# Design, Synthesis and Biological Evaluation of 1,3,5-Triazine Derivatives Targeting hA_1_ and hA_3_ Adenosine Receptor

**DOI:** 10.3390/molecules27134016

**Published:** 2022-06-22

**Authors:** Sujin Park, Yujin Ahn, Yongchan Kim, Eun Joo Roh, Yoonji Lee, Chaebin Han, Hee Min Yoo, Jinha Yu

**Affiliations:** 1 College of Pharmacy and Graduate School of Pharmaceutical Sciences, Ewha Womans University, Seoul 03760, Korea; sujinp@kist.re.kr (S.P.); 121521@kist.re.kr (C.H.); 2 Biomedical Research Institute, Korea Institute of Science and Technology (KIST), Seoul 02792, Korea; riskywing@gmail.com (Y.K.); r8636@kist.re.kr (E.J.R.); 3 Biometrology Group, Korea Research Institute of Standards and Science (KRISS), Daejeon 34113, Korea; yujinahn2202@gmail.com; 4 Department of Precision Measurement, University of Science and Technology (UST), Daejeon 34113, Korea; 5 College of Pharmacy, Chung-Ang University, Seoul 06974, Korea; yoonjilee@cau.ac.kr

**Keywords:** adenosine receptor, dual ligand, 1,3,5-triazine, molecular docking, antitumor agents

## Abstract

Adenosine mediates various physiological activities in the body. Adenosine receptors (ARs) are widely expressed in tumors and the tumor microenvironment (TME), and they induce tumor proliferation and suppress immune cell function. There are four types of human adenosine receptor (hARs): hA_1_, hA_2A_, hA_2B_, and hA_3_. Both hA_1_ and hA_3_ AR play an important role in tumor proliferation. We designed and synthesized novel 1,3,5-triazine derivatives through amination and Suzuki coupling, and evaluated them for binding affinities to each hAR subtype. Compounds **9a** and **11b** showed good binding affinity to both hA_1_ and hA_3_ AR, while **9c** showed the highest binding affinity to hA_1_ AR. In this study, we discovered that **9c** inhibits cell viability, leading to cell death in lung cancer cell lines. Flow cytometry analysis revealed that **9c** caused an increase in intracellular reactive oxygen species (ROS) and a depolarization of the mitochondrial membrane potential. The binding mode of 1,3,5-triazine derivatives to hA_1_ and hA_3_ AR were predicted by a molecular docking study.

## 1. Introduction

Extracellular adenosines are important signal transmitters and mediate various physiological activities in the body [1,2]. Human adenosine receptors (hARs) can be classified into four subtypes: hA_1_, hA_2A_, hA_2B_, and hA_3._ All four belong to the G protein-coupled receptor (GPCR) family, and each has a different pharmacological profile, tissue distribution, and function [3]. Although hARs have been known for a long time, new functions are continuously being discovered and novel ligands being developed.

hA_1_ AR is found in various tissues and cells and regulates many physiological activities in the body; for example, the activation of hA_1_ AR leads to bradycardia [4], inhibits neurotransmitter release [5], lipolysis [6], and renal excretion [7] and induces smooth muscle contraction [8]. The hA_1_ AR agonist has mainly been developed for treatment of cardiovascular diseases, such as heart failure, arrhythmia, and angina, or for neurological diseases, such as seizure, ischemia, and depression [9].

hA_3_ AR is also important for physiological signaling in the body. hA_3_ AR is expressed at a low level in most cells but is overexpressed in inflammatory and various neoplastic cells [10]. Therefore, it is an important drug target for developing therapeutic agents for glaucoma, stroke, asthma, inflammation, rheumatoid arthritis, and cancer [11].

Multiple studies have demonstrated that hA_1_ AR regulates the proliferation of tumor cells and that hA_1_ AR antagonists inhibit the proliferation and migration of tumor cells [12]. It is unclear exactly what function hA_3_ AR has in tumor cell proliferation and death [13,14]. Numerous publications have shown that hA_3_ AR is overexpressed in various types of cancer cells [15]. The activation of hA_3_ AR induces the growth of tumor cells by increasing VEGF, HIF-1, MMP-9, angiogenesis, migration, and proliferation [16]. At the same time, it decreases cell proliferation by induction of G-CSF and IL-2 in the immune system [17].

Polypharmacology, a concept that controls multiple targets with one drug, is a new paradigm in drug discovery that has recently received attention [18]. Compared with combination therapy, polypharmacology has higher safety and lower risk of drug–drug interaction [19]. hA_1_ and hA_3_ AR are good targets for multitarget drugs because among hARs, hA_1_ and hA_3_ AR are quite similar, with 49% sequence similarity [3]. In addition, the two are activated by the same endogenous ligand, adenosine, and the downstream signal also inhibits cyclic adenosine monophosphate (cAMP) production in the same way.

**Figure 1:**
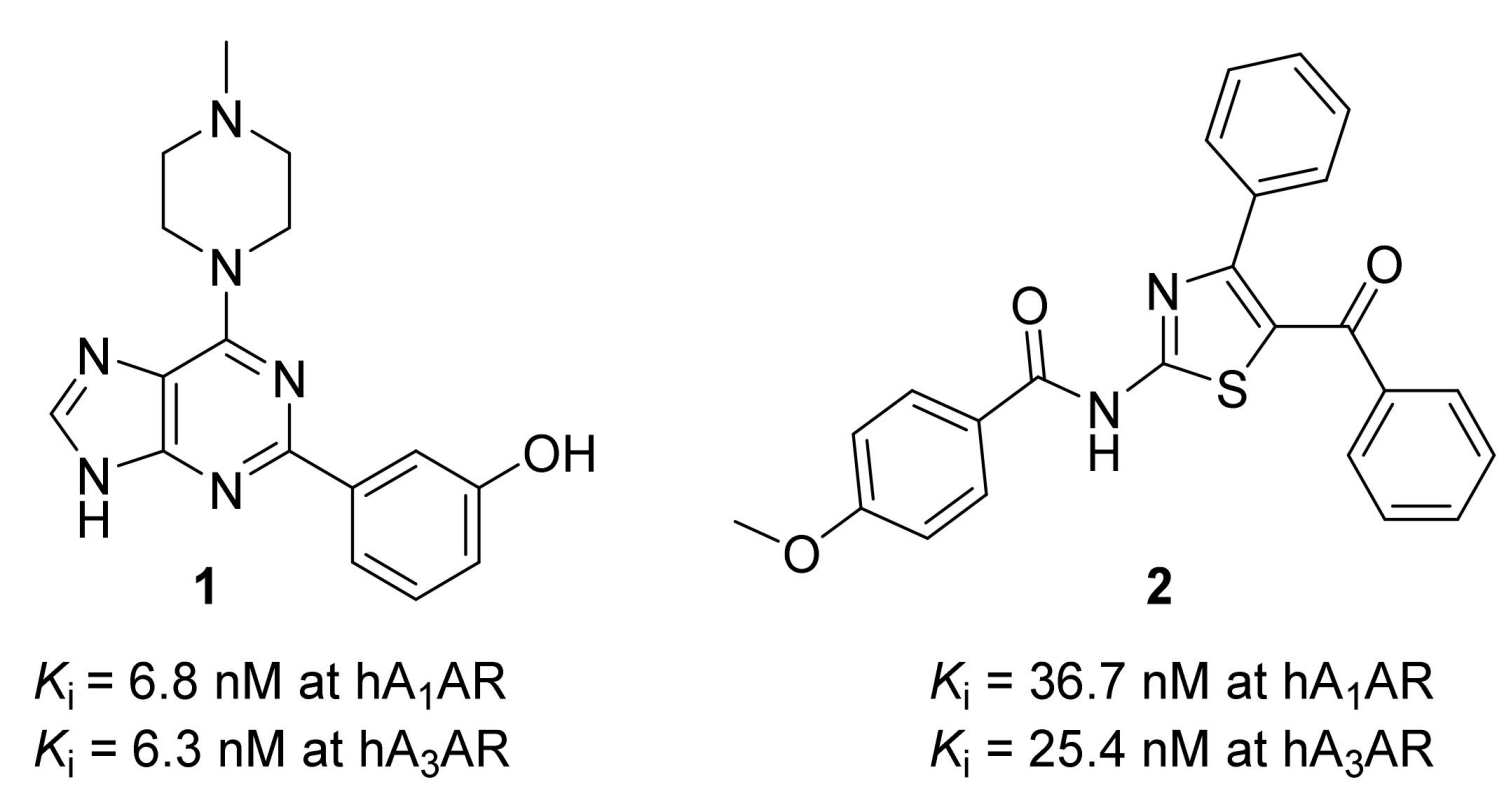
The structure of dual hA_1_–hA_3_ AR antagonists. AR: adenosine receptor.

Determining what alterations tumor cells undergo when simultaneously inhibiting both hA_1_ and hA_3_ AR that share a sub-signal transduction will be an important step in the development of anticancer drugs targeting adenosine receptors. Several studies on ligands that simultaneously regulate hA_1_ and hA_3_ AR have been published [20,21,22]. Compound **1** bearing a purine scaffold shows an inhibition constant (*K*_i_) of 6.8 and 6.3 nM, and compound **2** shows a *K*_i_ of 36.7 and 25.4 nM for hA_1_ and hA_3_ AR, respectively, indicating balanced binding (Figure 1).

**Figure 2:**
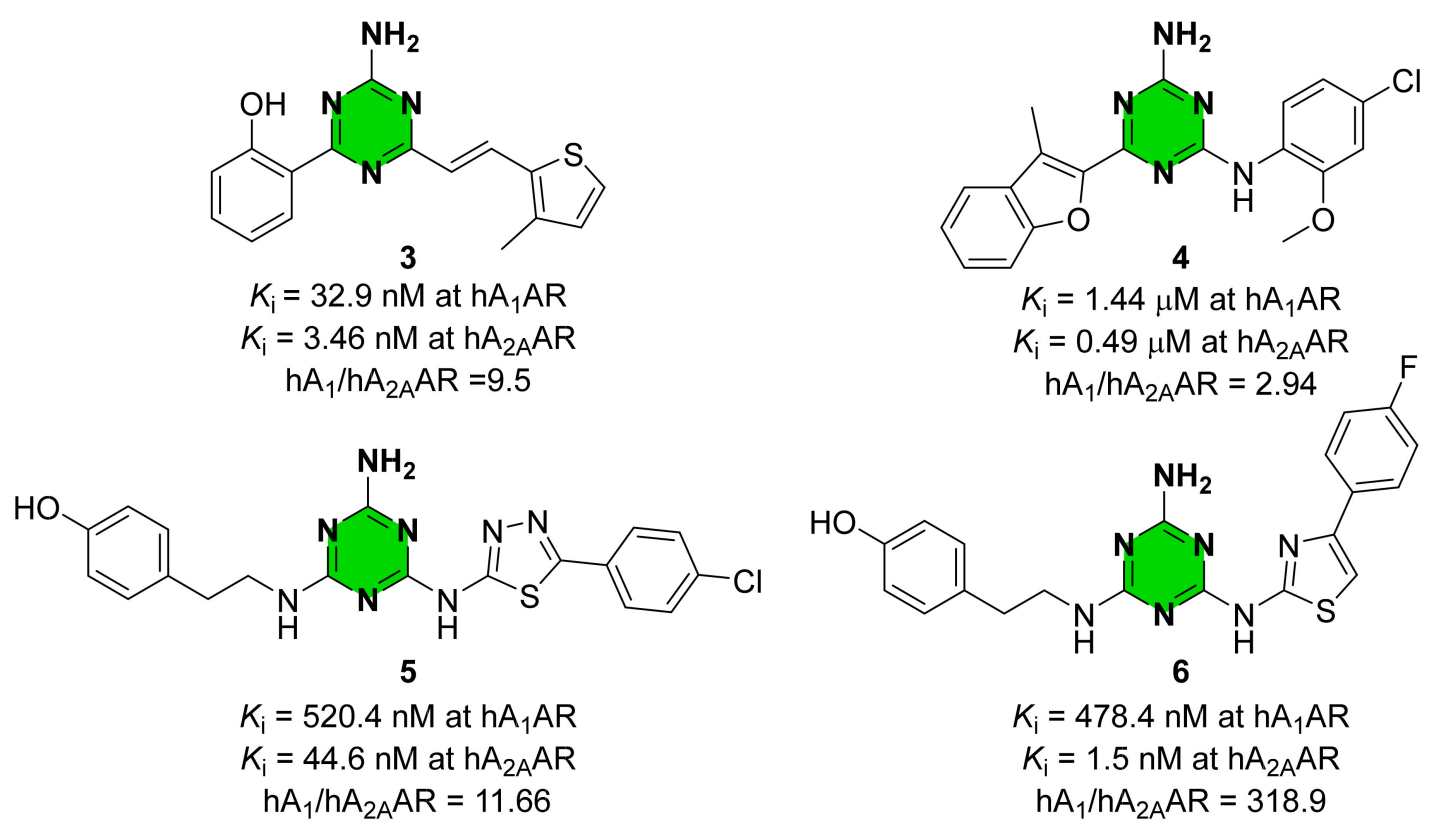
1,3,5-Triazine derivatives as hAR ligands. AR: adenosine receptor.

To develop a novel scaffold of the hAR ligand in non-xanthine ligands, we analyzed previously reported compounds targeting adenosine receptors. Langmead et al., in an effort to find a novel hit through a docking study, reported that compounds containing 1,3,5-triazine bind to hA_1_ and hA_2A_ AR (Figure 2) [23]. Compound **3** was found to be an hA_2A_ AR antagonist, with moderate selectivity (9.5-fold) against hA_1_ AR. Compound **4** was discovered through virtual screening and bound more potently compared to compound **3**, but the selectivity against hA_1_ AR was 2.94, which was lower compared to compound **3 [24]**. 

Compound **5** (1,3,5-triazine-thiadiazole) has been described as a potent hA_2A_ AR antagonist [25,26]. Compound **6** showed 319-fold selectivity against hA_2A_ AR compared to hA_1_ AR; however, the selectivity index (hA_1_ AR:hA_2A_ AR) of compound **5** was 11.66, indicating low selectivity. On the basis of previously published research, it was determined that derivatives of the 1,3,5-triazine scaffold were ligands that modulate adenosine receptors. In addition, the type of triazine substituents altered the subtype selectivity of adenosine receptors. This indicates that triazine modifications could be optimized to produce selective ligands for subtypes other than hA_2A_ AR. Therefore, we attempted to introduce various substitutions into 1,3,5-triazine in order to provide specific ligands for hA_1_ and hA_3_ AR.

In this study, we developed a series of 1,3,5-triazine derivatives that bind to hA_1_ and hA_3_ AR by substituting at the 2, 4, and 6 positions of the 1,3,5-triazine scaffold. A novel 2-amino-1,3,5-triazine derivative was designed by introducing substituents at the 4 and 6 positions of 1,3,5-triazine. The designed derivatives were synthesized, and their binding affinities to hARs were evaluated using a radioligand. The binding mode of 1,3,5-triazine derivatives were predicted by a molecular docking study using the crystal structure of the hA_1_ AR (PDB ID: 5N2S) and a homology model of the hA_3_ AR [27].

## 2. Results and Discussion

### 2.1. Synthesis of 1,3,5-Triazine Derivatives

**Scheme 1:**
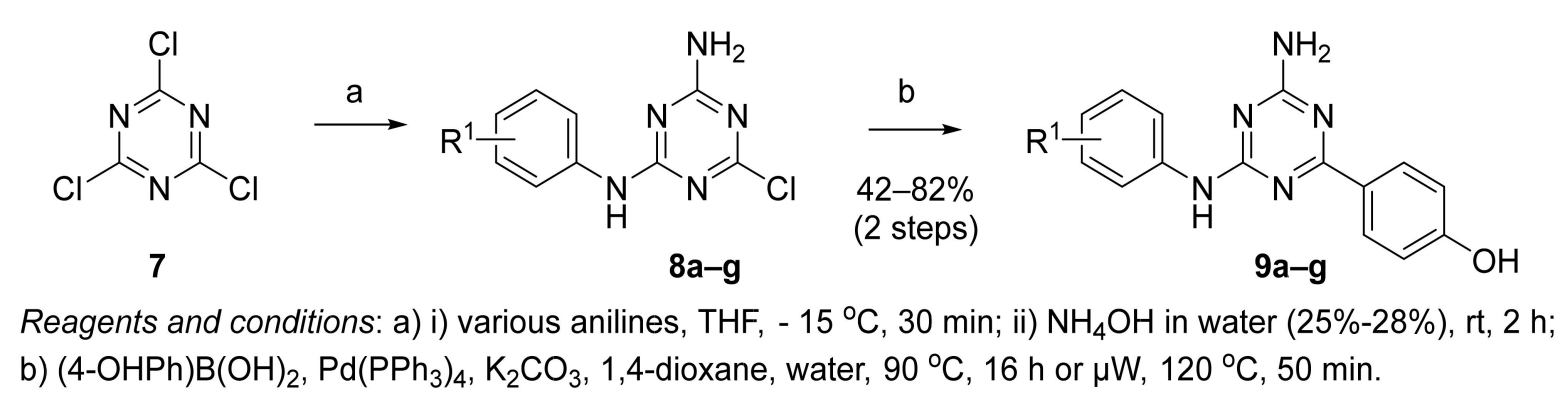
Synthesis of 2-(4-hydroxyphenyl)-4-amino-1,3,5-triazine derivatives. THF: tetrahydrofuran.

2-Amino-1,3,5-triazine derivatives were prepared as described in Scheme 1. Briefly, cyanuric chloride (**7**) was reacted with various types of anilines to obtain **8a–g**, followed by amination using aqueous ammonia (25–28% in water), synthesizing **9a–g**. Suzuki coupling with (4-hydroxyphenyl) boronic acid and **8a–g** in the presence of Pd(PPh_3_)_4_ yielded **9a–g** in moderate-to-good yield (42–82%).

**Scheme 2:**
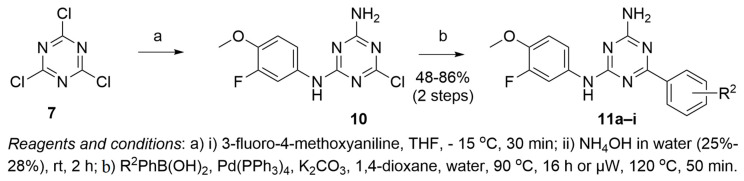
Synthesis of 6-(3-fluoro-4-methoxyaniline)-4-amino-1,3,5-triazine derivatives. THF: tetrahydrofuran.

Based on compound **8a**, we synthesized derivatives including 3-fluoro-4-methoxyaniline at the 4 position of 1,3,5-triazine through amination and Suzuki coupling (Scheme 2). Stepwise amination of cyanuric chloride using 3-fluoro-4-methoxyaniline and ammonium hydroxide yielded **10**, and Suzuki coupling with **10** and various boronic acids yielded the condensed compounds (**11a–i**).

### 2.2. Radioligand Binding Assays at Human Adenosine Receptors

All the synthesized compounds were screened with radioligand binding assays at hARs [28]. First, the percentage of inhibition was measured by treatment of each hAR subtype with 10 μM of each compound (Table 1). Except for **9c**, all compounds showed <90% inhibition at hA_2A_ and hA_2B_ AR. Compounds **9a–c**, **9e**, and **9g** showed >95% inhibition at hA_1_ AR, and compounds **9a**, **9c**, and **9d** showed >95% inhibition at hA_3_ AR. In addition, **9d** showed 69% inhibition at hA_1_ AR whereas it showed 95% inhibition at hA_3_ AR, showing significant selectivity. Compound **9c** showed >95% inhibition at all subtypes except hA_2B_ AR. Compound **9f** including 4-*N*-piperidine showed no binding with any of the subtypes, probably because the large piperidine interfered with binding to the hARs.

The binding affinities of compounds showing >95% inhibition were determined and are shown in Table 2. Compound **9a** with 3-fluoro-4-methoxyaniline showed the best binding affinity to hA_3_ AR (*K*_i_ = 55.5 nM) and good binding affinity to hA_1_ AR, with a 2.5-fold hA_1_ AR:hA_3_ AR selectivity index. Compound **9b** with 3,5-dimethoxyaniline also showed potent and selective binding affinity to hA_1_ AR (*K*_i_ = 69.7 nM). Compound **9c** with 3-methoxy-4-chloroaniline showed the best binding affinity to hA_1_ AR (*K*_i_ = 57.9 nM) and moderate binding affinity to hA_3_ AR (*K*_i_ = 661.1 nM). R^1^ substitution in the aniline appeared to be well tolerated by hA_1_ AR compared to hA_3_ AR. The compounds substituted with methoxy in aniline generally showed good binding affinity to hA_1_ AR, with higher affinity being obtained with compounds bearing meta-methoxy attached to the aniline core. Moreover, the compounds with methoxy substituted at the *para*-position of aniline showed the best binding affinity.

Since the most balanced binding to hA_1_ AR:hA_3_ AR was shown by **9a**, we developed a series of 3-fluoro-4-methoxyaniline derivatives **11a–i** for dual hA_1_–hA_3_ AR ligands. Various substituents were introduced at the *para*-position of phenyl to replace the hydroxyl group, and the percentage inhibition was evaluated at the four hAR subtypes with 10 μM of the synthesized compounds (Table 3). Compounds **11a** and **11b**, which included methoxy and fluorine at the *para*-position of benzene, respectively, displayed >90% inhibition at both hA_1_ and hA_3_ AR and low percentage inhibition at hA_2A_ and hA_2B_ AR. Compounds **11c–e** substituted with the electron-withdrawing groups OCF_3_, CF_3_, and CN, respectively, at the *para*-position of the phenyl core showed low percentage inhibition at all hAR subtypes. The compounds bearing a bulky group attached to the *para*-position of the phenyl core also showed low percentage inhibition at all hARs, indicating that the substituent at the *para*-position of the phenyl core is likely to be important for determining the binding affinity at hARs. Compounds **11h** and **11i** bearing two fluorine and nitro at the meta-position of the phenyl core, respectively, showed much lower percentage inhibition compared to **9a** and **11b**. We deduce that substituents at a position other than the *para*-position of the phenyl core negatively affect binding to hARs.

The binding affinities of **11a** and **11b** at hA_1_ and hA_3_ AR were determined and are shown in Table 4. Compound **11a** with methoxy instead of a hydroxyl group showed improved binding affinity to both hA_1_ and hA_3_ AR, about 2-fold at hA_1_ AR and 5-fold at hA_3_ AR, with an hA_1_ AR:hA_3_ AR selectivity index of 5.87. For **11b** bearing *para*-fluorine at the phenyl core, the binding affinity was less changed; however, the binding affinity to hA_1_ AR slightly improved, indicating an hA_1_ AR:hA_3_ AR selectivity index of 1.7.

### 2.3. cAMP Assay at hA_1_ and hA_3_ AR Adenosine Receptors

To determine whether 1,3,5-triazine scaffold derivatives behave as agonists or antagonists, we performed cAMP accumulation assays with **11a** and **11b** at hA_1_ and hA_3_ AR. We confirmed whether the test compounds behave as antagonists by measuring the concentration change in cAMP in the presence of a full agonist 5-*N*-ethylcarboxamido adenosine (NECA) using antagonist mode assays. We also confirmed whether the test compounds could behave as agonists by comparing the relative percentage activation of NECA using agonist mode assays. Both **11a** and **11b** showed 77% and 88% inhibition at hA_3_ AR in antagonist mode, respectively, and 17% and 6% percentage activation in agonist mode, respectively, indicating that **11a** and **11b** behave as antagonists at hA_3_ AR.

Compound **11b** showed 60% inhibition in antagonist mode and 23% activation in agonist mode, confirming that it acts as an antagonist and a partial agonist for hA_1_ AR. However, the assay results of **11a** at hA_1_ AR were interesting. The percentage inhibition and percentage activation of **11a** were 1% and 11% in antagonist mode and agonist mode, respectively, indicating that **11a** is neither an antagonist nor an agonist for hA_1_ AR. That is, **11a** binds to hA_1_ AR with high binding affinity, and the cAMP concentration does not change due to **11a** binding. Thus, additional experiments are required to determine which signaling transduction occurs after **11a** binds to hA_1_ AR.

### 2.4. Cell Viability of 1,3,5-Triazine Derivatives ***9a***–***c***, ***9g***, and ***11a***–***b***

**Figure 3:**
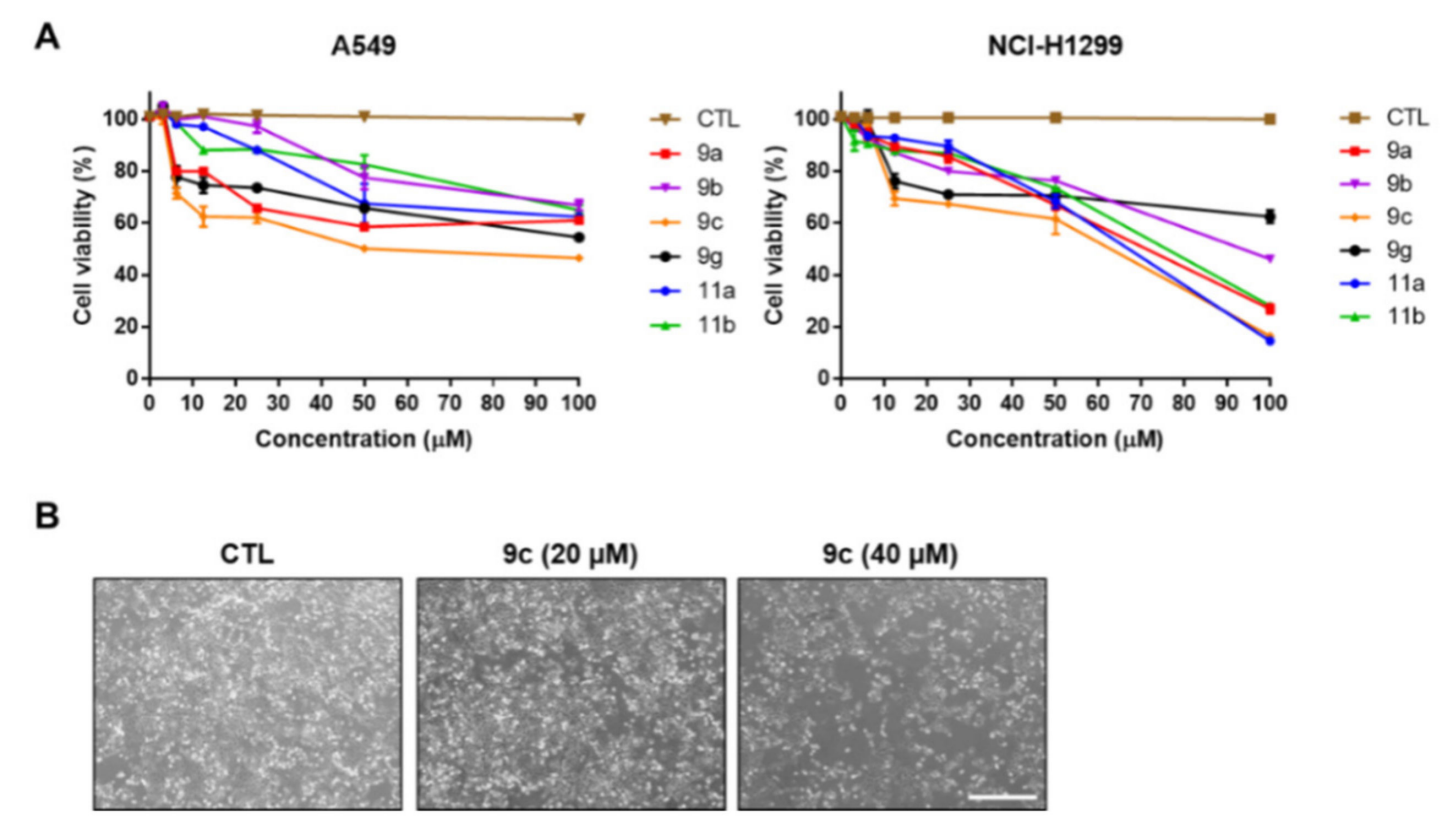
Compound **9c** decreases cell viability in lung cancer cell lines. (**A**) Cell viability as measured through MTS assays in which A549 and NCI-H1299 cells were exposed to various concentrations of the compounds. (**B**) Cell morphologies were taken after 48 h of treatment with **9c** (scale bar: 750 μm).

To test the effect of the compounds in terms of cell growth regulation in lung cancer cell lines such as A549 and NCI-H1299 cells, these cells were treated in 96-well plates for 48 h with the compounds at concentrations ranging from 0 μM to 100 μM. Viability of A549 and NCI-H1299 cells was lowered by treatment with 1,3,5-triazine derivatives **9a**–**c**, **9g**, and **11a**–**b** which showed good binding affinity at hA_1_ AR. However, compound **9a** and **11a**–**b**, which bound to hA_1_ and hA_3_ AR, exhibited relatively low cell viability, whereas compound **9c**, which had the highest binding affinity at hA_1_ AR among the derivatives, exhibited the greatest inhibitory effect. To assess lung cancer cell viability, a cell viability assay was performed, and the results indicated a significant decrease in cell viability to 59.9% in A549 cells treated with **9c** of 25 μM concentration, and to 68.8% in NCI-H1299 cells treated with **9c** of 25 μM concentration (Figure 3A). Therefore, we decided to perform an additional experiment with **9c**. Microscopic analysis of A549 cells treated with 20 μM and 40 μM of **9c** further showed that these cells exhibited gradual changes in cell growth in a dose-dependent manner relative to the concentration of **9c** (Figure 3B).

### 2.5. Compound ***9c***-Induced Intracellular ROS and Mitochondrial ROS

**Figure 4:**
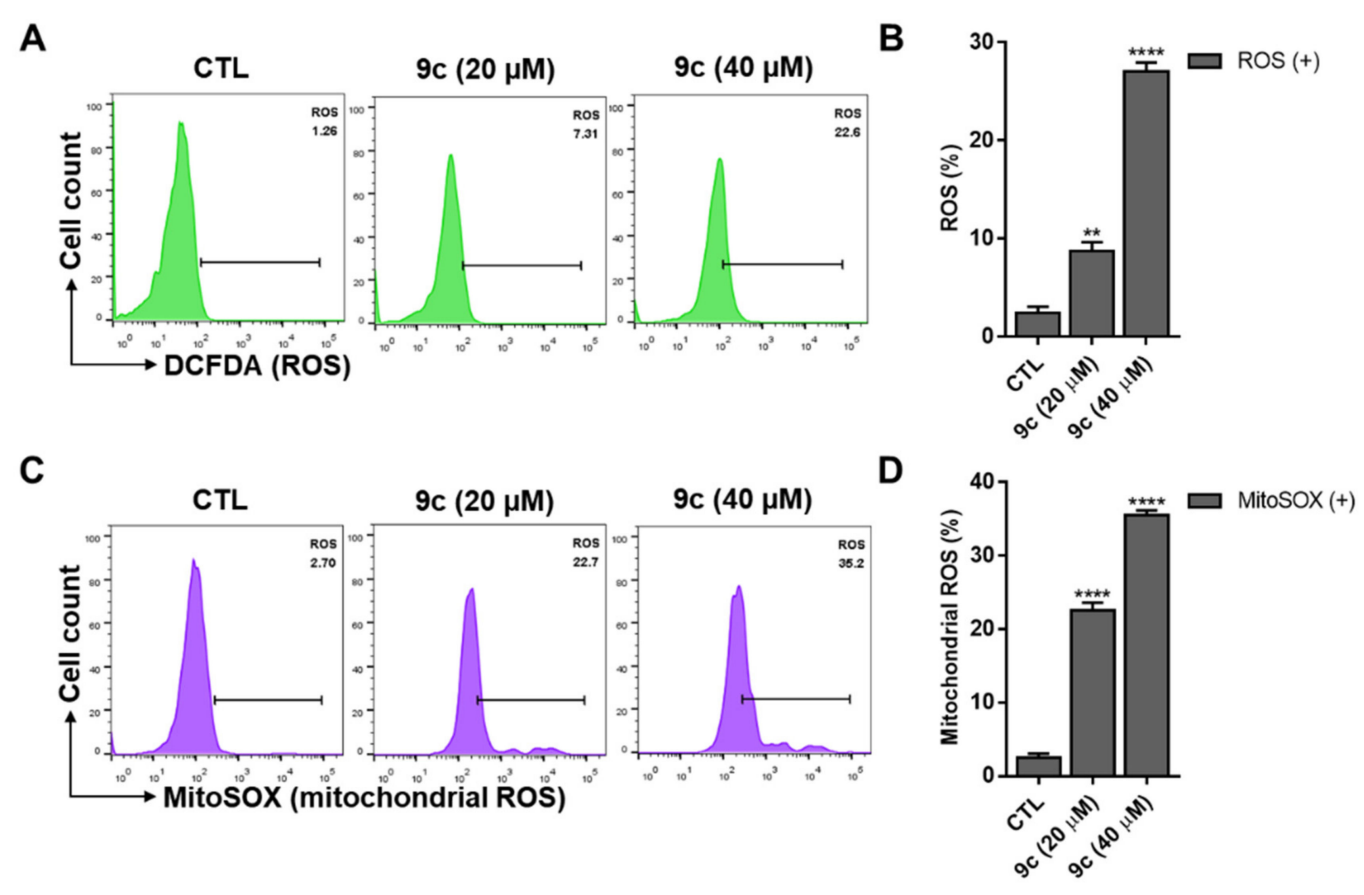
Compound **9c**-induced reactive oxygen species (ROS) generation in A549 cell lines. (**A**) Changes in ROS levels following treatment with 20 μM and 40 μM **9c**, respectively, for 48 h as measured using H_2_DCFDA and a flow cytometer. (**B**) Quantification of ROS levels. (**C**) A549 cells were treated with **9c** and mitochondrial ROS was indicated by MitoSOX Red. Flow cytometry was employed to analyze the A549 cells. (**D**) Quantification of mitochondrial ROS levels. Values indicate means ± SEM. (*n* = 3, ** *p* ≤ 0.01, **** *p* ≤ 0.0001).

Although reactive oxygen species (ROS) play an important role in regulating normal cellular processes, abnormal ROS levels contribute to the development of a variety of human diseases, including cancer. Because of their accelerated metabolism, cancer cells have higher ROS levels than normal cells [29]. Nevertheless, the high ROS content of cancer cells makes them more susceptible to oxidative stress-induced cell death, which can be used to target cancer cells selectively [30]. Flow cytometry was used to determine whether **9c** induces ROS generation in A549 cells by examining the results. The experiment was conducted with H_2_DCF-DA as a fluorescent probe. As shown in Figure 4A,B, A549 cells treated with **9c** of 20 μM and 40 μM concentrations exhibited highly increased ROS of 7.31% and 22.6%, respectively. For comparison, the control sample exhibited a ROS level of 1.26%. Next, we determined the effect of **9c** in regulating mitochondrial ROS. Our results show the **9c**-treated A549 cells to have significantly increased mitochondrial ROS levels (Figure 4C,D). These data indicate that **9c** treatment makes lung cancer cells more sensitive to oxidative stress and makes them more vulnerable to ROS-mediated cell death.

### 2.6. Compound ***9c***-Induced Mitochondrial Membrane Dysfunction

**Figure 5:**
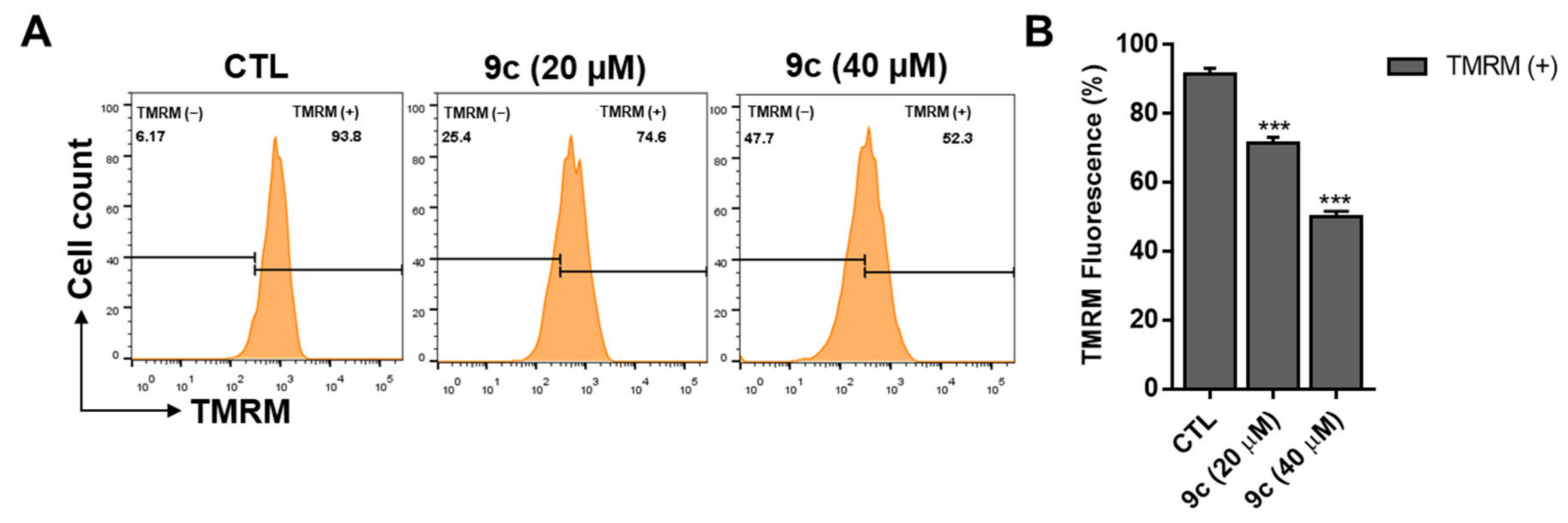
**9c** decreases mitochondrial membrane potential in the A549 cell line. (**A**) Effects of treatment with 20 μM and 40 μM **9c** for 48 h on the mitochondrial membrane potential of A549 cells as measured using the TMRM reagent (100 nM) and a flow cytometer. (**B**) Quantification of mitochondrial membrane potential. Values indicate the means ± SEM. (*n* = 3, *** *p* ≤ 0.001).

ROS-induced oxidative stress can cause the rapid depolarization of the inner mitochondrial membrane potential (ΔΨm) and, as a result, impairment of oxidative phosphorylation [31]. Using tetramethylrhodamine methyl ester (TMRM) measurements, we investigated how mitochondrial membrane potential was affected by **9c**. A549 cells were exposed to **9c** following incubation with TMRM. Afterward, flow cytometry was employed to calculate the intensity of TMRM binding in the healthy membrane. Figure 5A,B show that, compared to the control value of 93.8%, the TMRM-positive intensity dramatically decreased to 74.6.% at 20 μM, and to 52.3% at 40 μM, respectively. The results suggest that **9c** causes the A549 cell’s mitochondrial membrane to depolarize, which leads to dysfunction.

### 2.7. Compound ***9c*** Effects on Lung Cancer Cell Death

**Figure 6:**
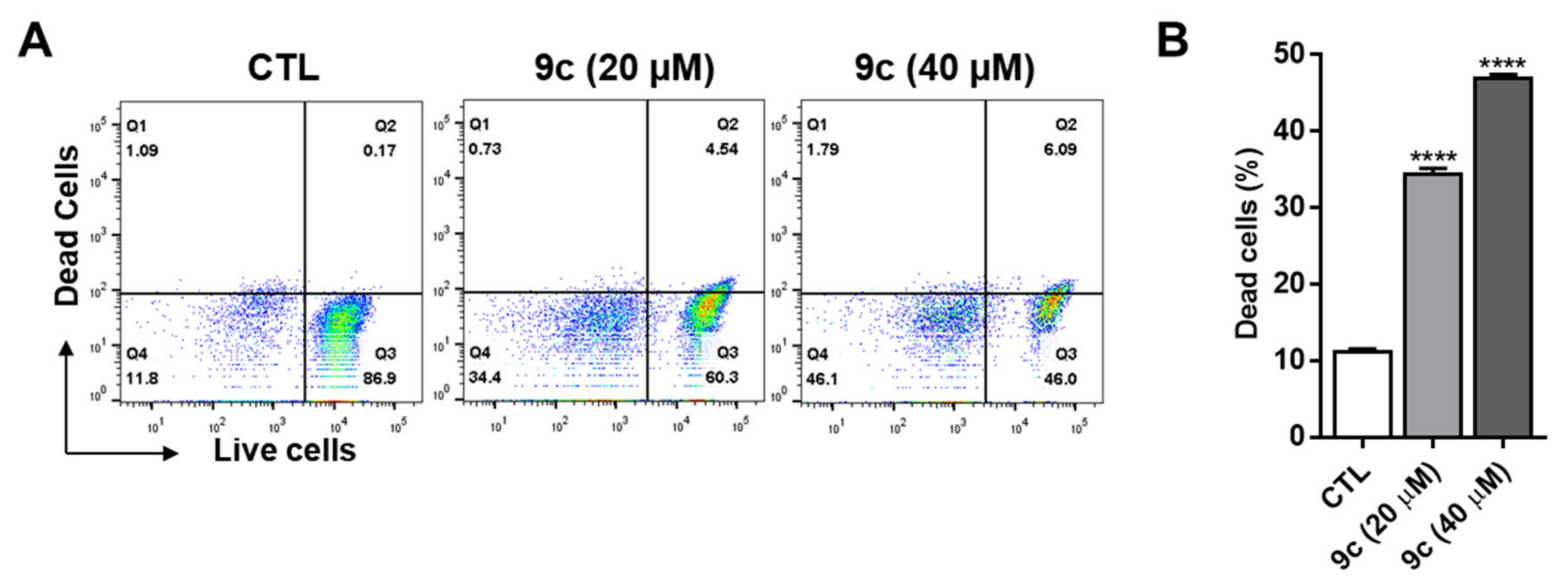
Compound **9c**-induced cytotoxic effects on lung cancer cell lines. (**A**) Live and dead assay performed using a flow cytometer following **9c** treatment of A549 cells. (**B**) Quantification of dead cells. Values indicate means ± SEM. (*n* = 3, **** *p* ≤ 0.0001).

A live cell assay and a dead cell assay were carried out using a mixture of two fluorescent dyes: calcein (green dye for live cells) and ethidium homodimer-1 (EthD-1, red dye for dead cells). The cells were washed and stained with calcein and EthD-1 before conducting imaging fluorescence microscopy and flow cytometry experiments. When treated with 20 μM **9c**, we observed a drastic decrease in the number of live cells (green) and a slight increase in the number of dead cells (red). This was supported by the flow cytometry results, which indicated an approximate increase in the number of dead cells (compared to the control group) by 34.4% following treatment with 20 μM **9c**, and by 46.1% following treatment with 40 μM **9c** (Figure 6A,B), respectively. As a result, our findings suggest that **9c** significantly reduces the viability of lung cancer cell lines.

### 2.8. Molecular Docking Study of 1,3,5-Triazine Derivatives

**Figure 7:**
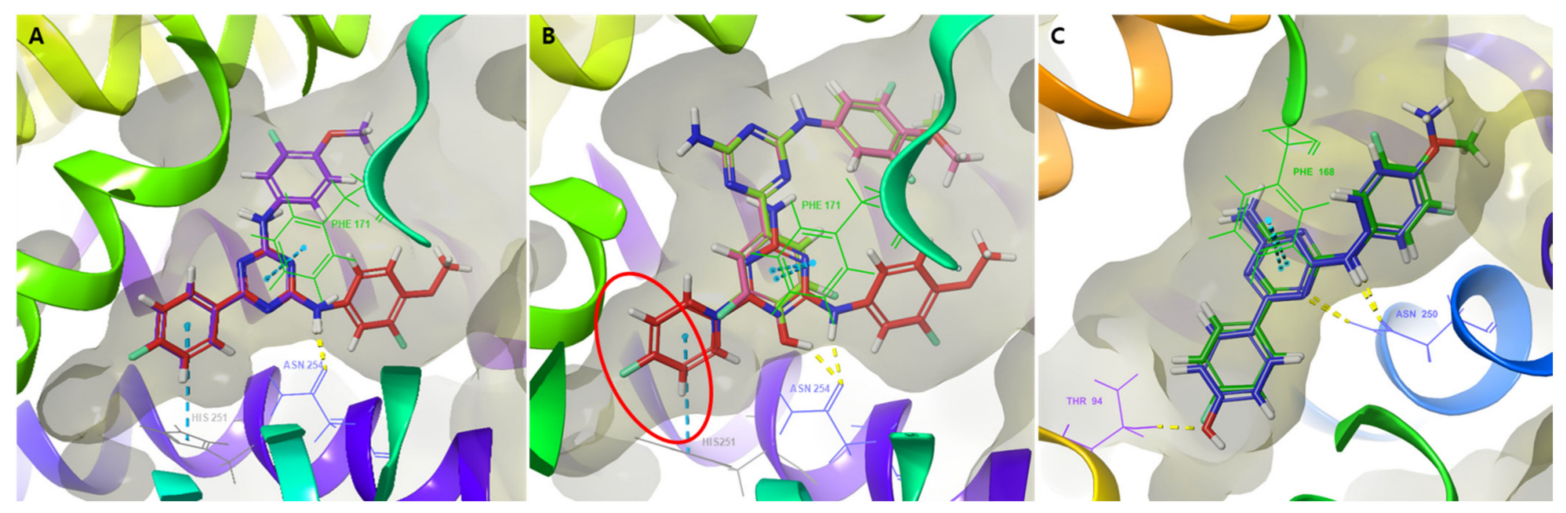
(**A**) Superimposition of two predicted docking poses in hA_1_ AR of **11b**. (**B**) Superimposition of docking poses in hA_1_ AR of **11b** (red carbon atom), **11h** (salmon pink carbon atom), and **11i** (yellow-green carbon atom). The narrow binding pocket for *para*-phenol ring of **11b** is highlighted with a red circle. (**C**) Superimposition of docking poses in hA_3_ AR of **9a** (blue carbon atom) and **11b** (green carbon atom). Hydrogen-bonding and π–π stacking interaction are pictured as yellow and cyan dashed lines, respectively.

We attempted molecular docking to investigate how the triazine derivatives bind to hA_1_ and hA_3_ AR. Initially, the binding mode of **11b** to hA_1_ AR was predicted using x-ray structure (PDB; 5N2S). Consequently, two docking poses of **11b** in hA_1_ AR were proposed and depicted in Figure 7A. There was no significant difference between the two binding mode docking scores, −9.100 (red) and −8.624 (violet). **11b** was stabilized by π–π interactions between the triazine and F171^5.29^ in the two docking poses. By contrast, N254^6.5^ formed hydrogen bonds with the nitrogen of aniline in the red-binding mode, or nitrogen of amino in the purple-binding mode, respectively. The aniline group of **11b** was oriented toward the augmented TM2 region in hA_1_ AR, adopting a purple-binding pose. Docking was also performed on **9a** and **9c**, which both exhibited strong binding affinity in hA_1_ AR and were both predicted to bind in the same manner as **11b**. (Supplementary Materials, Figure S3). In addition, **11h** and **11i** with a substituent at the *meta* position of the phenyl ring were also applied to a docking study (Figure 7B). The phenyl rings of **11h** and **11i** were predicted to occupy the binding pocket at which triazine of **11b** was located. Since the binding pocket for phenyl ring of **11b** was narrow (Figure 7B—red circle), it can be inferred that *meta*-substituents on a phenyl ring of triazine derivatives interfered with the binding to the receptor, which explained why **11h** and **11i** were inactive at hA_1_ AR.

We used the previously published homology model of hA_3_ AR for docking [27], since the x-ray crystal structure of hA_3_ AR has not yet been determined. The ligand used to generate the homology model is structurally distinct from triazine derivatives; therefore, induced-fit docking was utilized to predict the binding mode of **11b** in hA_3_ AR. The N250^6.55^ of hA_3_ AR generated hydrogen bonds with the nitrogen of aniline and triazine of **11b**, and its triazine formed a π–π interaction with F168^5.29^ (Figure 7C). This was consistent with the binding mode in hA_1_ AR, and the docking study explained how **11b** binds to both hA_1_ and hA_3_ AR. **9a** was predicted to bind in the same manner as **11b** on the hA_3_ AR model, which was generated from the **11b** induced-fit docking (Figure 7C).

## 3. Materials and Methods

### 3.1. Chemical Synthesis

#### 3.1.1. General Chemical Synthesis

Reagents and solvents were purchased from commercial suppliers (Sigma-Aldrich, Seoul, Korea; Acros Organics, Seoul, Korea; TCI, Seoul, Korea, etc.) and used as provided, unless indicated otherwise. All reactions except Suzuki coupling that used boronic acids with palladium catalysts in a microwave were performed in a round-bottom flask under a nitrogen atmosphere with stirring at room temperature. Reactions were monitored with analytical thin-layer chromatography (TLC) using glass sheets pre-coated with silica gel 60 F_254_ (Merck, Darmstadt, Germany), with visualization under ultraviolet (UV) light (254 nm).

Proton nuclear magnetic resonance (^1^H-NMR) spectra of the compounds dissolved in CDCl_3_, deuterated dimethyl sulfoxide (DMSO-*d*_6_), or D_2_O, were recorded on a Bruker Avance 400 MHz (Bruker Corporation, Billerica, MA, USA). The chemical shifts were expressed as δ-values in parts per million (ppm) using residual solvent peaks (CDCl_3_: 1H, 7.26 ppm; DMSO: 1H, 2.50 ppm) as a reference. Coupling constants were given in hertz (Hz). The peak patterns are indicated by the following abbreviations: bs = broad singlet, d = doublet, dd = doublet of doublet, m = multiplet, q = quadruplet, s = singlet, and t = triplet. High-resolution spectra were obtained using Waters ACQUITY UPLC^®^ BEH C18 1.7μ-Q-TOF SYNAPT G2-Si (Waters Corporation, Milford, MA, USA) high-resolution mass spectrometry (HRMS). Column chromatography was performed on silica gel 60 (230–400 mesh). Eluent solvents for all chromatographic methods are noted as appropriate mixed solvents with given volume-to-volume ratios.

#### 3.1.2. General Procedure for the Synthesis of **8a**–**g**

A cyanuric chloride (**7**) solution (1 equiv) in tetrahydrofuran (THF) was stirred and then cooled to −15 °C. Aniline (1 equiv) was added, and the mixture was stirred at the −15 °C for 0.5–1 h. Under TLC monitoring, ammonium hydroxide solution (25–28% NH_3_ in water) was added, and the mixture was stirred at room temperature for 1–2 h. Finally, the solvent was removed under reduced pressure, and the resulting solid was collected by filtration and dried to obtain the desired product.

#### 3.1.3. General Procedure for the Synthesis of **9a**–**g**

Intermediates **8a**–**g** (1 equiv), (4-hydroxyphenyl)boronic acid (2 equiv), tetrakis(triphenylphosphine)palladium (5 mol%), potassium carbonate (2 equiv), and a 4:1 dioxane:water mixture were added to a microwave tube. The mixture was heated to 120 °C for 1 h under microwave irradiation and then filtered through Celite with ethyl acetate as an eluent solvent. Next, the filtrate was washed with water and extracted with ethyl acetate. The combined organic layers were washed with brine, dried over anhydrous Na_2_SO_4_, filtered, and concentrated in vacuo. Finally, the resulting residue was purified by column chromatography.

##### Compound **9a**

The 4-{4-amino-6-[(3-fluoro-4-methoxyphenyl)amino]-1,3,5-triazin-2-yl}phenol (**9a**) yield was 62%: ^1^H-NMR (400 MHz, DMSO-*d*_6_) δ 9.99 (s, 1H), 9.41 (s, 1H), 8.15 (d, *J* = 8.8 Hz, 2H), 7.87 (d, *J* = 12.5 Hz, 1H), 7.46 (d, *J* = 8.2 Hz, 1H), 7.09 (t, *J* = 9.4 Hz, 1H), 7.00 (s, 2H), 6.85 (d, *J* = 8.8 Hz, 2H), 3.80 (s, 3H); ^13^C-NMR (100 MHz, DMSO-*d*_6_) δ 170.01, 166.98, 164.32, 160.61, 150.95 (d, *J* = 240.5 Hz); 141.87 (d, *J* = 10.9 Hz); 133.81 (d, *J* = 9.8 Hz); 129.71, 127.48, 115.44, 114.99, 114.02 (d, *J* = 2.9 Hz); 108.22 (d, *J* = 22.9 Hz); 56.26; HRMS (ES+): *m*/*z* calculated for C_16_H_14_FN_5_O_2_: 328.1210 [M + H]^+^; found 328.1221.

##### Compound **9b**

The 4-{4-amino-6-[(3,5-dimethoxyphenyl)amino]-1,3,5-triazin-2-yl}phenol (**9b**) yield was 56%: ^1^H-NMR (400 MHz, DMSO-*d*_6_) δ 10.02 (s, 1H), 9.33 (s, 1H), 8.17 (d, *J* = 8.8 Hz, 2H), 7.15 (d, *J* = 2.3 Hz, 2H), 7.00 (s, 2H), 6.84 (d, *J* = 8.7 Hz, 2H), 6.14 (t, *J* = 2.2 Hz, 1H), 3.74 (s, 6H); ^13^C-NMR (100 MHz, DMSO-*d*_6_) δ 170.02, 166.97, 164.55, 160.66, 160.32, 141.84, 129.70, 127.47, 114.99, 98.05, 94.00, 55.02; HRMS (ES+): *m*/*z* calculated for C_17_H_17_N_5_O_3_: 340.1410 [M + H]^+^; found 340.1436.

##### Compound **9c**

The 4-{4-amino-6-[(4-chloro-3-methoxyphenyl)amino]-1,3,5-triazin-2-yl}phenol (**9c**) yield was 55%: ^1^H-NMR (400 MHz, DMSO-*d*_6_) δ 10.04 (s, 1H), 9.53 (s, 1H), 8.18 (d, *J* = 8.8 Hz, 2H), 7.92 (d, *J* = 2.2 Hz, 1H), 7.44–7.24 (m, 2H), 7.06 (s, 2H), 6.85 (d, *J* = 8.8 Hz, 2H), 3.88 (s, 3H); ^13^C-NMR (100 MHz, DMSO-*d*_6_) δ 170.13, 166.97, 164.45, 160.71, 154.27, 140.50, 129.78, 129.27, 127.41, 115.04, 113.27, 112.27, 104.51, 55.86; HRMS (ES+): *m*/*z* calculated for C_16_H_14_ClN_5_O_2_: 344.0914 [M + H]^+^; found 344.0938.

##### Compound **9d**

The 4-(4-amino-6-{[3-(trifluoromethyl)phenyl]amino}-1,3,5-triazin-2-yl)phenol (**9d**) yield was 82%: ^1^H-NMR (400 MHz, DMSO-*d*_6_) δ 10.05 (s, 1H), 9.75 (s, 1H), 8.35 (s, 1H), 8.18 (d, *J* = 8.9 Hz, 2H), 8.07 (d, *J* = 6.6 Hz, 1H), 7.52 (t, *J* = 7.8 Hz, 1H), 7.30 (d, *J* = 7.6 Hz, 1H), 7.08 (s, 2H), 6.85 (d, *J* = 8.9 Hz, 2H); ^13^C-NMR (100 MHz, DMSO-*d*_6_) δ 170.23, 167.07, 164.58, 160.83, 141.08, 130.03–128.68 (m); 129.56, 127.30, 124.38 (q, *J* = 272.3 Hz); 123.08, 117.86, 117.82, 115.83, 115.08; HRMS (ES+): *m*/*z* calculated for C_16_H_12_F_3_N_5_O: 348.1072 [M + H]^+^; found 348.1100.

##### Compound **9e**

The 4-{4-amino-6-[(3,5-dimethylphenyl)amino]-1,3,5-triazin-2-yl}phenol (**9e**) yield was 69%: ^1^H-NMR (400 MHz, DMSO-*d*_6_) δ 9.99 (s, 1H), 9.22 (s, 1H), 8.16 (d, *J* = 8.7 Hz, 2H), 7.46 (s, 2H), 6.93 (s, 2H), 6.84 (d, *J* = 8.8 Hz, 2H), 6.62 (s, 1H), 2.26 (s, 6H); ^13^C-NMR (100 MHz, DMSO-*d*_6_) δ 170.00, 167.06, 164.57, 160.60, 139.96, 137.24, 129.74, 127.59, 123.47, 117.73, 115.01, 21.28; HRMS (ES+): *m*/*z* calculated for C_17_H_17_N_5_O: 308.1511 [M + H]^+^; found 308.1553.

##### Compound **9f**

The 4-(4-amino-6-{[4-(piperidin-1-yl)phenyl]amino}-1,3,5-triazin-2-yl)phenol (**9f**) yield was 58%: ^1^H-NMR (400 MHz, DMSO-*d*_6_) δ 9.99 (s, 1H), 9.13 (s, 1H), 8.15 (d, *J* = 8.8 Hz, 2H), 7.60 (d, *J* = 9.0 Hz, 2H), 7.11–6.49 (m, 6H), 3.22–2.86 (m, 4H), 1.79–1.58 (m, 4H), 1.57–1.42 (m, 2H); ^13^C-NMR (100 MHz, DMSO-*d*_6_) δ 169.87, 167.05, 164.41, 160.49, 147.32, 131.82, 129.70, 127.69, 121.22, 116.33, 114.94, 50.43, 25.44, 23.89; HRMS (ES+): *m*/*z* calculated for C_20_H_22_N_6_O: 363.1933 [M + H]^+^; found 363.1973.

##### Compound **9g**

The 4-{4-amino-6-[(2,4-dimethylphenyl)amino]-1,3,5-triazin-2-yl}phenol (**9g**) yield was 45%: ^1^H-NMR (400 MHz, DMSO-*d*_6_) δ 9.94 (s, 1H), 8.57 (s, 1H), 8.09 (d, *J* = 8.6 Hz, 2H), 7.27 (d, *J* = 7.9 Hz, 1H), 7.03 (s, 1H), 6.98 (dd, *J* = 8.2, 2.3 Hz, 1H), 6.81 (d, *J* = 8.7 Hz, 2H), 6.75 (s, 2H), 2.27 (s, 3H), 2.18 (s, 3H); ^13^C-NMR (100 MHz, DMSO-*d*_6_) δ 169.88, 167.25, 165.45, 160.40, 134.67, 133.92, 133.05, 130.70, 129.62, 127.66, 126.36, 114.86, 20.50, 18.06; HRMS (ES+): *m*/*z* calculated for C_17_H_17_N_5_O: 308.1511 [M + H]^+^; found 308.1548.

#### 3.1.4. Procedure for the Synthesis of Intermediate **10**

A cyanuric chloride (7) solution (1 equiv) in tetrahydrofuran was stirred and then cooled to −15 °C. 3-Fluoro-4-methoxyaniline (1 equiv) was added, and the mixture was stirred at −15 °C for 0.5–1 h. Under TLC monitoring, ammonium hydroxide solution (25–28% NH_3_ in water) was added, and the mixture was stirred at room temperature for 1–2 h. Finally, the solvent was removed under reduced pressure, and the resulting solid was collected by infiltration and dried to obtain the desired product.

#### 3.1.5. General Procedure for the Synthesis of **11a**–**i**

Intermediate **10** (1 equiv), R^2^-phenylboronic acid (2 equiv), tetrakis(triphenyl phosphine)palladium (5 mol%), potassium carbonate (2 equiv), and a 4:1 dioxane:water mixture were added to a microwave tube. The mixture was heated to 120 °C for 1 h under microwave irradiation and then filtered through Celite with ethyl acetate as an eluent solvent. Next, the filtrate was washed with water and extracted with ethyl acetate. The combined organic layers were washed with brine, dried over anhydrous Na_2_SO_4_, filtered, and concentrated in vacuo. Finally, the resulting residue was purified by column chromatography.

##### Compound **11a**

The *N*^2^-(3-fluoro-4-methoxyphenyl)-6-(4-methoxyphenyl)-1,3,5-triazine-2,4-diamine (compound **11a**) yield was 76%: ^1^H-NMR (400 MHz, CDCl_3_) δ 8.33 (d, *J* = 8.9 Hz, 2H), 7.69 (dd, *J* = 13.2, 2.7 Hz, 1H), 7.13 (d, *J* = 8.4 Hz, 1H), 7.03 (s, 1H), 7.01–6.87 (m, 3H), 5.23 (s, 2H), 3.89 (d, *J* = 5.7 Hz, 6H); ^13^C-NMR (100 MHz, DMSO-*d*_6_) δ 169.81, 167.02, 164.33, 162.01, 150.96 (d, *J* = 240.5 Hz); 141.94 (d, *J* = 10.9 Hz); 133.73 (d, *J* = 9.8 Hz); 129.54, 129.05, 115.51, 114.01 (d, *J* = 2.9 Hz); 113.66, 108.29 (d, *J* = 22.9 Hz); 56.26, 55.33; HRMS (ES+): *m*/*z* calculated for C_17_H_16_FN_5_O_2_: 342.1361 [M + H]^+^; found 342.1368.

##### Compound **11b**

The *N*^2^-(3-fluoro-4-methoxyphenyl)-6-(4-fluorophenyl)-1,3,5-triazine-2,4-diamine (**11b**) yield was 81%: ^1^H-NMR (400 MHz, CDCl_3_) δ 8.47–8.30 (m, 2H), 7.67 (dd, *J* = 13.2, 2.7 Hz, 1H), 7.21–7.06 (m, 3H), 7.04 (s, 1H), 6.94 (t, *J* = 9.0 Hz, 1H), 5.25 (s, 2H), 3.90 (s, 3H); ^13^C-NMR (100 MHz, DMSO-*d*_6_) δ 169.21, 167.06, 164.35, 164.27 (d, *J* = 248.9 Hz); 150.95 (d, *J* = 240.9 Hz); 142.10 (d, *J* = 10.9 Hz); 133.51 (d, *J* = 9.8 Hz); 133.22 (d, *J* = 2.9 Hz); 130.17 (d, *J* = 9.1 Hz); 115.70, 115.31 (d, *J* = 21.4 Hz); 114.00 (d, *J* = 2.9 Hz); 108.44 (d, *J* = 22.9 Hz); 56.25; HRMS (ES+): *m*/*z* calculated for C_16_H_13_F_2_N_5_O: 328.1010 [M − H]^−^; found 328.1013.

##### Compound **11c**

The *N*^2^-(3-fluoro-4-methoxyphenyl)-6-[4-(trifluoromethoxy)phenyl]-1,3,5-triazine-2, 4-diamine (**11c**) yield was 84%: ^1^H-NMR (400 MHz, CDCl_3_) δ 8.40 (d, *J* = 8.9 Hz, 2H), 7.67 (dd, *J* = 13.2, 2.6 Hz, 1H), 7.30 (d, *J* = 7.9 Hz, 2H), 7.13 (d, *J* = 8.6 Hz, 1H), 7.01 (s, 1H), 6.95 (t, *J* = 9.1 Hz, 1H), 5.25 (s, 2H), 3.90 (s, 3H); ^13^C-NMR (100 MHz, CDCl_3_) δ 167.37, 164.89, 152.25 (d, *J* = 244.9 Hz); 152.06, 144.15 (d, *J* = 10.6 Hz); 134.81 (d, *J* = 4.9 Hz); 131.87 (d, *J* = 10.6 Hz); 131.32–118.83 (m); 130.30, 120.51, 116.30 (d, *J* = 3.6 Hz); 113.88 (d, *J* = 2.9 Hz); 110.30, 110.07, 56.82; HRMS (ES+): *m*/*z* calculated for C_17_H_13_F_4_N_5_O_2_: 394.0927 [M − H]^−^; found 394.0929.

##### Compound **11d**

The *N*^2^-(3-fluoro-4-methoxyphenyl)-6-[4-(trifluoromethyl)phenyl]-1,3,5-triazine-2,4-diamine (**11d**) yield was 48%: ^1^H-NMR (400 MHz, chloroform-*d*) δ 8.50 (d, *J* = 8.1 Hz, 2H), 7.74 (d, *J* = 8.3 Hz, 2H), 7.66 (dd, *J* = 13.0, 2.5 Hz, 1H), 7.15 (d, *J* = 8.3 Hz, 1H), 6.96 (t, *J* = 9.0 Hz, 1H), 5.47 (s, 2H), 3.91 (s, 3H); ^13^C-NMR (100 MHz, DMSO-*d*_6_) δ 168.94, 167.11, 164.35, 150.94 (d, *J* = 241.0 Hz); 142.21 (d, *J* = 11.0 Hz); 140.68, 133.34 (d, *J* = 9.5 Hz); 131.17 (d, *J* = 32.3 Hz); 128.42, 125.36 (d, *J* = 3.9 Hz); 124.15 (d, *J* = 272.2 Hz); 115.78, 114.00 (d, *J* = 2.9 Hz); 108.51 (d, *J* = 22.4 Hz); 56.25; HRMS (ES+): *m*/*z* calculated for C_17_H_13_F_4_N_5_O: 378.0978 [M − H]^−^; found 378.0970.

##### Compound **11e**

The 4-{4-amino-6-[(3-fluoro-4-methoxyphenyl)amino]-1,3,5-triazin-2-yl}benzonitrile (**11e**) yield was 85%: ^1^H-NMR (400 MHz, DMSO-*d*_6_) δ 9.67 (s, 1H), 8.41 (d, *J* = 8.8 Hz, 2H), 8.00 (d, *J* = 8.7 Hz, 2H), 7.88 (s, 1H), 7.44 (d, *J* = 8.8 Hz, 1H), 7.32 (s, 2H), 7.11 (t, *J* = 9.4 Hz, 1H), 3.81 (s, 3H); ^13^C-NMR (100 MHz, DMSO-*d*_6_) δ 168.75, 167.08, 164.33, 150.93 (d, *J* = 240.9 Hz); 142.25 (d, *J* = 10.9 Hz); 141.08, 133.25 (d, *J* = 9.8 Hz); 132.46, 128.32, 118.60, 115.83, 113.98 (d, *J* = 2.9 Hz); 113.54, 108.56 (d, *J* = 22.5 Hz); 56.24; HRMS (ES+): *m*/*z* calculated for C_17_H_13_FN_6_O: 335.1057 [M − H]^−^; found 335.1051.

##### Compound **11f**

The methyl-4-{4-amino-6-[(3-fluoro-4-methoxyphenyl)amino]-1,3,5-triazin-2-yl}benzoate (**11f**) yield was 71%: ^1^H-NMR (400 MHz, chloroform-*d*) δ 8.42 (d, *J* = 8.8 Hz, 2H), 8.13 (d, *J* = 8.8 Hz, 2H), 7.68 (dd, *J* = 13.1, 2.6 Hz, 1H), 7.14 (d, *J* = 8.4 Hz, 1H), 7.04 (s, 1H), 6.95 (t, *J* = 9.0 Hz, 1H), 5.27 (s, 2H), 3.95 (s, 3H), 3.90 (s, 3H); ^13^C-NMR (100 MHz, DMSO-*d*_6_) δ 169.27, 167.11, 165.91, 164.33, 150.94 (d, *J* = 241.0 Hz); 142.15 (d, *J* = 10.6 Hz); 141.11, 133.40 (d, *J* = 9.5 Hz); 131.87, 129.22, 127.97, 115.71, 114.01 (d, *J* = 2.9 Hz); 108.44 (d, *J* = 22.7 Hz); 56.25, 52.29; HRMS (ES+): *m*/*z* calculated for C_18_H_16_FN_5_O_3_: 368.1159 [M − H]^−^; found 368.1145.

##### Compound **11g**

The ethyl-4-{4-amino-6-[(3-fluoro-4-methoxyphenyl)amino]-1,3,5-triazin-2-yl}benzoate (**11g**) yield was 86%: ^1^H-NMR (400 MHz, chloroform-*d*) δ 8.41 (d, *J* = 8.8 Hz, 2H), 8.14 (d, *J* = 8.8 Hz, 2H), 7.67 (dd, *J* = 13.1, 2.6 Hz, 1H), 7.15 (d, *J* = 8.6 Hz, 1H), 7.10 (s, 1H), 6.95 (t, *J* = 9.0 Hz, 1H), 5.33 (s, 2H), 4.41 (q, *J* = 7.1 Hz, 2H), 3.90 (s, 3H), 1.42 (t, *J* = 7.1 Hz, 3H); ^13^C-NMR (100 MHz, DMSO-*d*_6_) δ 169.31, 167.12, 165.43, 164.35, 150.95 (d, *J* = 241.2 Hz); 142.16 (d, *J* = 10.9 Hz); 141.07, 133.42 (d, *J* = 9.4 Hz); 132.16, 129.18, 127.96, 115.73, 114.00 (d, *J* = 2.9 Hz); 108.44 (d, *J* = 22.5 Hz); 60.98, 56.25, 14.17; HRMS (ES+): *m*/*z* calculated for C_19_H_18_FN_5_O_3_: 384.1466 [M + H]^+^; found 384.1468.

##### Compound **11h**

The 4-{4-amino-6-[(3-fluoro-4-methoxyphenyl)amino]-1,3,5-triazin-2-yl}-2,6-difluorophenol (**11h**) yield was 68%: ^1^H-NMR (400 MHz, DMSO-*d*_6_) δ 10.91 (s, 1H), 9.52 (s, 1H), 8.38–7.68 (m, 3H), 7.42 (d, *J* = 8.2 Hz, 1H), 7.29–6.99 (m, 3H), 3.81 (s, 3H); ^13^C-NMR (100 MHz, DMSO-*d*_6_) δ 168.14, 166.98, 164.23, 151.91 (dd, *J* = 241.2, 6.9 Hz); 150.93 (d, *J* = 240.9 Hz); 142.13 (d, *J* = 10.9 Hz); 136.94 (t, *J* = 15.4 Hz); 133.42 (d, *J* = 9.4 Hz); 127.19 (t, *J* = 7.7 Hz); 115.70, 114.01 (d, *J* = 2.9 Hz); 111.01 (dd, *J* = 14.9, 7.6 Hz); 108.48 (d, *J* = 19.6 Hz); 56.25; HRMS (ES+): *m*/*z* calculated for C_16_H_12_F_3_N_5_O_2_: 362.0865 [[M − H]^−^; found 362.0877.

##### Compound **11i**

The 6-(4-fluoro-3-nitrophenyl)-*N*^2^-(3-fluoro-4-methoxyphenyl)-1,3,5-triazine-2,4-diamine (**11i**) yield was 66%: ^1^H-NMR (400 MHz, DMSO-*d*_6_) δ 9.72 (s, 1H), 9.01 (dd, *J* = 7.6, 2.0 Hz, 1H), 8.74–8.52 (m, 1H), 7.91 (s, 1H), 7.76 (dd, *J* = 11.2, 8.7 Hz, 1H), 7.47–7.31 (m, 3H), 7.11 (t, *J* = 9.4 Hz, 1H), 3.81 (s, 3H); ^13^C-NMR (100 MHz, DMSO-*d*_6_) δ 167.38, 167.04, 164.22, 156.59 (d, *J* = 266.3 Hz); 150.94 (d, *J* = 241.2 Hz); 142.30 (d, *J* = 11.3 Hz); 136.79 (d, *J* = 7.6 Hz); 135.10 (d, *J* = 9.8 Hz); 133.83 (d, *J* = 3.6 Hz); 133.21 (d, *J* = 10.2 Hz); 125.47, 118.96 (d, *J* = 21.4 Hz); 115.82, 113.98 (d, *J* = 2.9 Hz); 108.60 (d, *J* = 20.3 Hz); 56.26; HRMS (ES+): *m*/*z* calculated for C_16_H_12_F_2_N_6_O_3_: 373.0861 [M − H]^−^; found 373.0866.

### 3.2. Biological Evaluation

#### 3.2.1. Binding Assay at Human Adenosine Receptors

##### Binding Assay at hA_1_, hA_2A_, and hA_3_ AR

hA_1,_ hA_2A,_ and hA_3_ AR competition binding experiments were carried out in a multiscreen GF/C 96-well plate (Millipore, Madrid, Spain) pretreated with binding buffer (Hepes 20 mM, NaCl 100 mM, MgCl_2_ 10 mM, 2 U/mL adenosine deaminase, pH = 7.4 for hA_1_ AR; Tris-HCl 50 mM, EDTA 1 mM, MgCl_2_ 10 mM, 2 U/mL adenosine deaminase, pH = 7.4 for hA_2A_ AR; and Tris-HCl 50 mM, EDTA 1 mM, MgCl_2_ 5 mM, 2 U/mL adenosine deaminase, pH = 7.4 for hA_3_ AR, respectively). In each well was incubated 5 µg of membranes from Euroscreen CHO-A_1_ cell line, 5µg of membranes from Hela-A_2A_ cell line, or 30µg of membranes from Hela-A_3_ cell line and prepared in laboratory: 1 nM [^3^H]-DPCPX (140 Ci/mmol, 1 mCi/Ml, Perkin Elmer NET974001MC) for hA_1_ AR; 1 nM [^3^H]-ZM241385 (50 Ci/mmol, 1 mCi/mL, ARC-ITISA 0884) for hA_2A_ AR; and 10 nM [^3^H]-NECA (29.6 Ci/mmol, 1 mCi/mL, Perkin Elmer NET811250UC) for hA_3_ AR, respectively; and the compounds studied according to standard protocol. Non-specific binding was determined in the presence of R-PIA 10 µM (Sigma P4532) for hA_1_ AR; of NECA 50 µM (Sigma E2387) for hA_2A_ AR; and of R-PIA 100 µM (Sigma P4532) for hA_3_ AR, respectively. The reaction mixture (Vt: 200 µL/well) was incubated at 25 °C for 60 min (hA_1_ AR), 30min (hA_2A_ AR), or 180min (hA_3_ AR); whereafter it was filtered and washed four times (hA_1_ and hA_2A_ AR) or six times (hA_3_ AR) with 250 μL wash buffer (Hepes 20 mM, NaCl 100 mM, MgCl_2_ 10 mM pH = 7.4, for hA_1_ AR; Tris-HCl 50 mM, EDTA 1 mM, MgCl_2_ 10 mM, pH = 7.4, for hA_2A_ AR; and Tris-HCl 50mM pH = 7.4, for hA_3_ AR, respectively) before being measured in a microplate beta scintillation counter (MicrobetaTrilux, PerkinElmer, Madrid, Spain).

##### Binding Assay at hA_2B_ AR

hA_2B_ AR competition binding experiments were carried out in a multiscreen GF/C 96-well plate. In each well was incubated 25 µg of membranes from Euroscreen HEK-A_2B_ cell line and prepared in laboratory, 25 nM [^3^H]-DPCPX (140 Ci/mmol, 1 mCi/mL, Perkin Elmer NET974001MC) and compounds studied according to standard protocol. Non-specific binding was determined in the presence of NECA 1000 µM (Sigma E2397). The reaction mixture (Vt: 250 µL/well) was incubated at 25°C for 30 min, 200 µL was transferred to GF/C 96-well plate (Millipore, Madrid, Spain), and pretreated with binding buffer (Tris-HCl 50 Mm, EDTA 1 mM, MgCl_2_ 5 mM, Bacitracin 100 µg/µL, adenosine deaminase 2 U/Ml, pH = 6.5). It was then filtered and washed four times with 250 μL wash buffer (Tris-HCl 50 mM, EDTA 1 mM, MgCl_2_ 5 mM, pH = 6.5), before being measured in a microplate beta scintillation counter (MicrobetaTrilux, PerkinElmer, Madrid, Spain).

#### 3.2.2. cAMP Accumulation Assay

##### Antagonist Mode at hA_1_ or hA_3_ AR

hA_1_ and hA_3_ AR functional experiments were carried out in CHO-A_1_ and CHO-A_3_#18 cell line, respectively. The day before the assay, the cells were seeded on the 96-well culture plate (Falcon 353072). The cells were washed with wash buffer (Nutrient Mixture F12 Ham’s (Sigma N6658) for hA_1_ AR; Dulbecco’s modified eagle’s medium nutrient mixture F-12 ham (Sigma D8062) for hA_3_ AR; 25 mM Hepes; pH = 7.4). Wash buffer was replaced by incubation buffer (Mixture F12 Ham’s (Sigma N6658) for hA_1_ AR; Dulbecco’s modified eagle’s medium nutrient mixture F-12 ham (Sigma D8062) for hA_3_ AR; 25 mM Hepes, 20 µM Rolipram (Sigma R6520); pH = 7.4). Test compounds and XAC (Sigma X103) or MRS1220 (Sigma M228) as reference compound for hA_1_ and hA_3_ AR, respectively, were added and incubated at 37 °C for 15 min. Afterward, 0.1 µM of 5′-(*N*-Ethilcarboxamido) adenosine (NECA) (Sigma E2387) was added and incubated at 37 °C for 10 min. FSK (Sigma F3917) was added and incubated at 37 °C for 5 min. After incubation, the amount of cAMP was determined using cAMP Biotrak Enzyme immunoassay (EIA) System Kit (GE Healthcare RPN225).

##### Agonist Mode at hA_1_ or hA_3_ AR

hA_1_ and hA_3_ AR functional experiments were carried out in CHO-A_1_ and CHO-A_3_#18 cell line, respectively. The day before the assay, the cells were seeded on the 96-well culture plate (Falcon 353072). The cells were washed with wash buffer (Mixture F12 Ham’s (Sigma N6658) for hA_1_ AR; Dulbecco’s modified eagle’s medium nutrient mixture F-12 ham (Sigma D8062) for hA_3_ AR; 25mM Hepes; pH = 7.4). Wash buffer was replaced by incubation buffer (Mixture F12 Ham’s (Sigma N6658) for hA_1_ AR; Dulbecco’s modified eagle’s medium nutrient mixture F-12 ham (Sigma D8062) for hA_3_ AR; 25 mM Hepes, 20 µM Rolipram (Sigma R6520); pH = 7.4). The cells were pre-incubated at 37 °C for 15 min. Then, test compounds and 5′-(*N*-Ethilcarboxamido) adenosine (NECA) as reference compound (Sigma E2387) were added and incubated at 37 °C for 10 min. FSK (Sigma F3917) was added and incubated at 37 °C for 5 min. After incubation, the amount of cAMP was determined using cAMP Biotrak Enzyme immunoassay (EIA) System Kit (GE Healthcare RPN225).

#### 3.2.3. Cell Culture

For our study, A549 and NCI-H1299 cells were purchased from the Korean Cell Line Bank (KCLB, Seoul, South Korea). CHO-A1, Hela-A2A, and HEK-293T-A2B (Euroscreen, Gosselies, Belgium) were also used in this study. The cell culture medium (Roswell Park Memorial Institute (RPMI) 1640, Thermo Fisher Scientific, Waltham, MA, USA) contained 10% fetal bovine serum (FBS) and 1% Antibiotic-Antimycotic (Thermo Fisher Scientific, Waltham, MA, USA) and was used in accordance with guidelines provided by KCLB. The cells were cultured at 37 °C in an incubator with 5% CO_2_. When the cell density reached 90%, subcultures were generated using a trypsin-EDTA solution.

#### 3.2.4. Cell Viability Assay

A549 and NCI-H1299 cells were seeded in 96-well plates at a density of 1 × 10^5^ cells per well for 24 h before being exposed to compounds at various concentrations. After 48 h of compound treatment, the CellTiter 96 AQueous One Solution Cell Proliferation Assay Kit (Promega, Madison, WI, USA) was used to conduct a cell viability assay. The cells were incubated with solution reagents for 2 h at 37 °C before being measured for absorbance at 490 nm using a Synergy HTX Multi-Mode microplate reader (BioTek Instruments, Inc., Winooski, VT, USA).

#### 3.2.5. Microscopy

A549 cells were seeded in 6-well plates at a density of 1 × 10^6^ cells per well for microscopy analysis and then treated with compounds (20 and 40 μM). Cell morphology was examined after 48 h and images of the cells were acquired using an EVOS M5000 Imaging System (Thermo Fisher Scientific Inc., Waltham, MA, USA).

#### 3.2.6. Mitochondrial Membrane Potential (MMP) and Reactive Oxygen Species (ROS) Assay

A549 cells were incubated with a fluorescent indicator, specifically 100 nM tetramethylrhodamine methyl ester perchlorate (TMRM, Thermo Fisher Scientific, Waltham, MA, USA), to determine the mitochondrial membrane potential. The A549 cells treated with the compound were harvested after 48 h, washed in PBS, and re-suspended in FACS buffer (PBS supplemented with 2 percent fetal bovine serum). A flow cytometer (BD FACSVerse, BD Biosciences, San Jose, CA, USA) and the FlowJo software (FlowJo LLC, Ashland, OR, USA) were used to analyze the cells. For the reactive oxygen species measurements, we began by seeding A549 cells in 6-well plates up to a density of 1 × 10^6^ cells in each well, then we treated the cells with the compound (20 and 40 μM) for 48 h. 2′,7′-dichlorodihydrofluorescein diacetate acetyl ester (DCFDA) or MitoSOX-red mitochondrial superoxide indicator (Thermo Fisher Scientific, Waltham, MA, USA) was used to detect intracellular and mitochondrial ROS levels. DCFDA (1 μM) or MitoSOX (5 µM) was added to the cells and then incubated at room temperature for 20 min. PBS was then used to wash the cells, and the cells were subsequently re-suspended in FACS buffer (PBS supplemented with 1% FBS). Intracellular fluorescence measurements involved the use of a flow cytometer (BD FACSVerse, BD Biosciences, San Jose, CA, USA) and the FlowJo software (Version 10, TreeStar, Ashland, OR, USA).

#### 3.2.7. Live–Dead Assay

The A549 cells were seeded in 6-well plates at a density of 1 × 10^6^ cells per well, and the cells underwent treatment with the compound (20 and 40 μM) for 48 h. The A549 cells were analyzed using fluorescent dyes for both living and dead cells with the LIVE/DEAD kit (Thermo Fisher Scientific, Waltham, MA, USA). For cell staining, we used EthD-1 and calcein by referring to the manufacturer’s instructions. Images were captured with an EVOS M5000 Imaging System (Thermo Fisher Scientific Inc., Waltham, MA, USA).

#### 3.2.8. Statistical Analysis

GraphPad Prism (GraphPad Software, Inc., version 7, San Diego, CA, USA) was used for statistical analysis, and the results were presented as means ± SEM. The Student’s *t*-test was used to further analyze the data. The resulting *p*-values were considered statistically significant (* *p* < 0.05, ** *p* < 0.01, *** *p* < 0.001, **** *p* ≤ 0.0001).

### 3.3. Molecular Modeling

Schrödinger Maestro, version 13.1 (Release 2021-2, Schrödinger, LLC, New York, NY, USA) was used to perform the molecular docking of 1,3,5-triazine derivatives [32]. The structure of 1,3,5-triazine derivatives were drawn using Chemdraw [33] and its 3D conformation was generated using the Schrödinger LigPrep programme [34]. LigPrep generated all possible tautomers and states at pH 7.0 using Epik [35,36] for 1,3,5-triazine derivatives. The crystal structure of hA_1_ AR co-crystallized with PSB36 (PDB ID: 5N2S) was acquired from the Protein Data Bank (PDB). The homology model of hA_3_ AR was obtained from the research work by Lee et al. [27]. The protein was prepared using the Protein Preparation Wizard [37] to assign bond orders, add hydrogens at pH 7.0, and remove water molecules. Prime was used to complete missing side chains and loops. Finally, a restrained minimization was performed using the default constraint of 0.30 Å RMSD and the OPLS 2005 force field in order to complete the protein preparation. Molecular docking simulations were performed using the Glide ligand docking module for hA_1_ AR and the Glide induced fit docking module [38,39,40] for hA_3_ AR in standard protocol (standard precision) mode. The binding conformations of 1,3,5-triazine derivatives were analyzed in order to identify the important interactions with the active site residues of hA_1_ and hA_3_ AR.

## 4. Conclusions

We synthesized 1,3,5-triazine derivatives from cyanuric chloride and evaluated their binding affinity toward hARs. Of these derivatives, **11b** showed good binding affinity to both hA_1_ and hA_3_ AR (*K*_i_ =98.3 and 56.6 nM, respectively; selectivity index = 1.74). **11b** was found to be a hA_1_ and hA_3_ AR dual antagonist in cAMP accumulation assays at hA_1_ and hA_3_ AR. Compound **9c** showed the highest binding affinity to hA_1_ AR (*K*_i_ = 57.9 nM), and we demonstrated that **9c** exhibits cytotoxic activity in lung cancer cells. According to our findings, **9c** increased the expression of ROS, and this accumulation led to mitochondrial membrane dysfunction, which caused cell death. Thus, **9c** is a promising therapeutic agent for lung cancer, and further research efforts should focus on elucidating the mechanisms involved in detail.The binding modes of triazine derivatives were identified through a molecular docking study at hA_1_ and hA_3_ AR. The 1,3,5-triazine derivatives were predicted to bind to both hA_1_ and hA_3_ AR. We demonstrated that 1,3,5-triazine derivatives have the potential to be developed as hA_1_ and hA_3_ AR antagonists. As a result, further SAR efforts on the 1,3,5-triazine derivatives are already underway to improve their efficacy and selectivity to hA_1_ or hA_3_ AR, or to both hA_1_ and hA_3_ AR as dual ligands.

## Figures and Tables

**Table 1:** Percentage inhibition of 2-(*p*-phenol)-4-amino-1,3,5-triazine derivatives **9a–g** at hA_1_, hA_2A_, hA_2B_, and hA_3_ ARs.

** 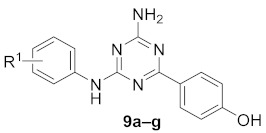 **					
**Compound**	**R^1^**	**Percentage Inhibition (%) at 10 μM ^a^**			
		**hA_1_ AR**	**hA_2A_ AR**	**hA_2B_ AR**	**hA_3_ AR**
**9a**	3-F-4-OCH_3_	99 ± 1	58 ± 1	72 ± 2	97 ± 1
**9b**	3,5-(OCH_3_)_2_	99 ± 1	84 ± 4	62 ± 2	93 ± 1
**9c**	3-OCH_3_-4-Cl	99 ± 1	94 ± 3	90 ± 1	95 ± 1
**9d**	3-CF_3_	69 ± 2	40 ± 5	74 ± 1	95 ± 1
**9e**	3,5-(CH_3_)_2_	96 ± 3	58 ± 3	42 ± 2	92 ± 1
**9f**	4-*N*-piperidine	28 ± 1	28 ± 3	44 ± 4	38 ± 1
**9g**	2,4-(CH_3_)_2_	99 ± 1	80 ± 4	47 ± 2	91 ± 1

^a^ All binding experiments were performed using adherent mammalian cells stably transfected with cDNA encoding the appropriate hAR (hA_1_ and hA_3_ AR in CHO cells, hA_2A_ AR in Hela cells, and hA_2B_ AR in HEK-293 cells). Binding was carried out using 2 nM [^3^H]DPCPX, 3 nM [^3^H]ZM241385, 25 nM [^3^H]DPCPX, and 0.5 nM [^3^H]NECA as radioligands for hA_1_, hA_2A_, hA_2B_, and hA_3_ AR, respectively. Values are expressed as the percentage inhibition of specific radioligand binding at 10 μM, with nonspecific binding defined using 10 μM NECA. AR: adenosine receptor; cDNA: complementary DNA; CHO: Chinese hamster ovary; DPCPX: 8-cyclopentyl-1,3-dipropylxanthine; hAR: human adenosine receptor; HEK: human embryonic kidney; NECA: 5-*N*-ethylcarboxamido adenosine.

**Table 2:** Binding affinity to hA_1_ and hA_3_ ARs.

Compound	R^1^	*K*_i_(nM) ^a^		Selectivity (hA_1_ AR:hA_3_ AR)
		hA_1_ AR	hA_3_ AR	
**9a**	3-F-4-OCH_3_	139.3	55.5	2.51
**9b**	3,5-(OCH_3_)_2_	69.7	ND	-
**9c**	3-OCH_3_-4-Cl	57.9	661.1	0.0875
**9d**	3-CF_3_	ND	1258	-
**9e**	3,5-(CH_3_)_2_	872.1	ND	-
**9f**	4-*N*-piperidine	ND	ND	-
**9g**	2,4-(CH_3_)_2_	116.5	ND	-

^a^ All binding experiments were performed using adherent mammalian cells stably transfected with cDNA encoding the appropriate hAR (hA_1_ and hA_3_ in CHO cells). Binding was carried out using 2 nM [^3^H]DPCPX and 0.5 nM [^3^H]NECA as radioligands for hA_1_ and hA_3_ AR, respectively. AR: adenosine receptor; cDNA: complementary DNA; CHO: Chinese hamster ovary; DPCPX: 8-cyclopentyl-1,3-dipropylxanthine; hAR: human adenosine receptor; ND: not determined; NECA: 5-*N*-ethylcarboxamido adenosine.

**Table 3:** Percentage inhibition of 6-(3-fluoro-4-methoxyaniline)-4-amino-1,3,5-triazine derivatives **11a**–**i** at hA_1_, hA_2A_, hA_2B_, and hA_3_ ARs.

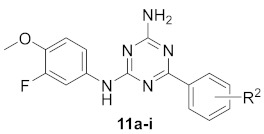					
**Compound**	**R^2^**	**Percentage Inhibition (%) at 10 μM ^a^**			
		**hA_1_ AR**	**hA_2A_ AR**	**hA_2B_ AR**	**hA_3_ AR**
**9a**	4-OH	99 ± 1	56 ± 3	66 ± 3	97 ± 1
**11a**	4-OCH_3_	95 ± 1	83 ± 1	65 ± 2	97 ± 1
**11b**	4-F	98 ± 1	49 ± 1	56 ± 1	95 ± 4
**11c**	4-OCF_3_	48 ± 4	81 ± 1	27 ± 6	60 ± 1
**11d**	4-CF_3_	74 ± 1	30 ± 1	93 ± 3	79 ± 1
**11e**	4-CN	45 ± 5	50 ± 2	52 ± 1	72 ± 3
**11f**	4-C(O)OCH_3_	52 ± 1	35 ± 6	55 ± 2	85 ± 1
**11g**	4-C(O)OCH_2_CH_3_	56 ± 1	48 ± 6	35 ± 2	78 ± 3
**11h**	3,5-diF-4-OH	88 ± 1	26 ± 5	66 ± 6	79 ± 1
**11i**	3-NO_2_-4-F	53 ± 1	57 ± 2	72 ± 6	83 ± 1

^a^ All binding experiments were performed using adherent mammalian cells stably transfected with cDNA encoding the appropriate hAR (hA_1_ and hA_3_ in CHO cells, hA_2A_ in Hela cells, and hA_2B_ in HEK-293 cells). Binding was carried out using 2 nM [^3^H]DPCPX, 3 nM [^3^H]ZM241385, 25 nM [^3^H]DPCPX, and 0.5 nM [^3^H]NECA as radioligands for hA_1_, hA_2A_, hA_2B_, and hA_3_ AR, respectively. Values are expressed as the percentage inhibition of a specific radioligand binding at 10 μM, with nonspecific binding defined using 10 μM NECA. AR: adenosine receptor; cDNA: complementary DNA; CHO: Chinese hamster ovary; DPCPX: 8-cyclopentyl-1,3-dipropylxanthine; hAR: human adenosine receptor; HEK: human embryonic kidney; NECA: 5-*N*-ethylcarboxamido adenosine.

**Table 4:** Binding affinity to hA_1_ and hA_3_ ARs.

Compound	R^2^	*K*_i_ (nM) ^a^		Selectivity (hA_1_ AR:hA_3_ AR)
		hA_1_ AR	hA_3_ AR	
**9a**	4-OH	139.3	55.5	2.51
**11a**	4-OCH_3_	78.1	13.3	5.87
**11b**	4-F	98.3	56.6	1.74

^a^ All binding experiments were performed using adherent mammalian cells stably transfected with cDNA encoding the appropriate hAR (hA_1_ and hA_3_ in CHO cells). Binding was carried out using 2 nM [^3^H]DPCPX and 0.5 nM [^3^H]NECA as radioligands for hA_1_ and hA_3_ AR, respectively. AR: adenosine receptor; cDNA: complementary DNA; CHO: Chinese hamster ovary; DPCPX: 8-cyclopentyl-1,3-dipropylxanthine; hAR: human adenosine receptor; NECA: 5-*N*-ethylcarboxamido adenosine.

## Data Availability

All data that generated or analyzed during this study are available from the corresponding authors upon justified request.

## Supplementary Materials

The following supporting information can be downloaded at: https://www.mdpi.com/article/10.3390/molecules27134016/s1, Figure S1: ^1^H and ^13^C NMR Copies of **9a**–**g** and **11a**–**i**; Figure S2. HR-MS Copies of **9a**–**g**, and **11a**–**i**; Figure S3: Molecular Docking of **9a**, **11b**, **11h** and **11i** in hA_1_ AR.

## Sample Availability

Samples of the compounds are available from the authors.
