# Supplementary material for: Design, Synthesis and Biological Evaluation of 1,3,5-Triazine Derivatives Targeting hA1 and hA3 Adenosine Receptor
Source: Molecules. 2022 Jun 22;27(13):4016. doi: 10.3390/molecules27134016 (PMC9268102; doi:10.3390/molecules27134016)

Supplementary Materials

Design, Synthesis, and Biological Evaluation of 1,3,5-Triazine Derivatives Targeting hA_1 and hA_3 Aenosine Receptor

Sujin Park [1,2], Yujin Ahn [3,4], Yongchan Kim [2], Eun Joo Roh [2], Yoonji Lee [5], Chaebin Han [1,2], Hee Min Yoo [3,4,*] and Jinha Yu [1,*]

[1] Department of Pharmacy, College of Pharmacy and Graduate School of Pharmaceutical Sciences, Ewha Womans University, Seoul 03760, Korea

[2] Biomedical Research Institute, Korea Institute of Science and Technology (KIST), Seoul 02792, Korea

[3] Biometrology Group, Korea Research Institute of Standards and Science (KRISS), Daejeon 34113, Korea

[4] Department of Precision Measurement, University of Science and Technology (UST), Daejeon 34113, Korea; [5]College of Pharmacy, Chung-Ang University, Seoul 06974, Korea

Figure S1. ^{1}H and ^{13}C NMR Copies of Compound **9a-g**, and **11a-i**
Figure S2. HR-MS Copies of Compound **9a-g**, and **11a-i**
Figure S3. Molecular Docking of Compound **9a**, **11a**, and **11b**

Figure S1. ^{1}H and ^{13}C NMR Copies of compound **9a-g** and **11a-i**

4-(4-Amino-6-((3-fluoro-4-methoxyphenyl)amino)-1,3,5-triazin-2-yl)phenol (**9a**)

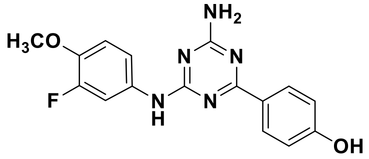

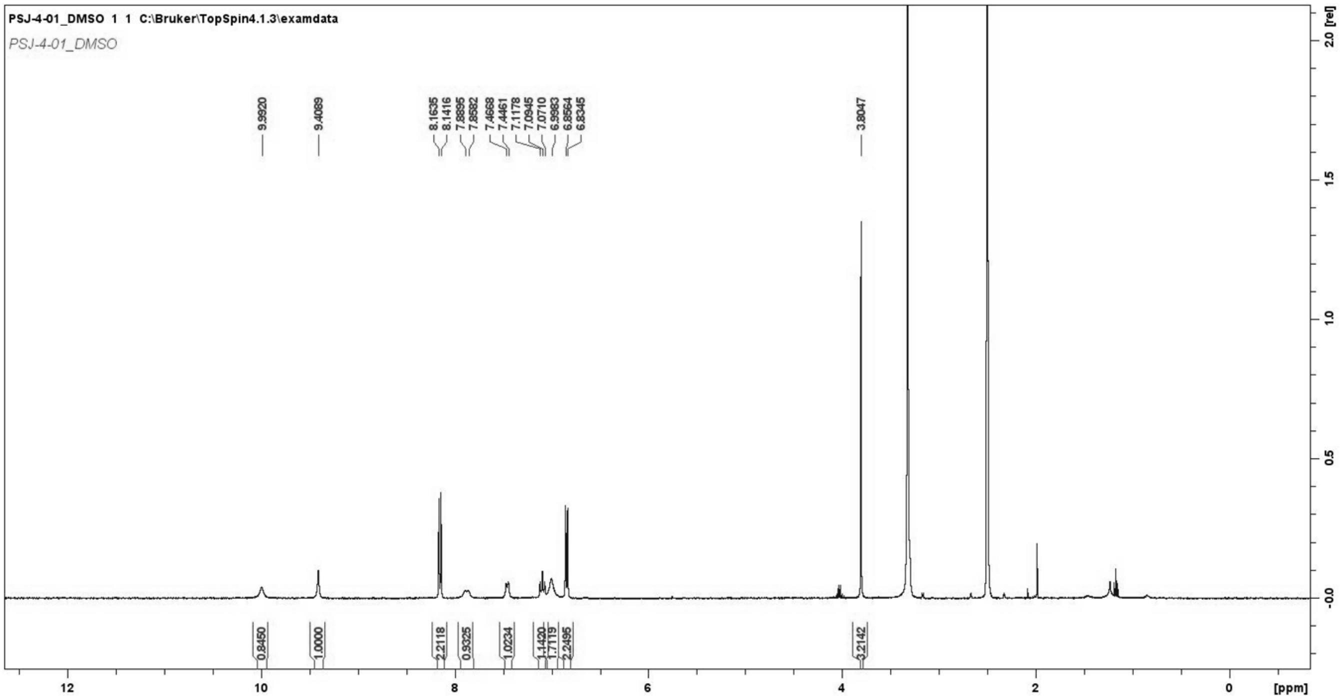

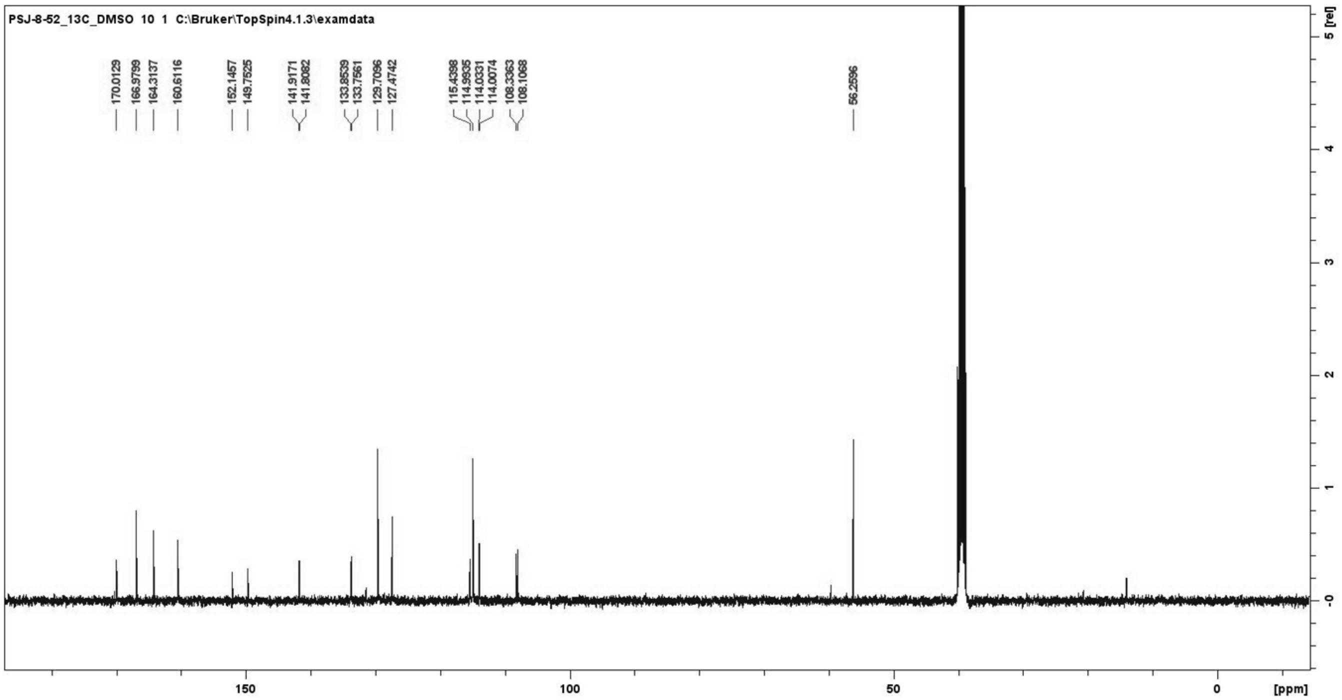

4-(4-Amino-6-((3,5-dimethoxyphenyl)amino)-1,3,5-triazin-2-yl)phenol (**9b**)

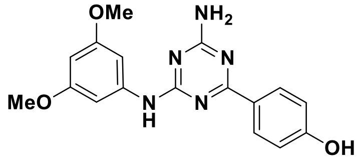

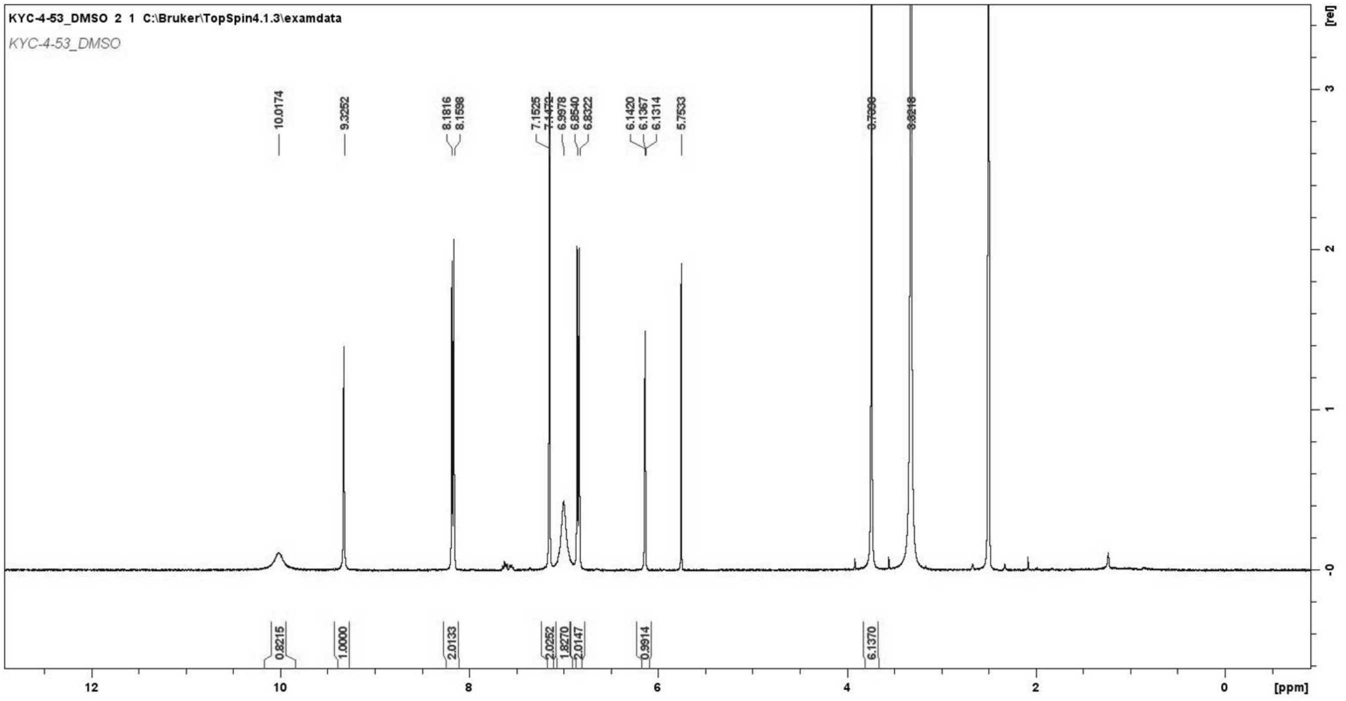

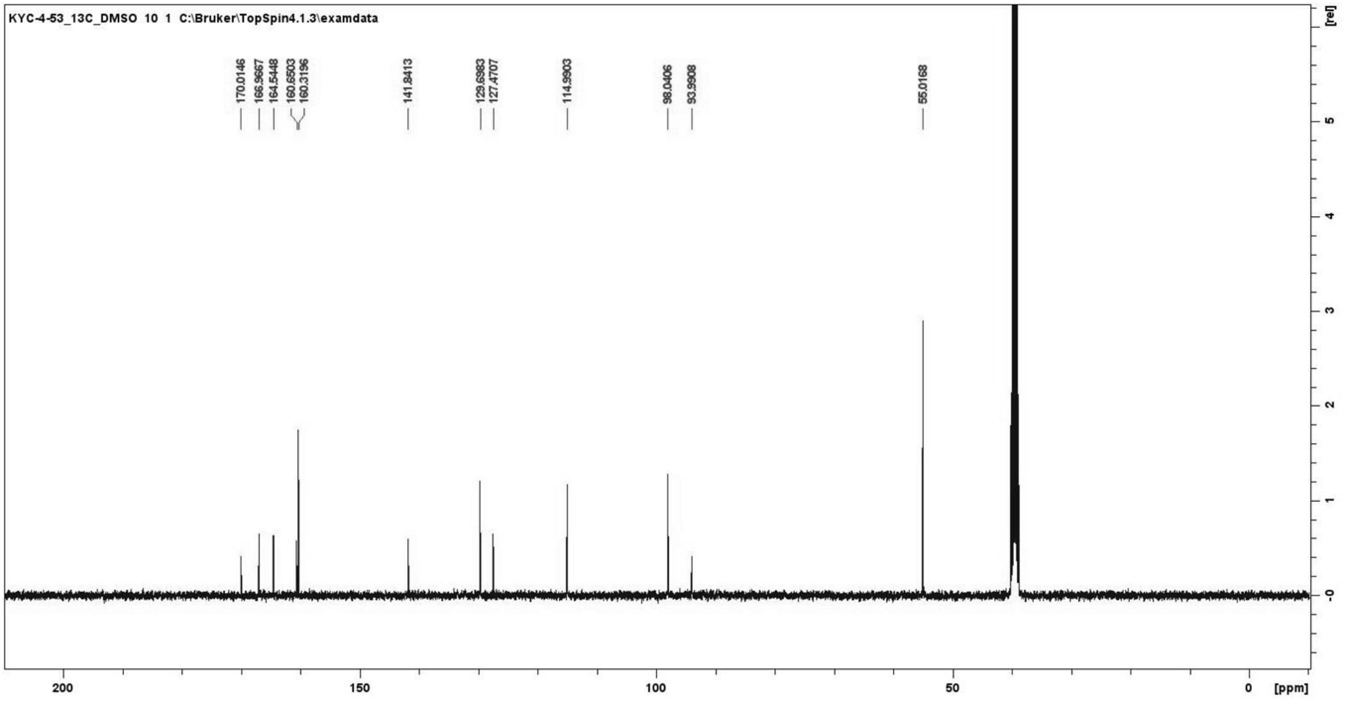

4-(4-Amino-6-((4-chloro-3-methoxyphenyl)amino)-1,3,5-triazin-2-yl)phenol (**9c**)

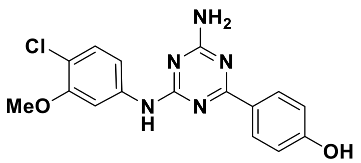

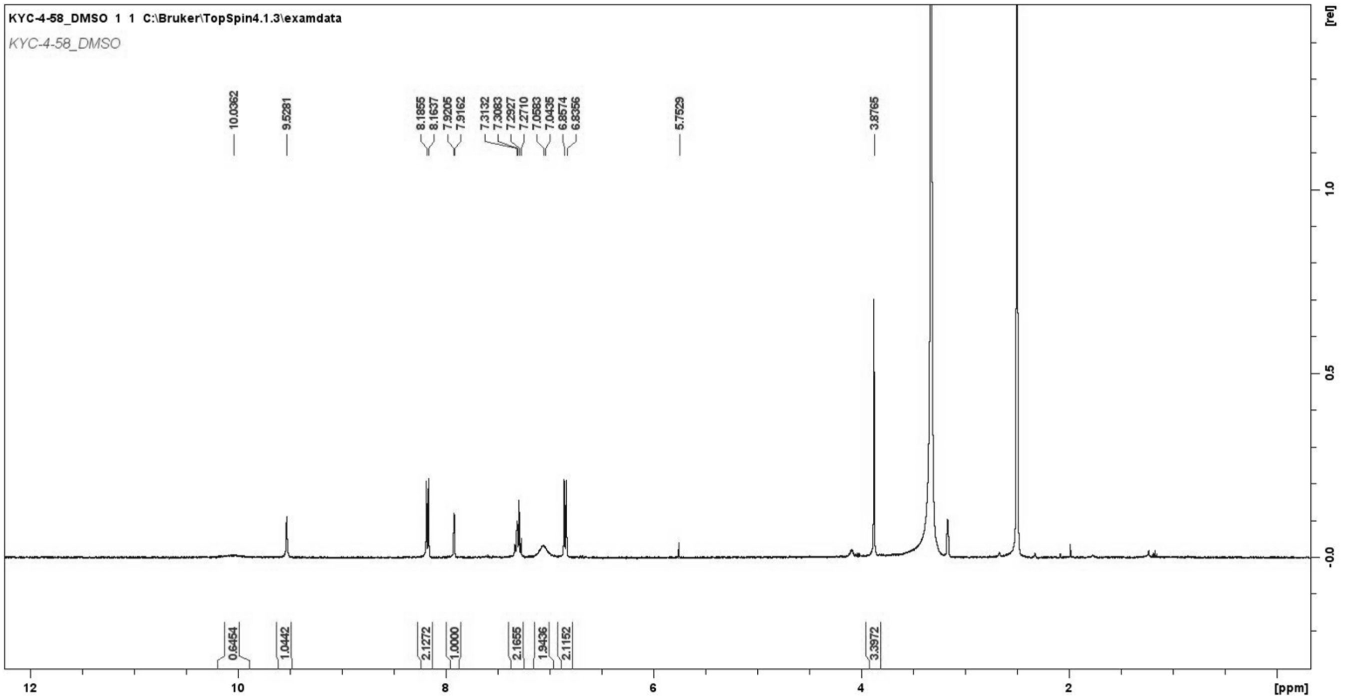

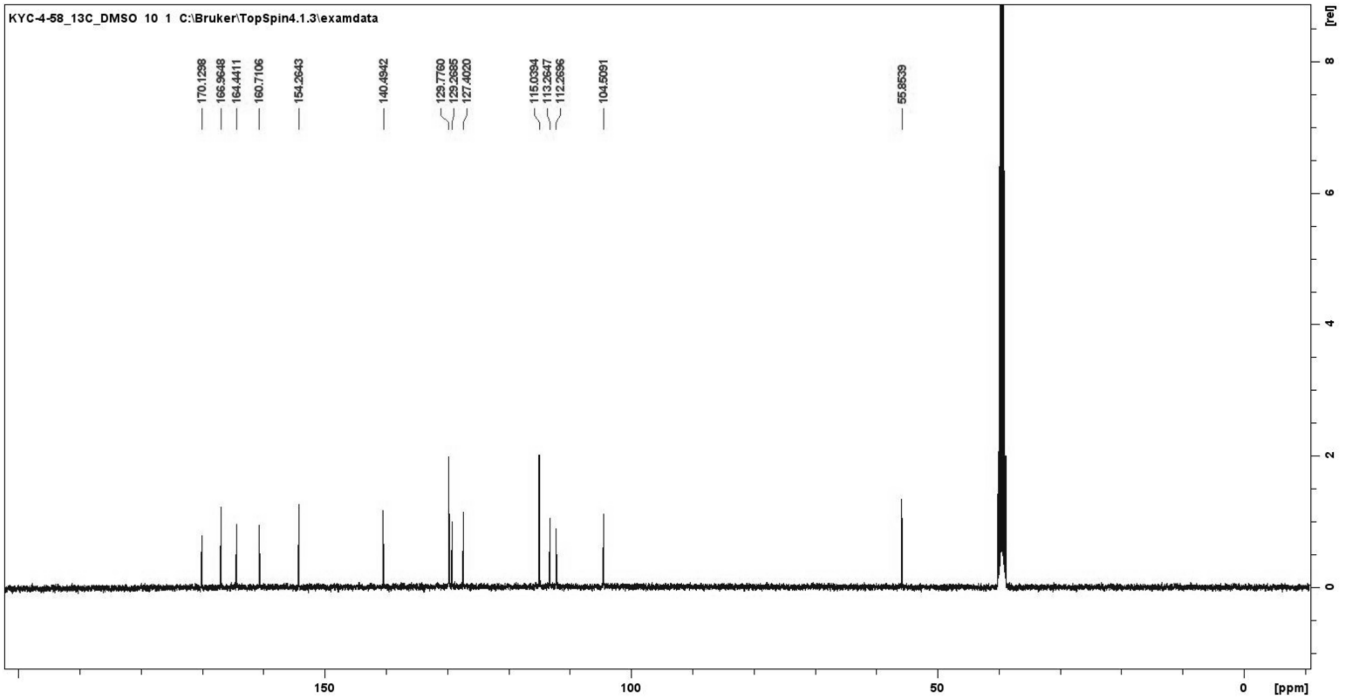

4-(4-Amino-6-((3-(trifluoromethyl)phenyl)amino)-1,3,5-triazin-2-yl)phenol (**9d**)

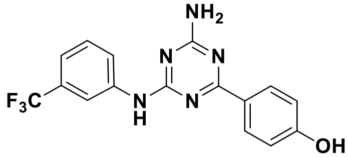

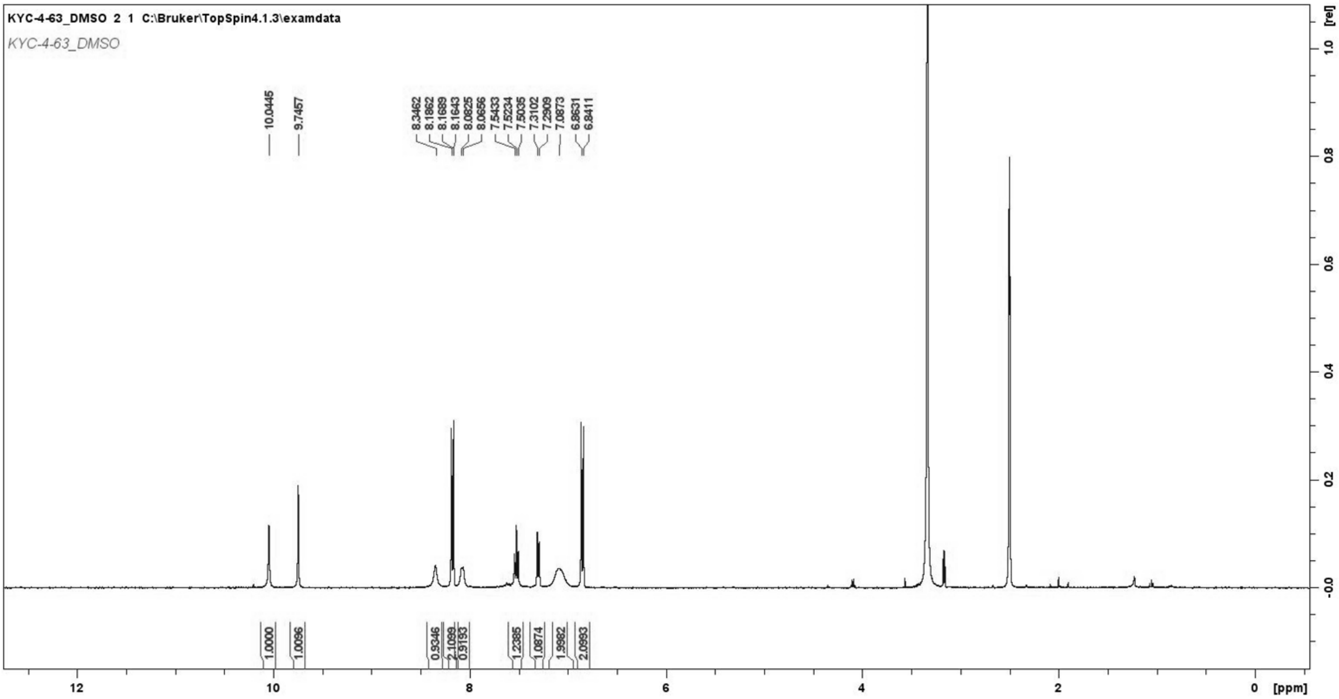

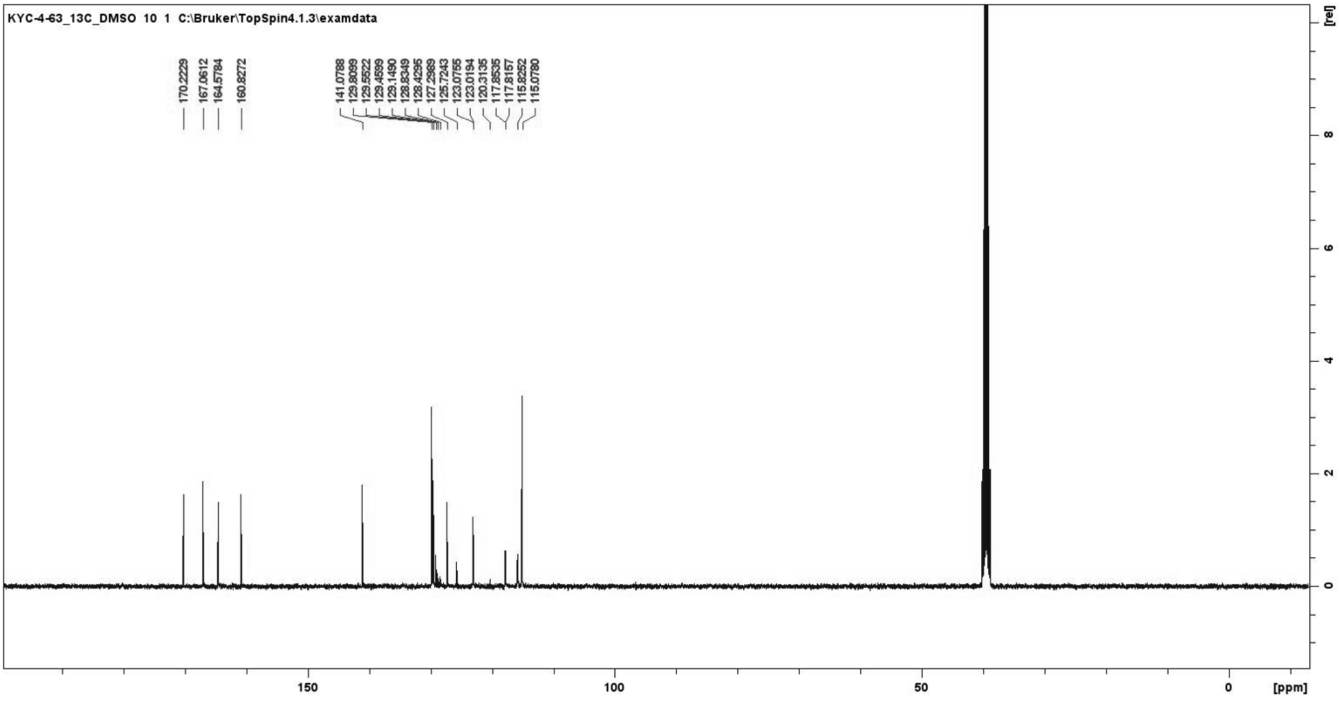

4-(4-Amino-6-((3,5-dimethylphenyl)amino)-1,3,5-triazin-2-yl)phenol (**9e**)

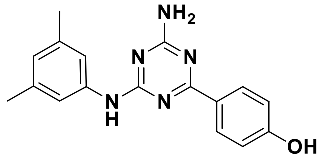

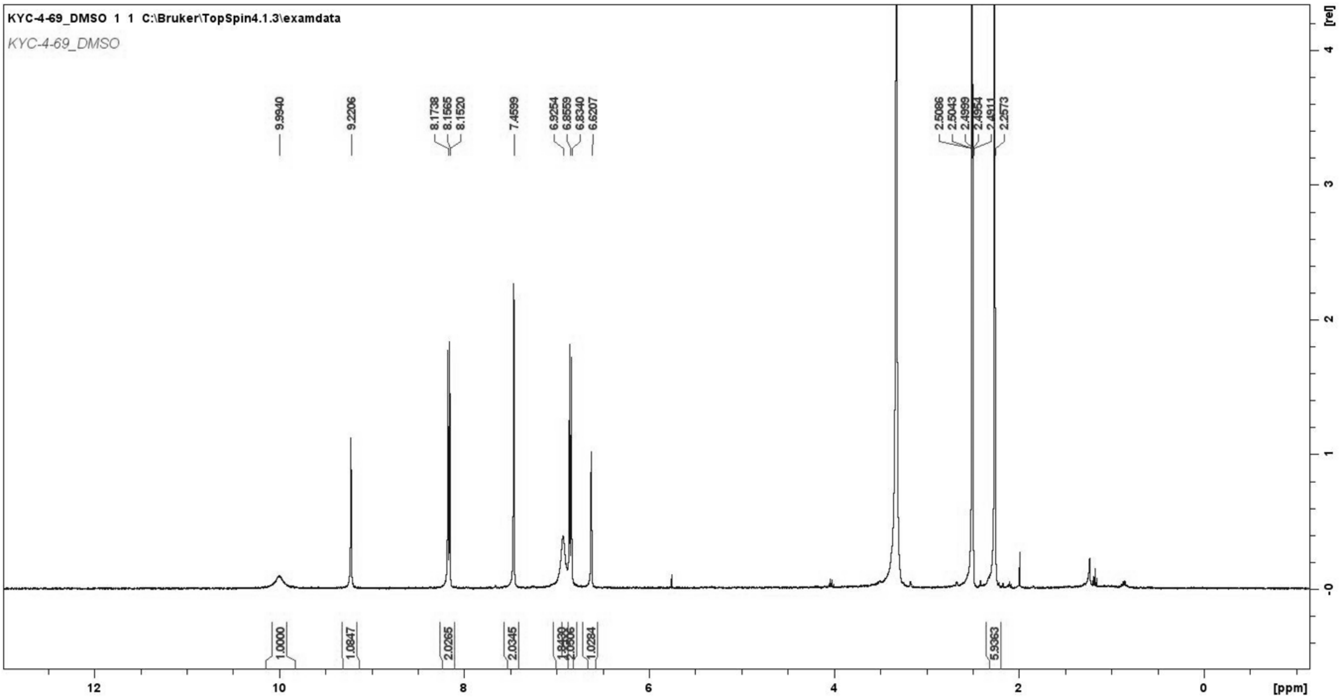

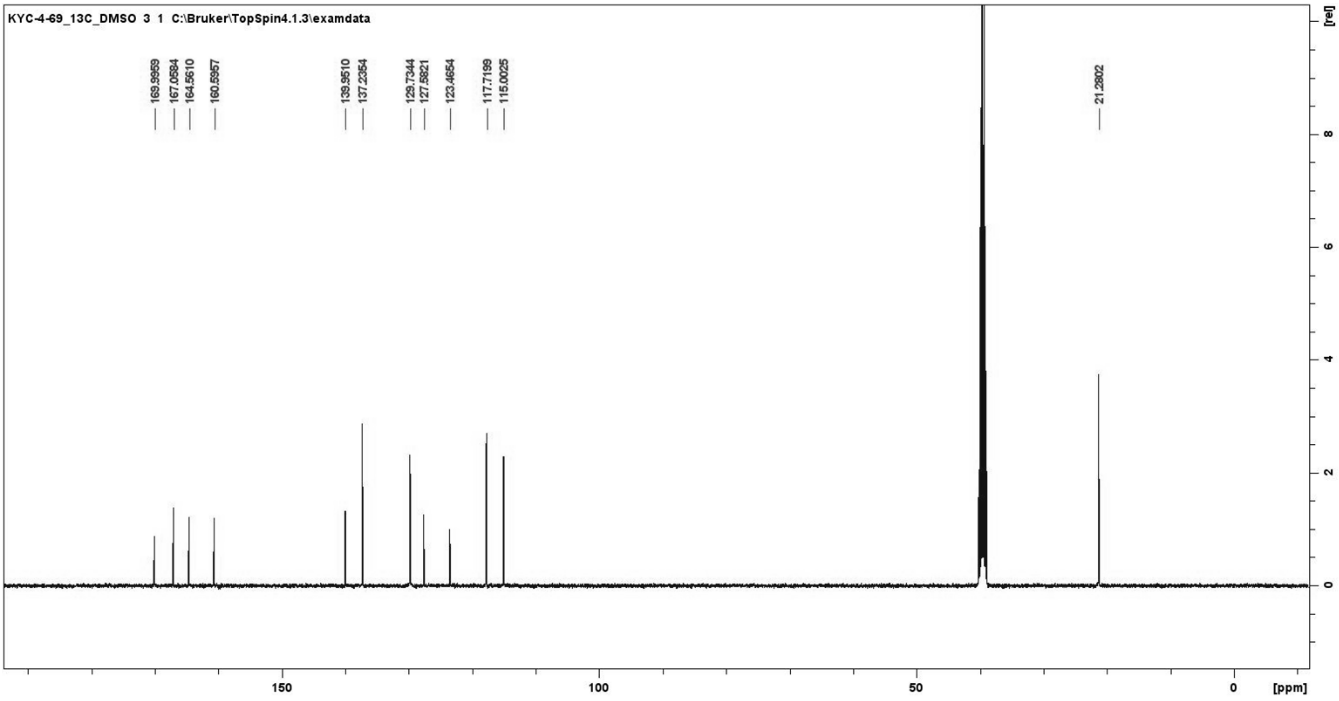

4-(4-Amino-6-((4-(piperidin-1-yl)phenyl)amino)-1,3,5-triazin-2-yl)phenol (**9f**)

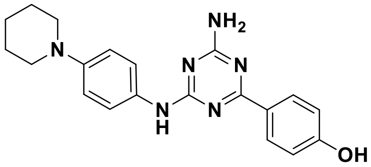

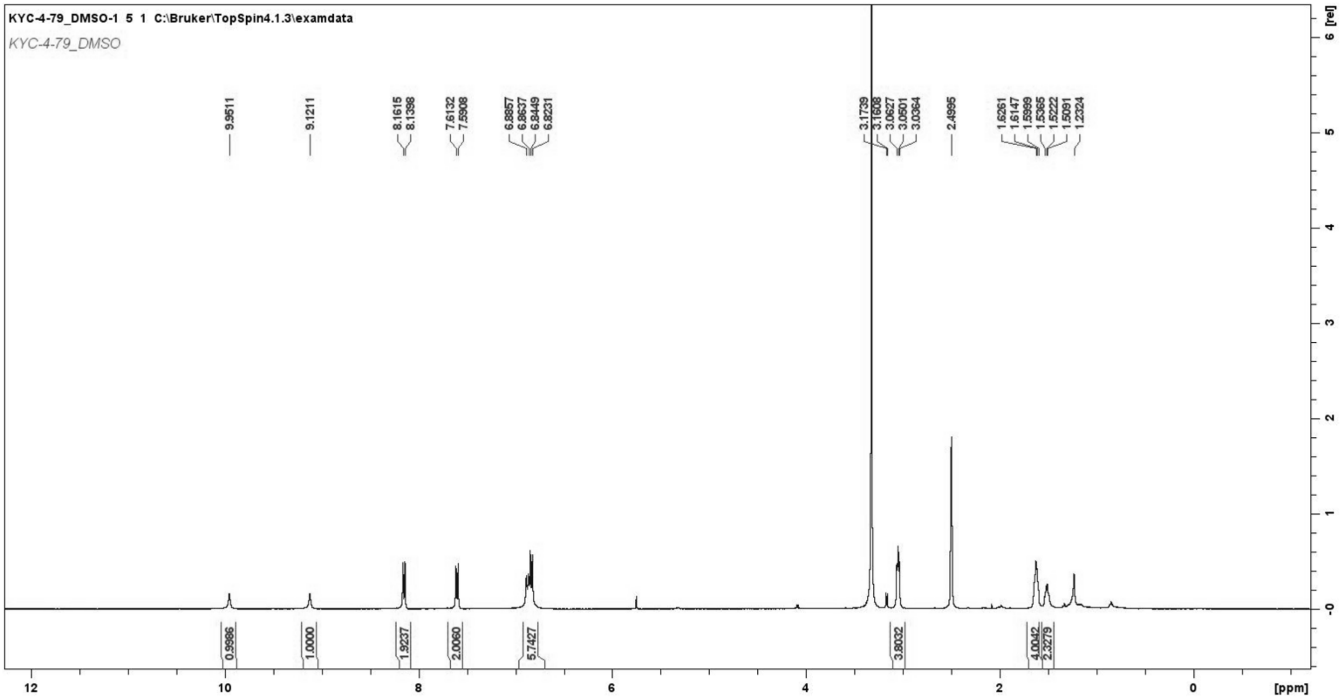

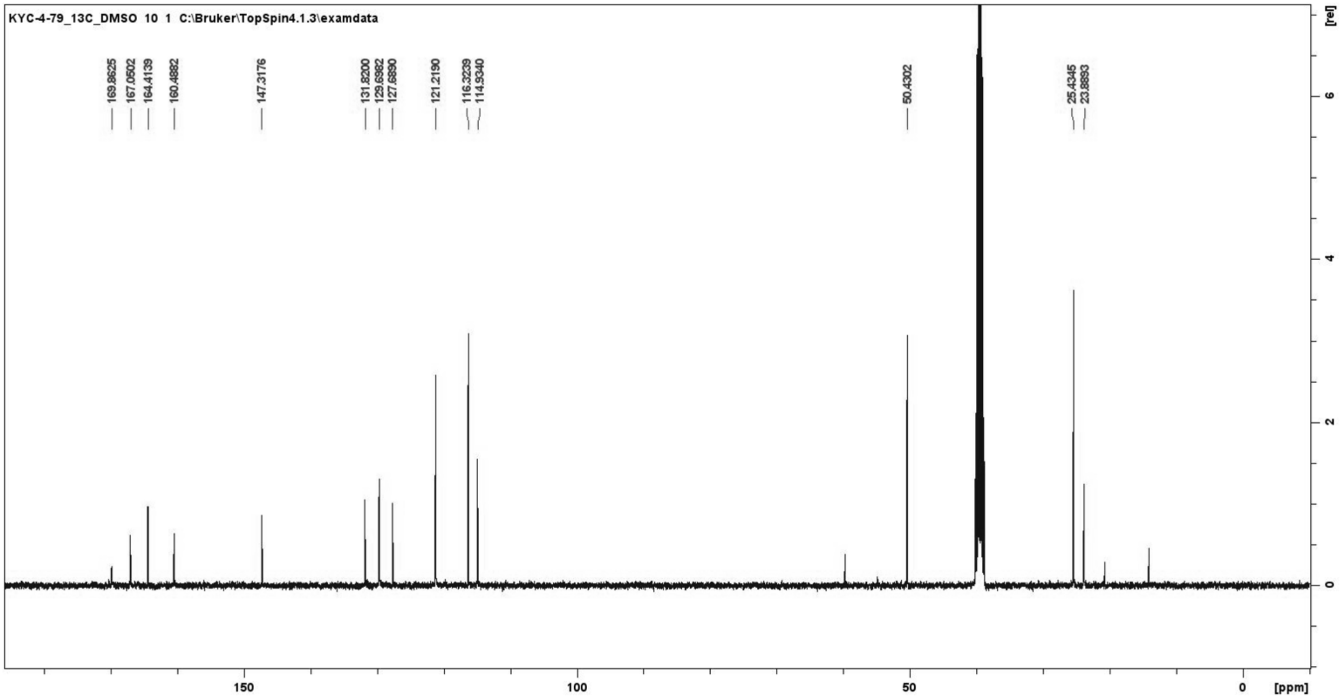

4-(4-Amino-6-((2,4-dimethylphenyl)amino)-1,3,5-triazin-2-yl)phenol (**9g**)

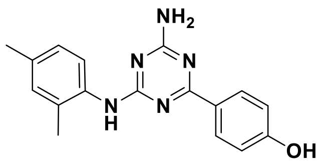

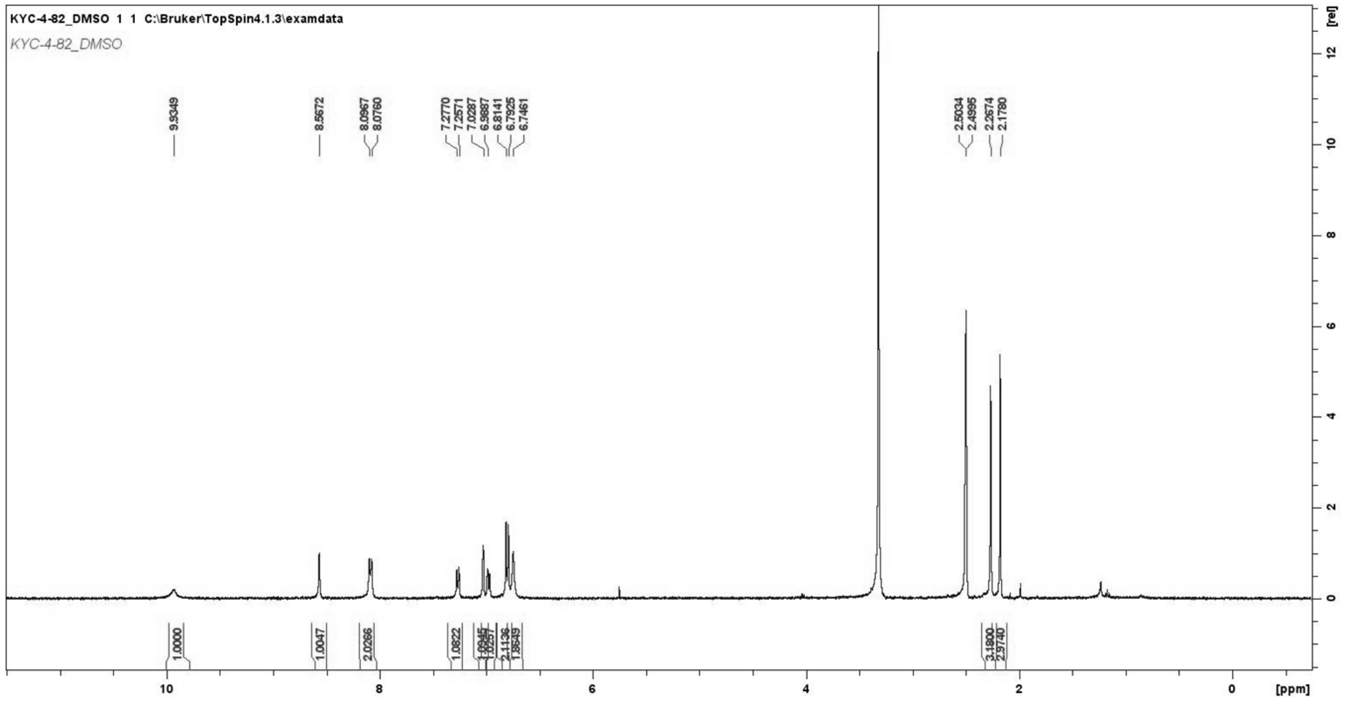

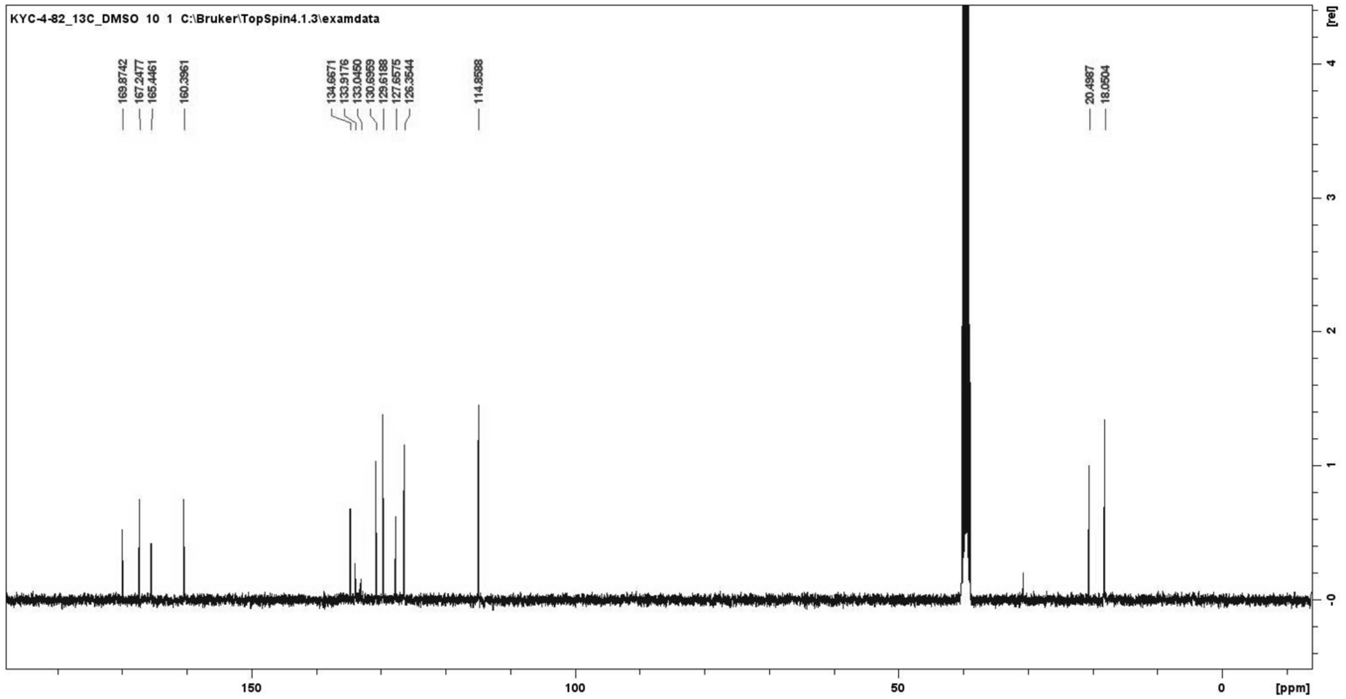

N[2]-(3-Fluoro-4-methoxyphenyl)-6-(4-methoxyphenyl)-1,3,5-triazine-2,4-diamine (**11a**)

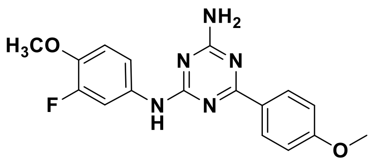

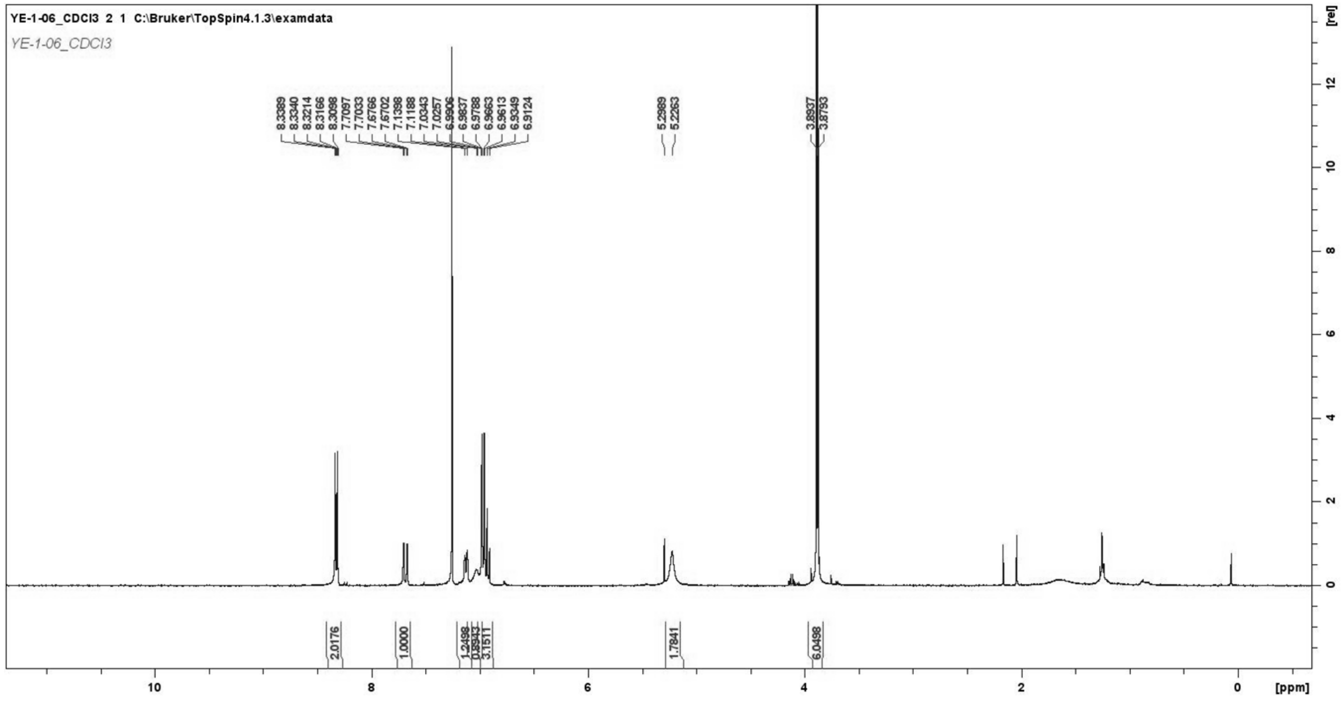

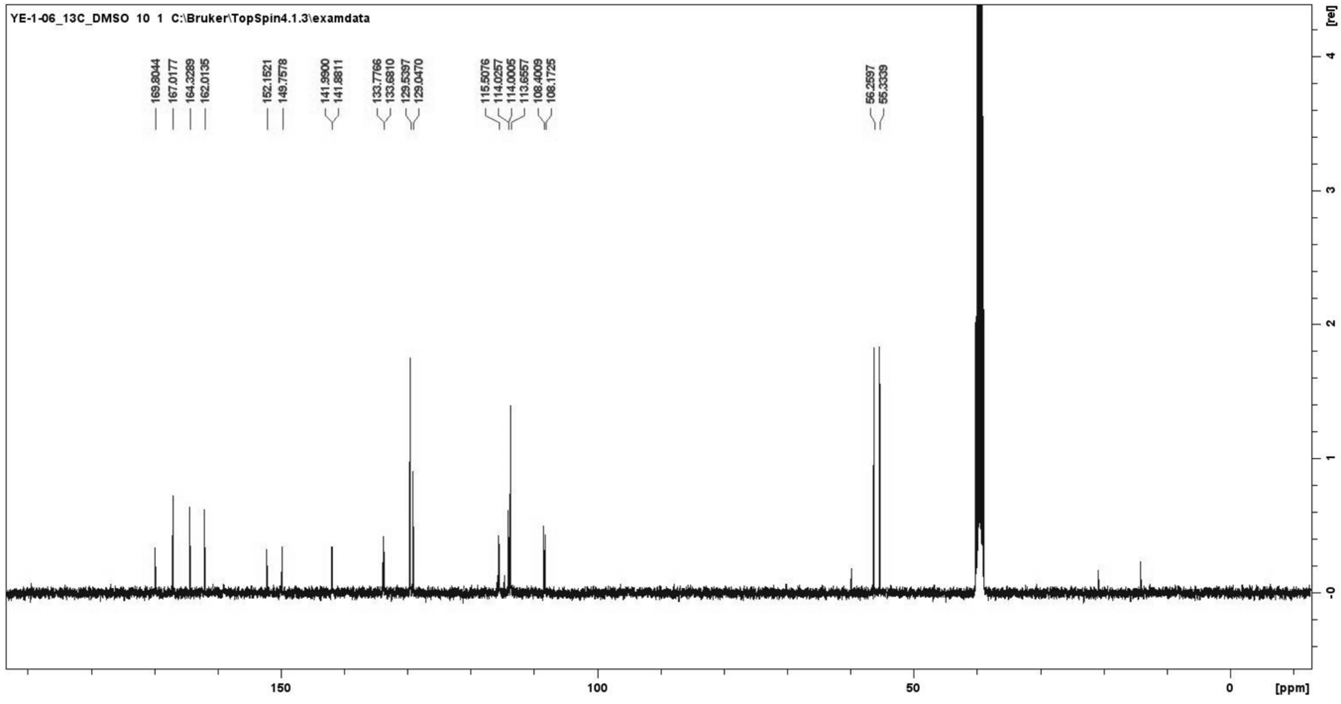

N^2-(3-Fluoro-4-methoxyphenyl)-6-(4-fluorophenyl)-1,3,5-triazine-2,4-diamine (**11b)**

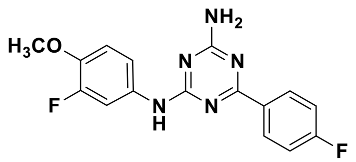

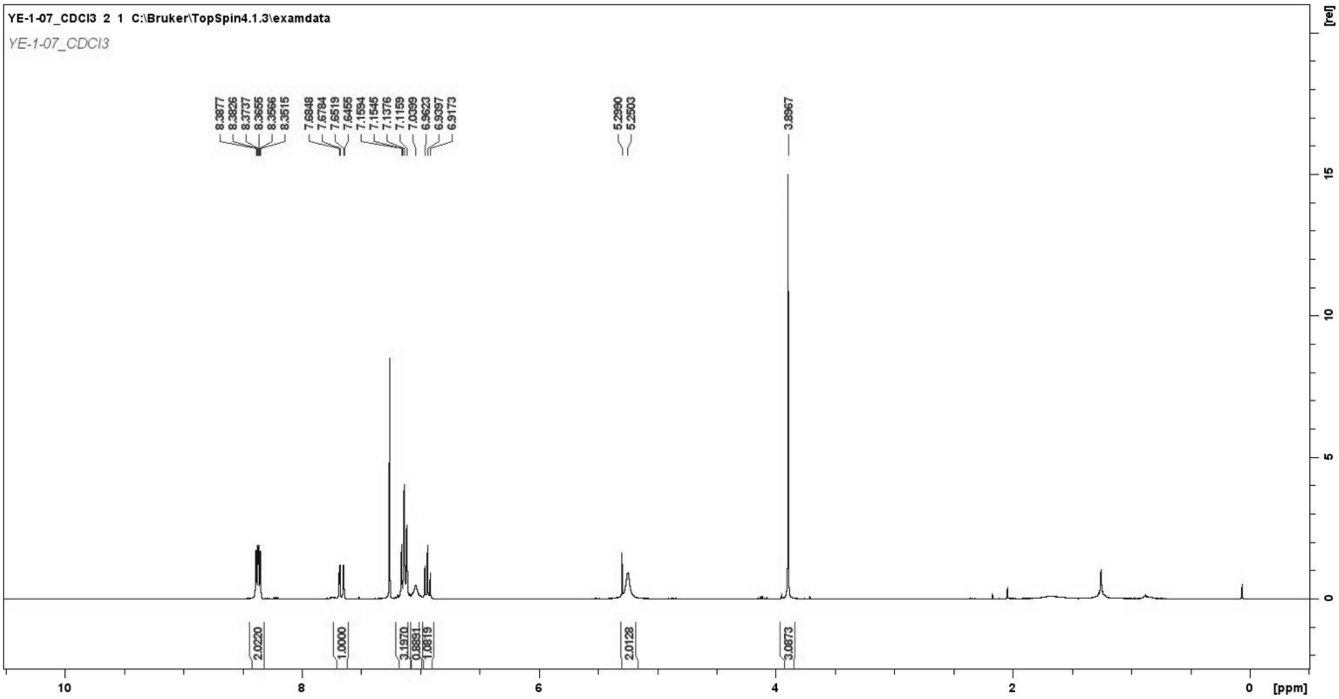

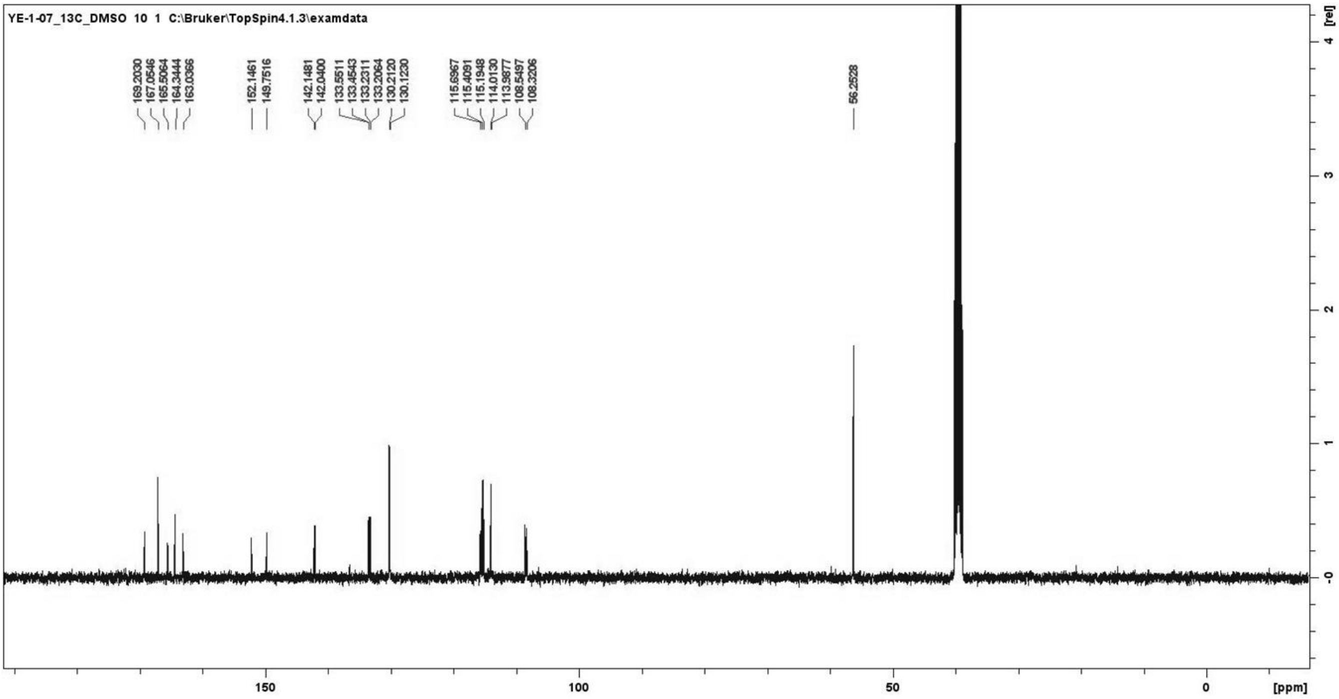

N[2]-(3-Fluoro-4-methoxyphenyl)-6-(4-(trifluoromethoxy)phenyl)-1,3,5-triazine-2,4-diamine (**11c**)

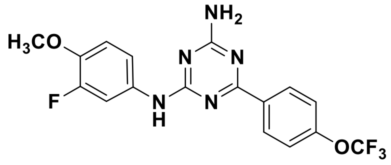

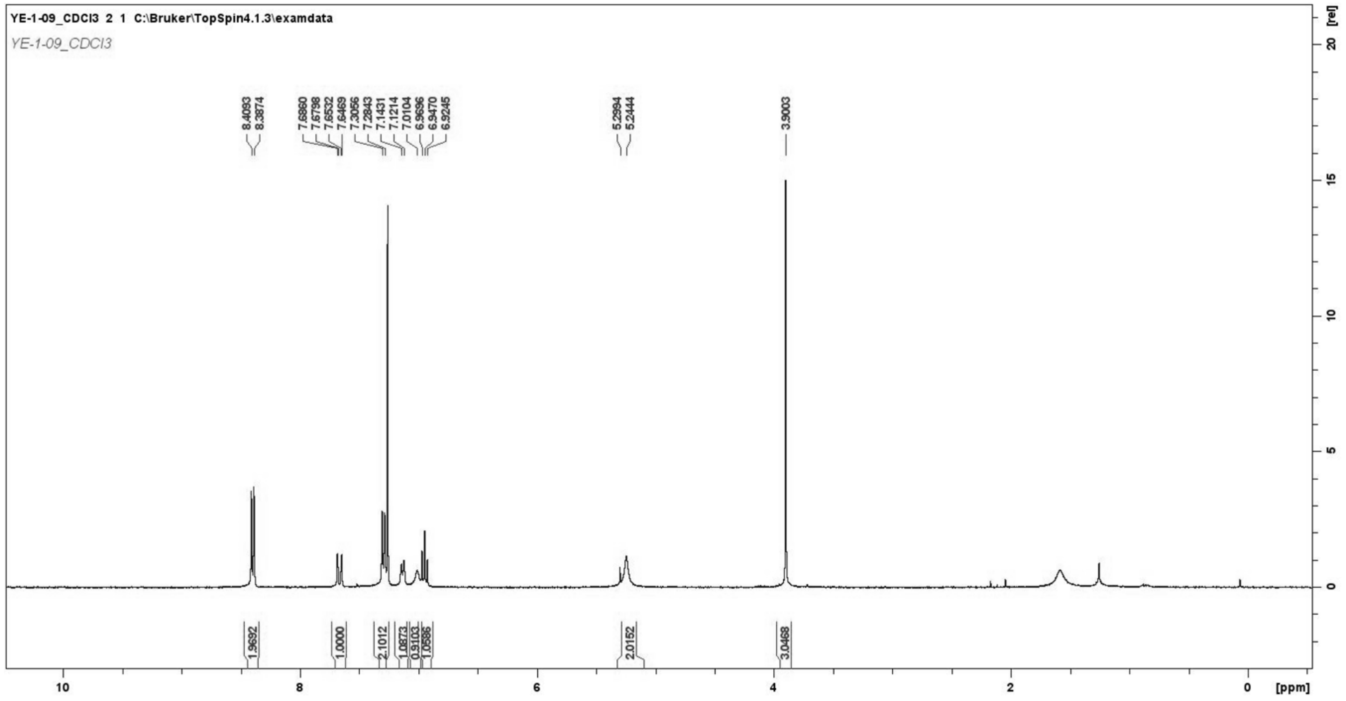

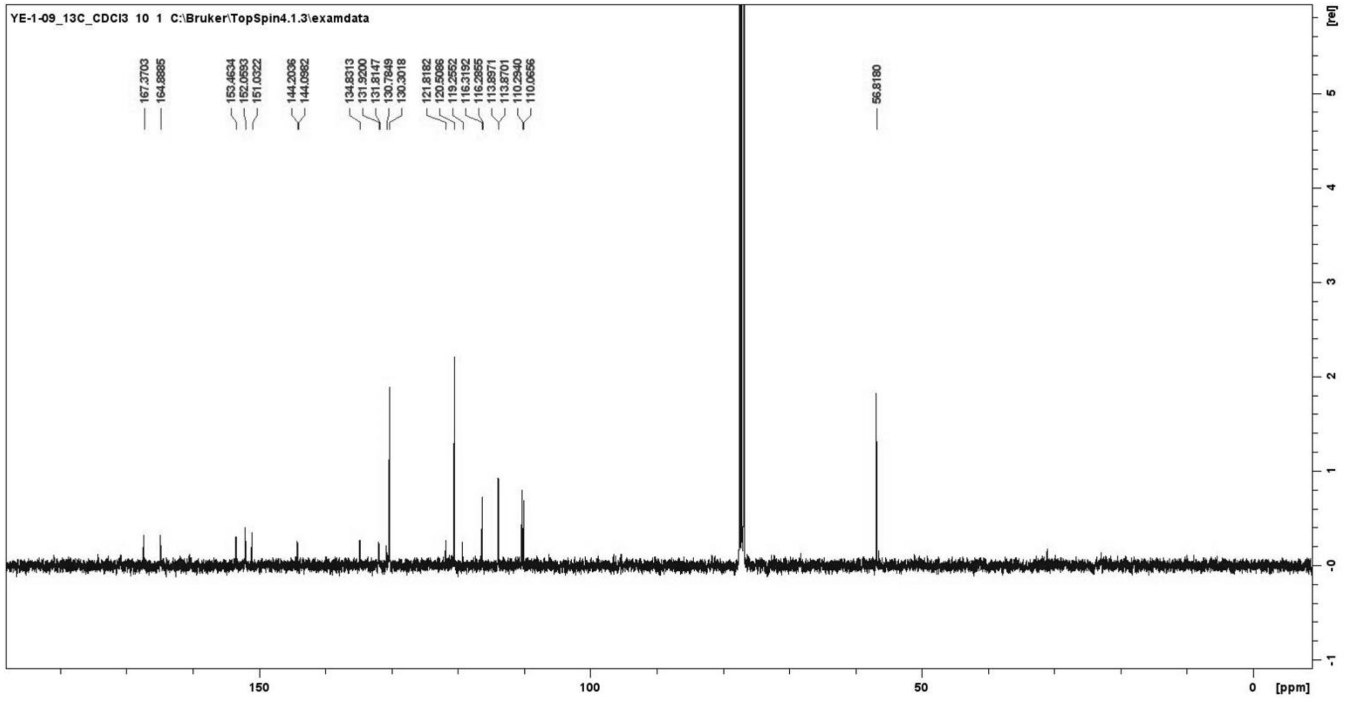

N[2]-(3-Fluoro-4-methoxyphenyl)-6-(4-(trifluoromethyl)phenyl)-1,3,5-triazine-2,4-diamine (**11d**)

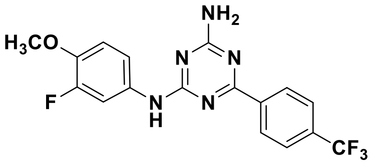

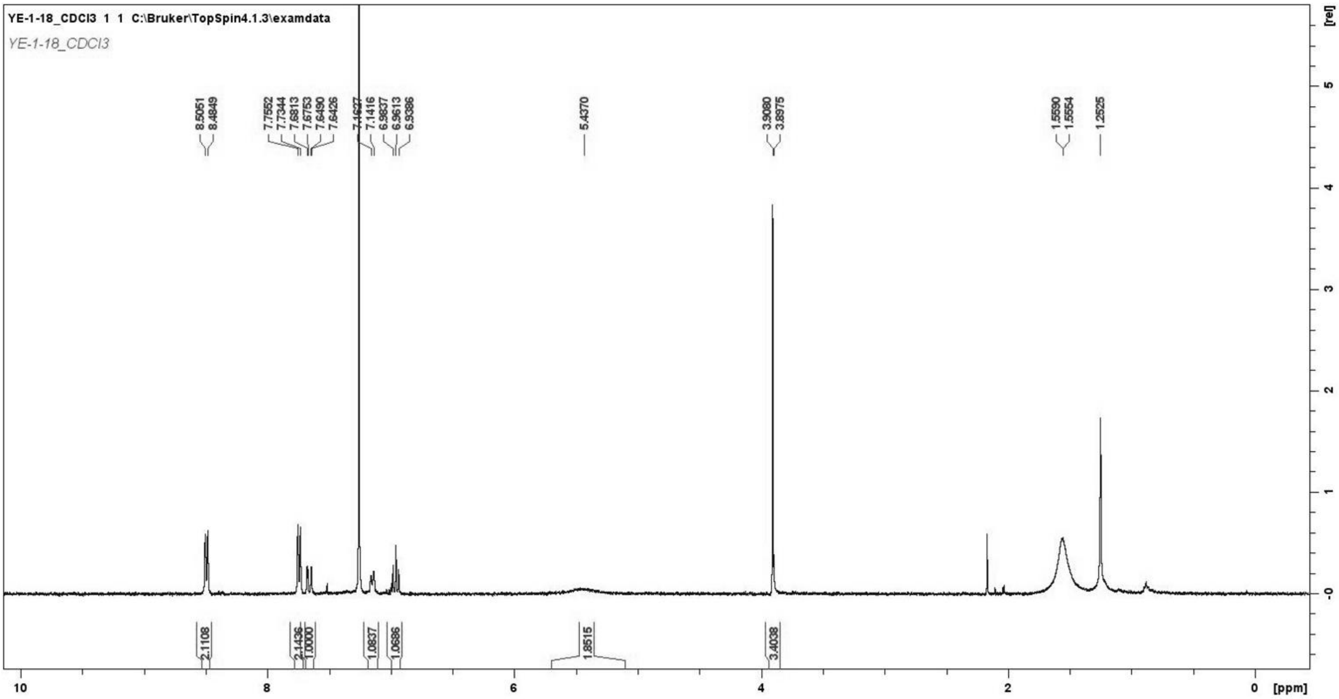

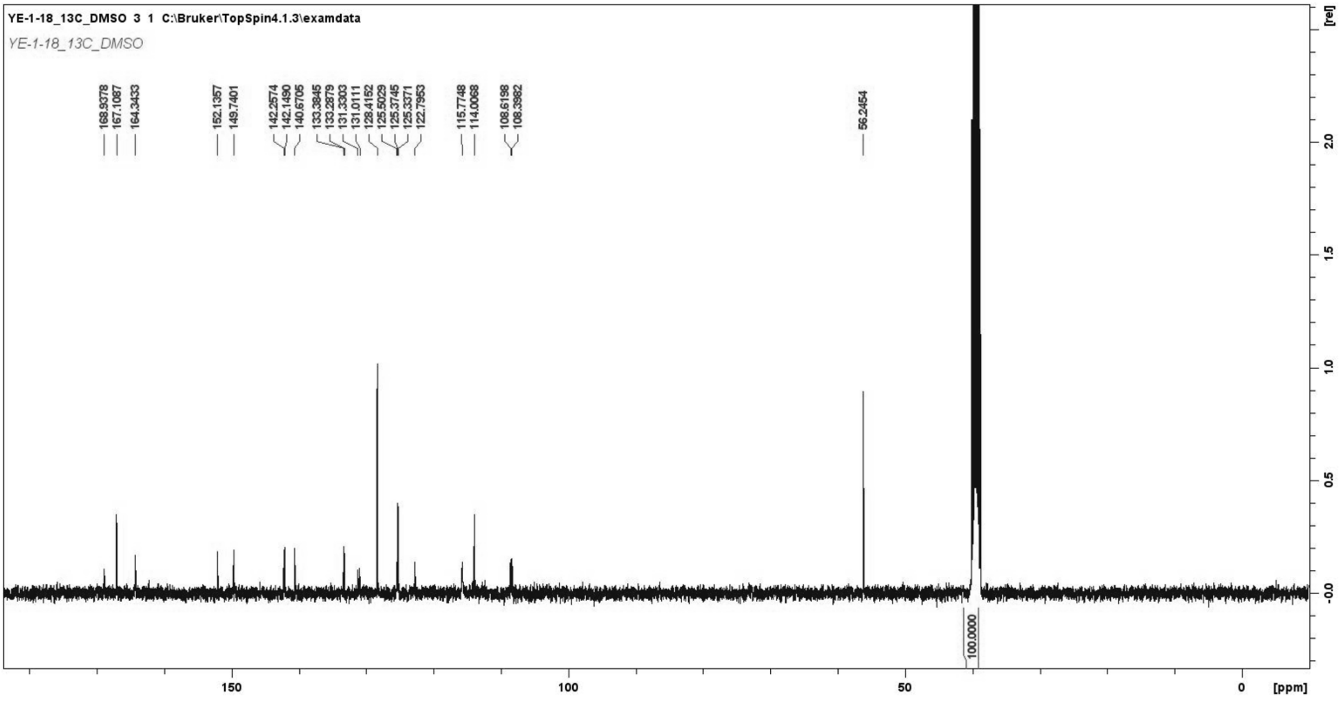

4-(4-Amino-6-((3-fluoro-4-methoxyphenyl)amino)-1,3,5-triazin-2-yl)benzonitrile (**11e**)

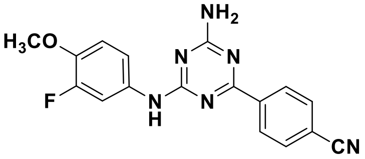

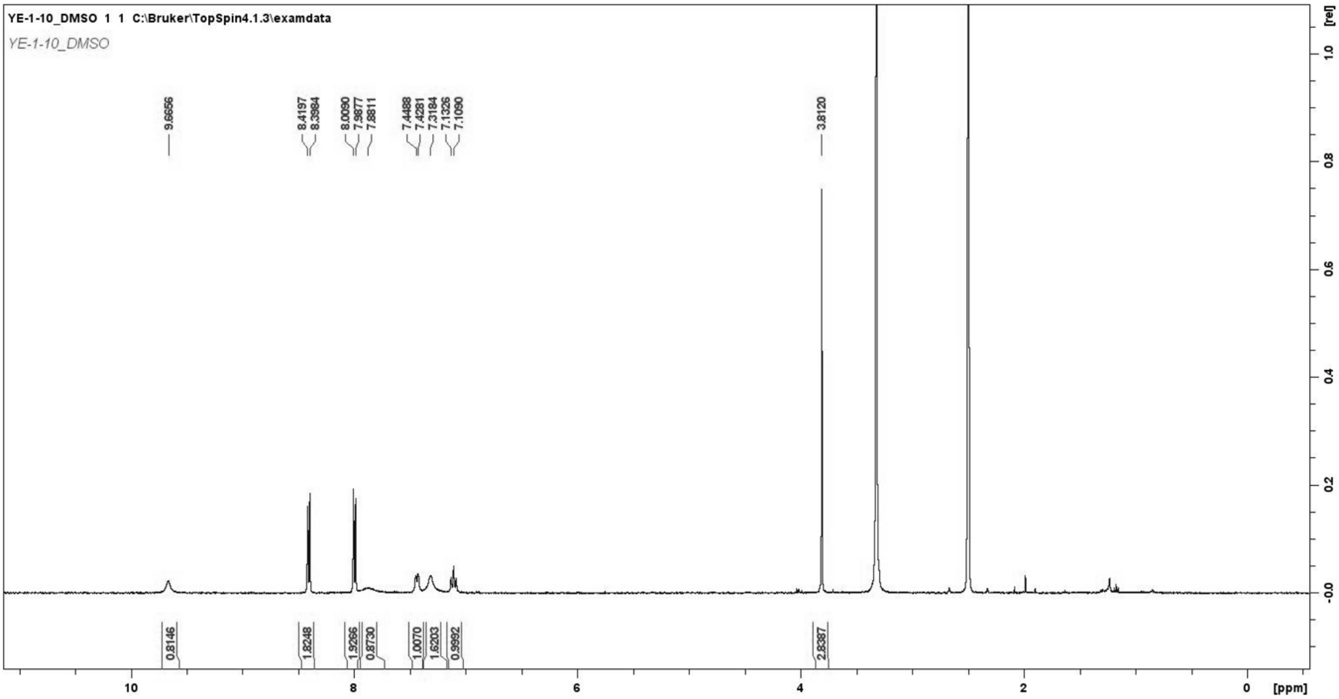

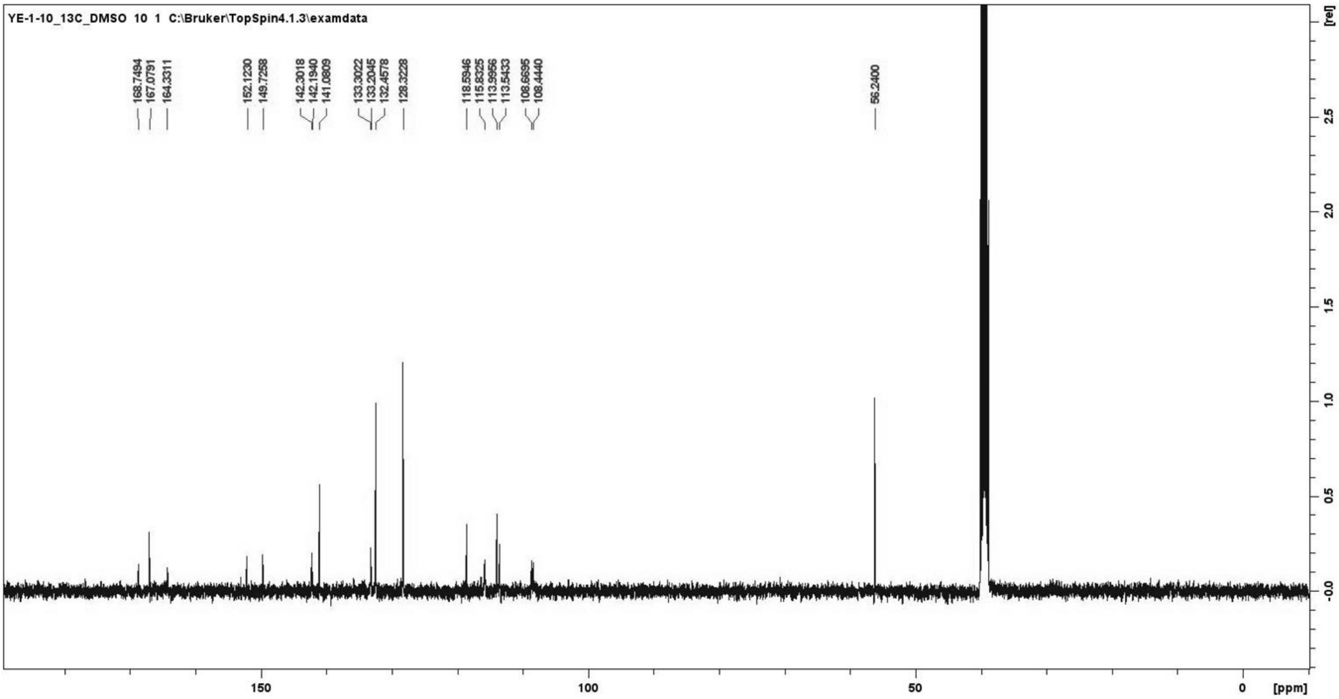

Methyl 4-(4-Amino-6-((3-fluoro-4-methoxyphenyl)amino)-1,3,5-triazin-2-yl)benzoate (**11f**)

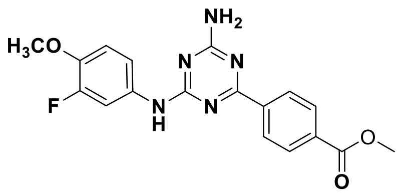

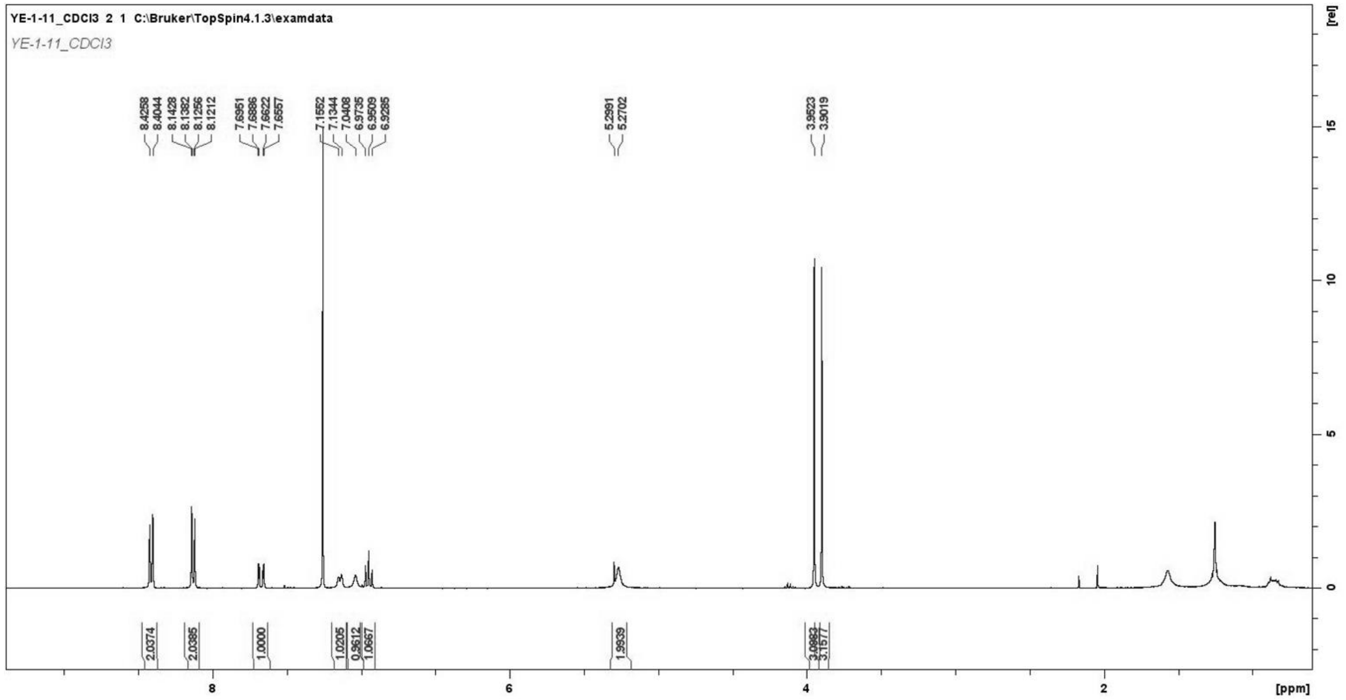

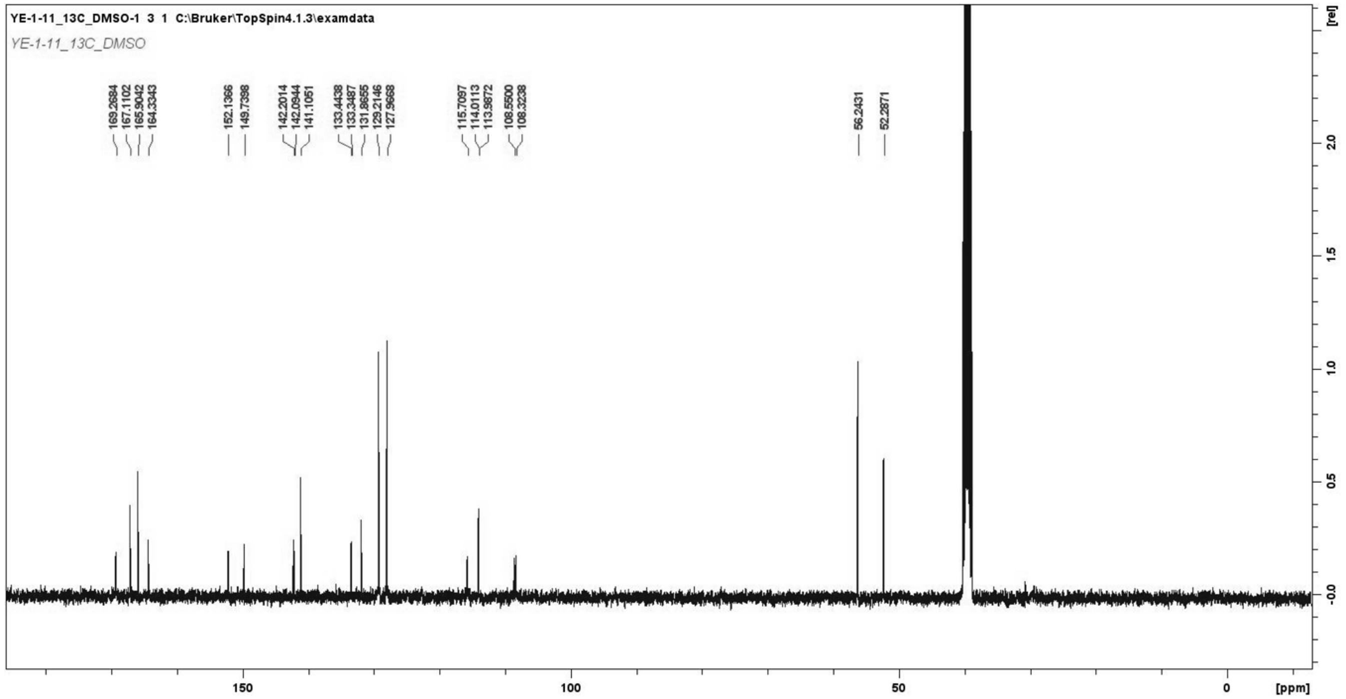

Ethyl 4-(4-Amino-6-((3-fluoro-4-methoxyphenyl)amino)-1,3,5-triazin-2-yl)benzoate (**11g**)

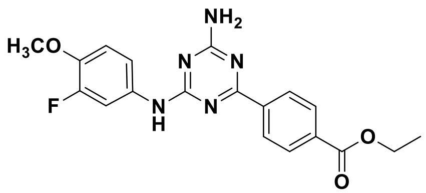

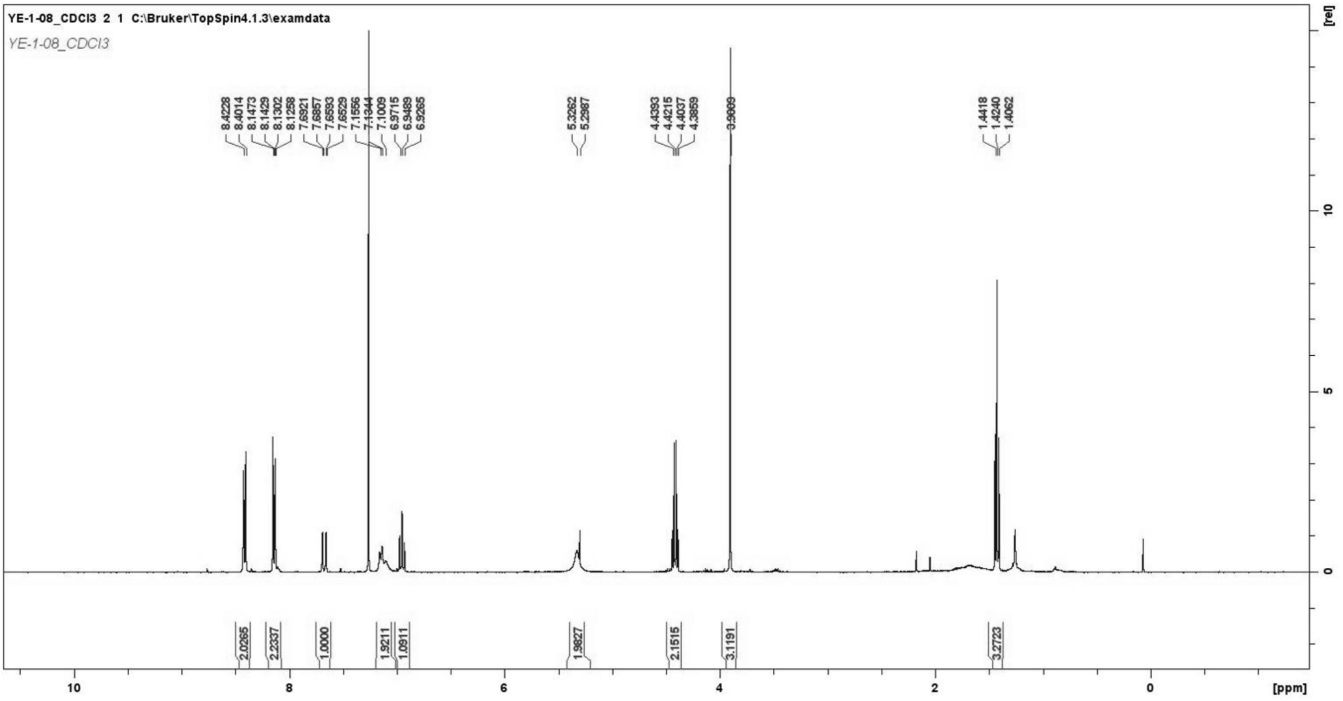

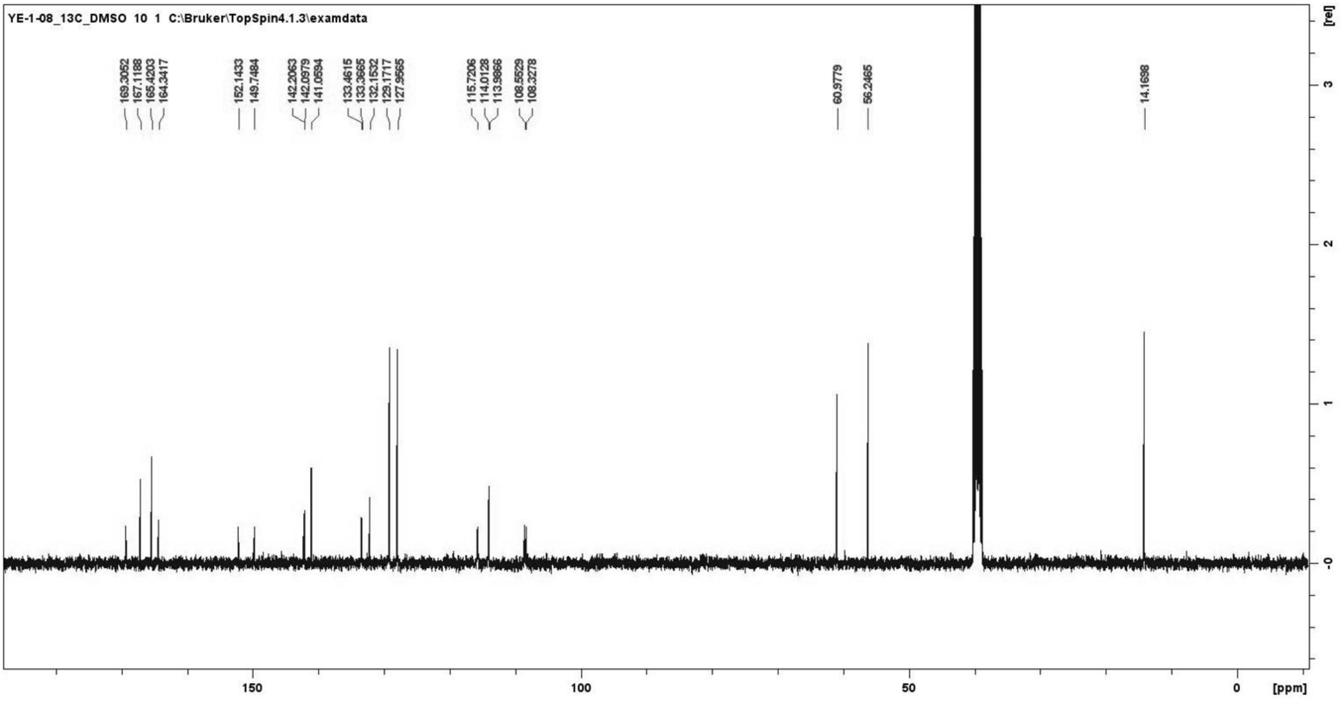

4-(4-Amino-6-((3-fluoro-4-methoxyphenyl)amino)-1,3,5-triazin-2-yl)-2,6-difluorophenol (**11h**)

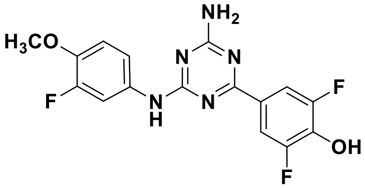

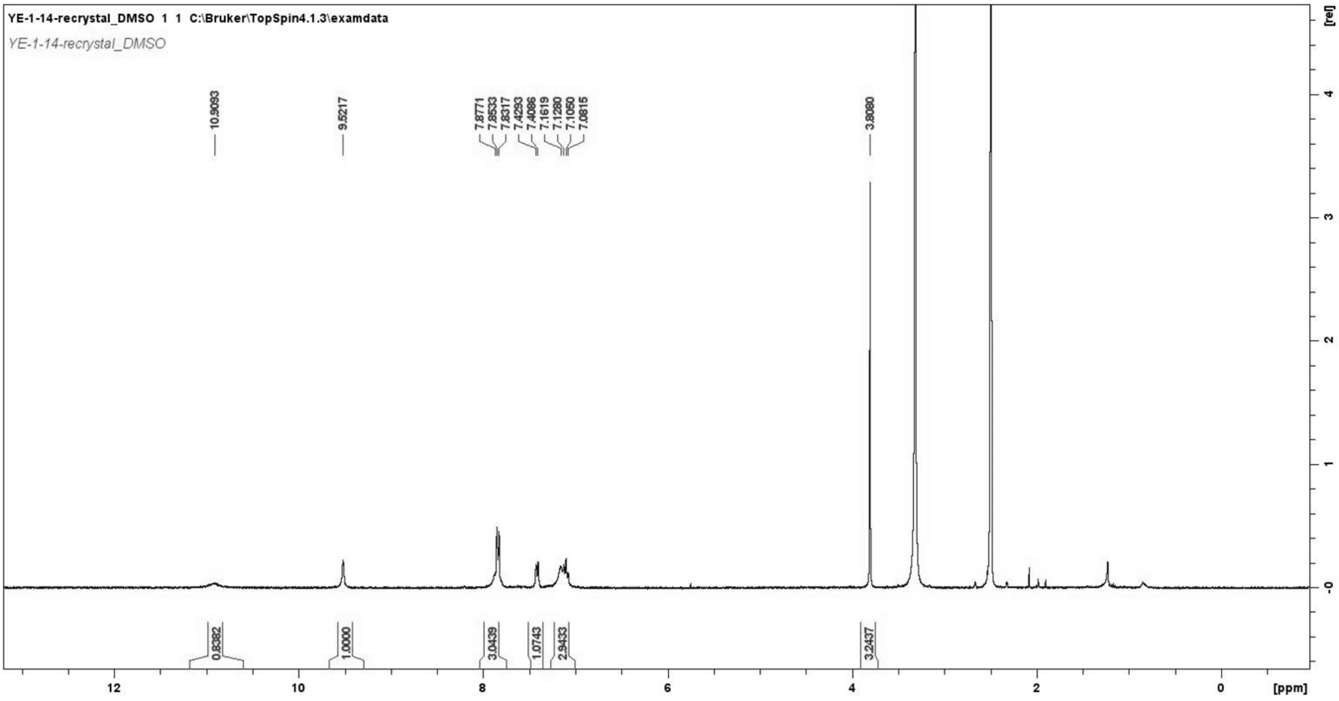

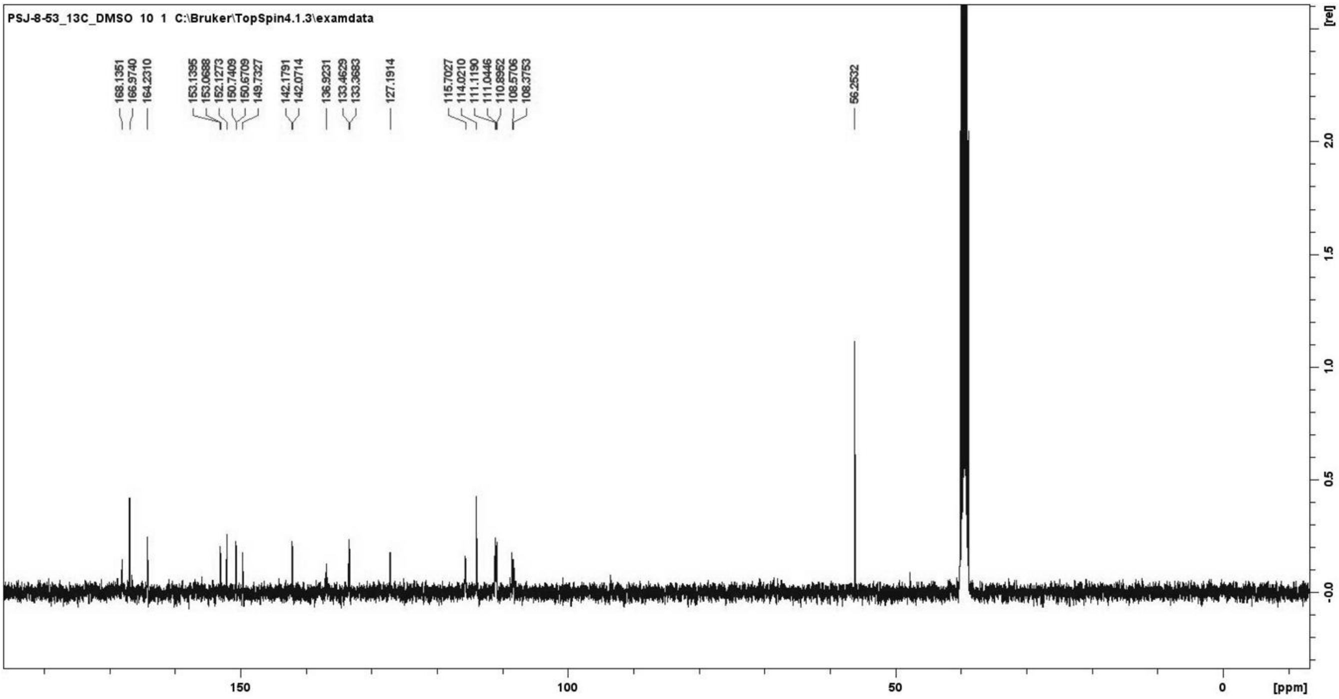

6-(4-Fluoro-3-nitrophenyl)-N^2-(3-fluoro-4-methoxyphenyl)-1,3,5-triazine-2,4-diamine (**11i**)

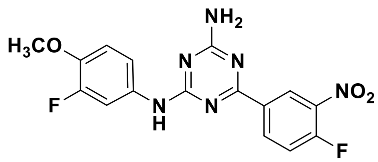

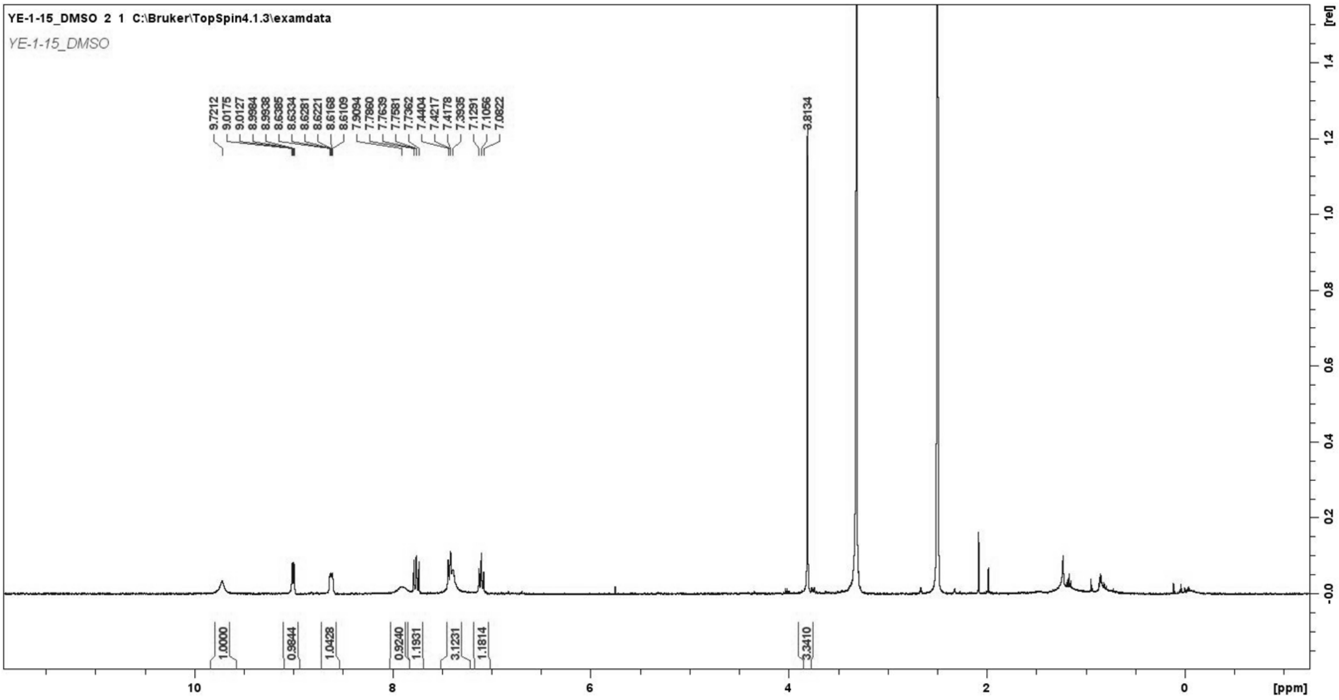

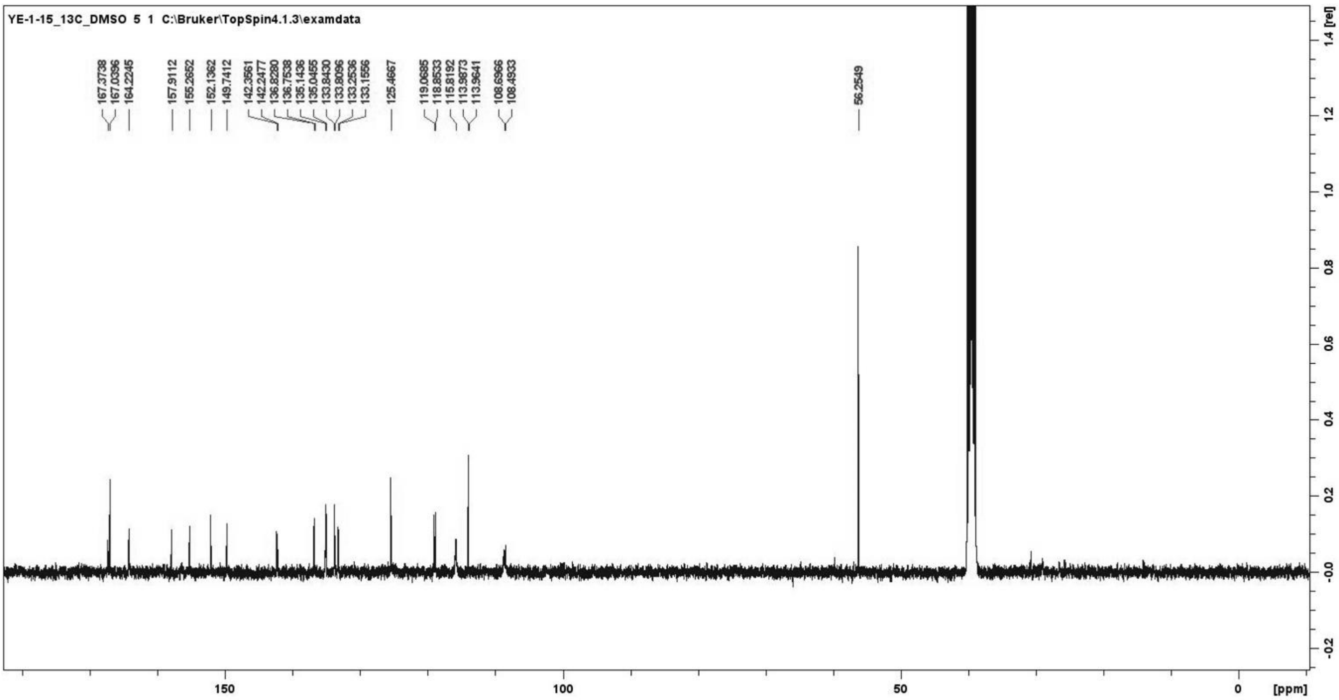

Figure S2. HR-MS Copies of Compound **9a-g** and **11a-i**

4-(4-Amino-6-((3-fluoro-4-methoxyphenyl)amino)-1,3,5-triazin-2-yl)phenol (**9a**)

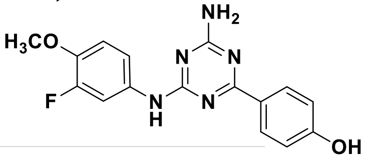

Isotope modeling _ Sample 16

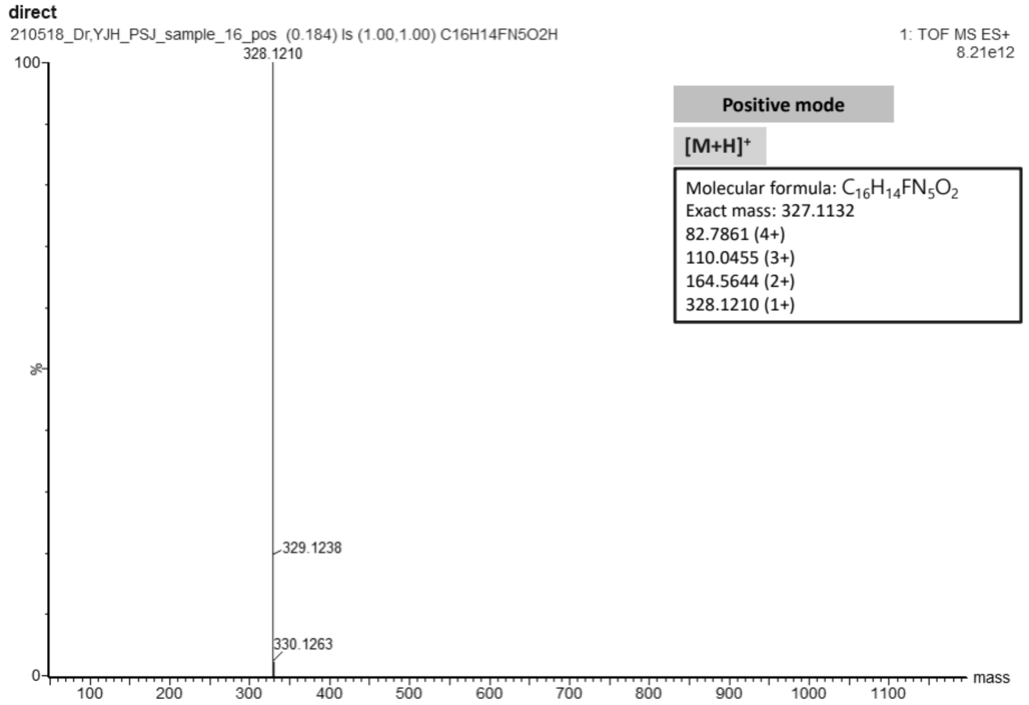

Chromatogram_ Positive mode_ sample

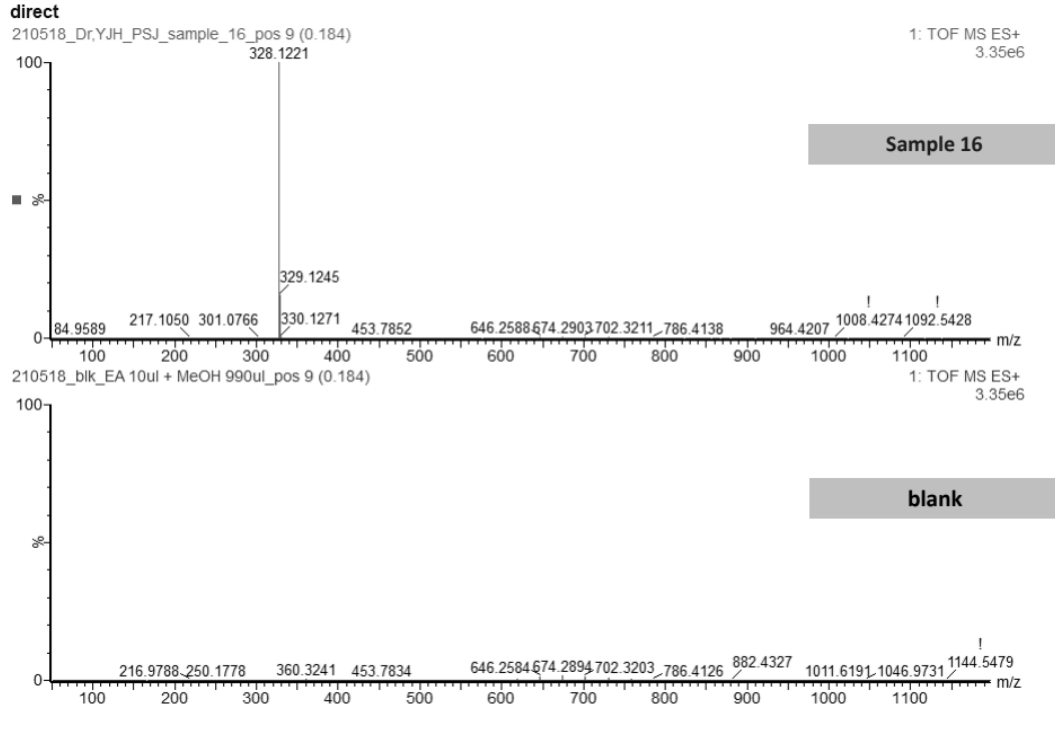

4-(4-Amino-6-((3,5-dimethoxyphenyl)amino)-1,3,5-triazin-2-yl)phenol (**9b**)

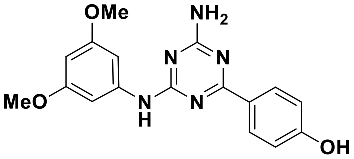

Isotope Modeling: KYC-4-53 (C17H17N5O3, Exact MS 339.1331)

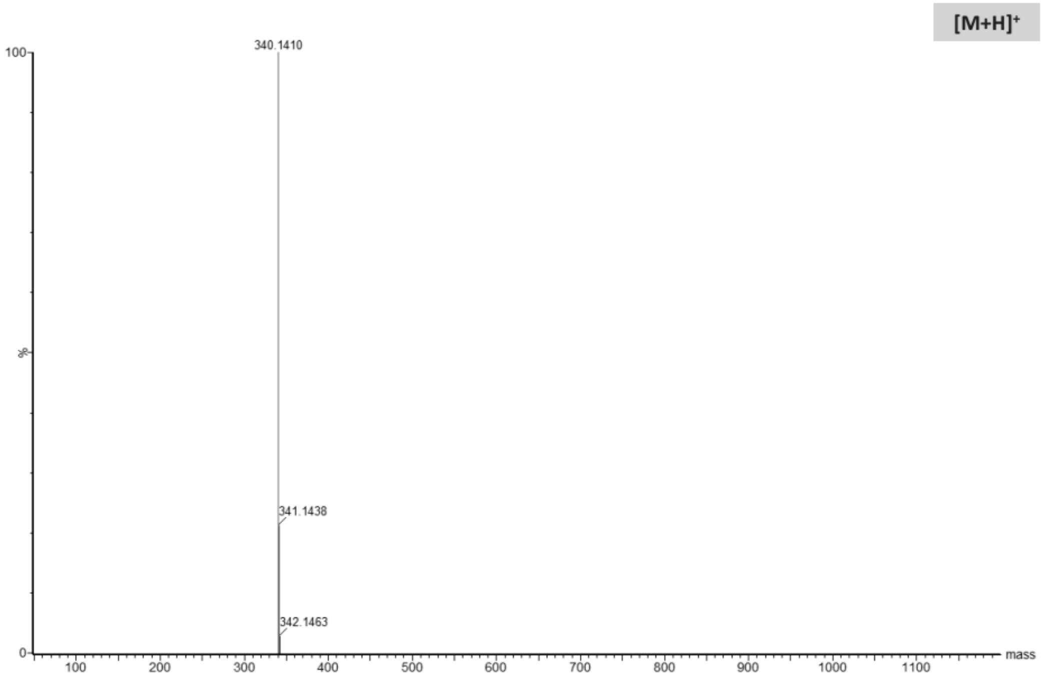

Mass Spectrum: KYC-4-53 (Positive mode)

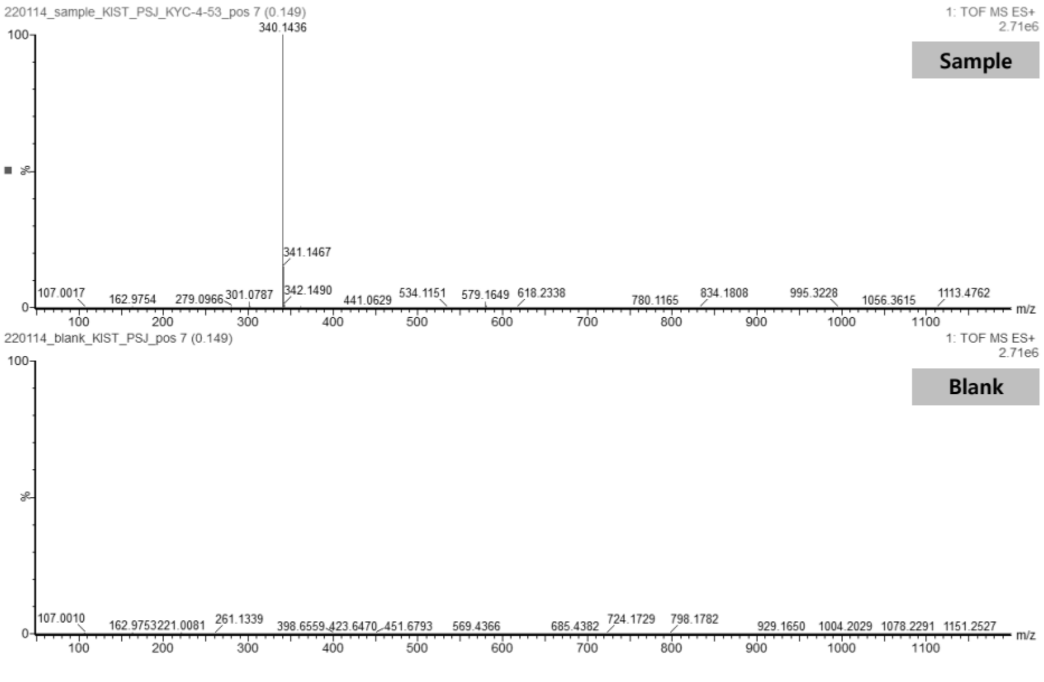

4-(4-Amino-6-((4-chloro-3-methoxyphenyl)amino)-1,3,5-triazin-2-yl)phenol (**9c**)

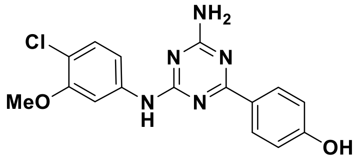

Isotope Modeling: KYC-4-58 (C16H14ClN5O2, Exact MS 343.0836)

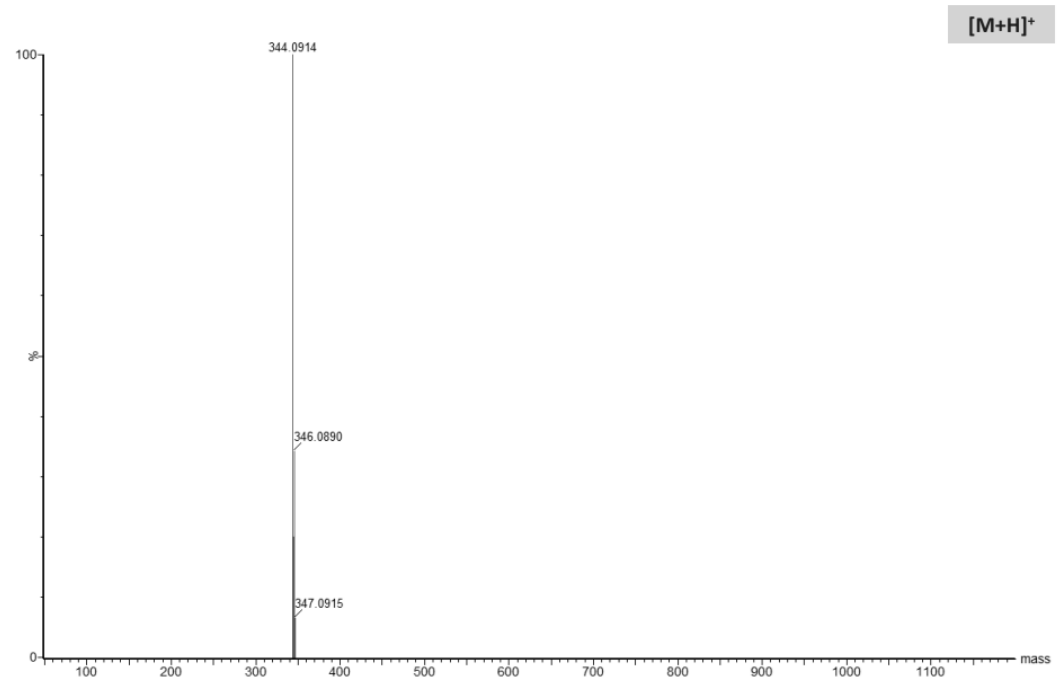

Mass Spectrum: KYC-4-58 (Positive mode)

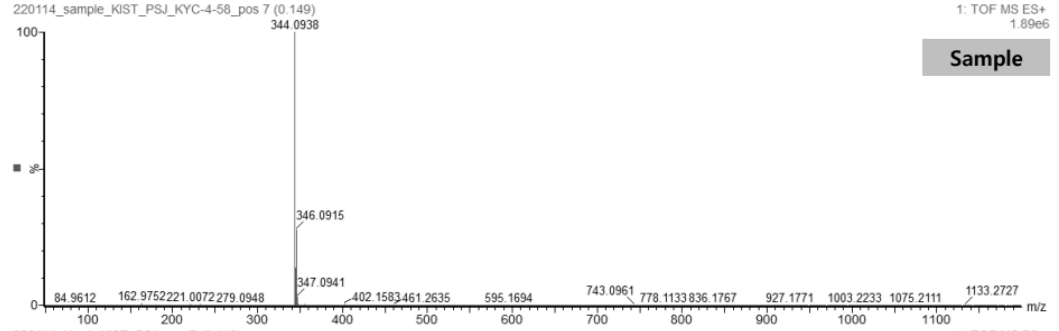

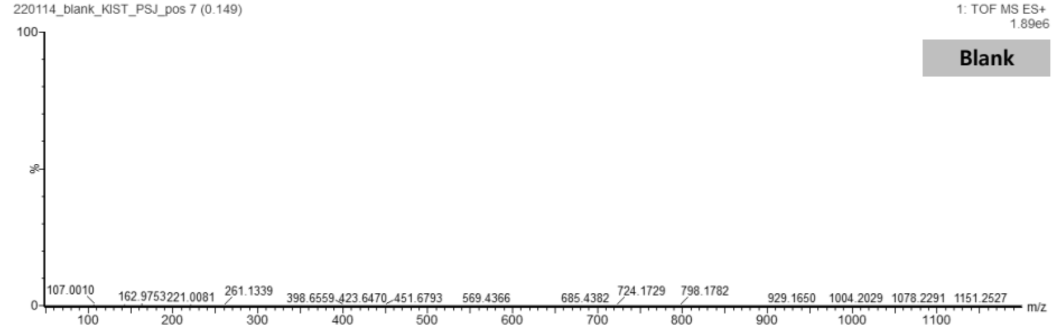

4-(4-Amino-6-((3-(trifluoromethyl)phenyl)amino)-1,3,5-triazin-2-yl)phenol (**9d**)

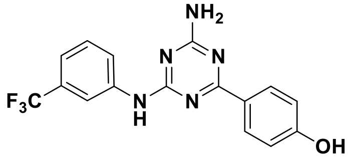

Isotope Modeling: KYC-4-63 (C16H12F3N5O, Exact MS 347.0994)

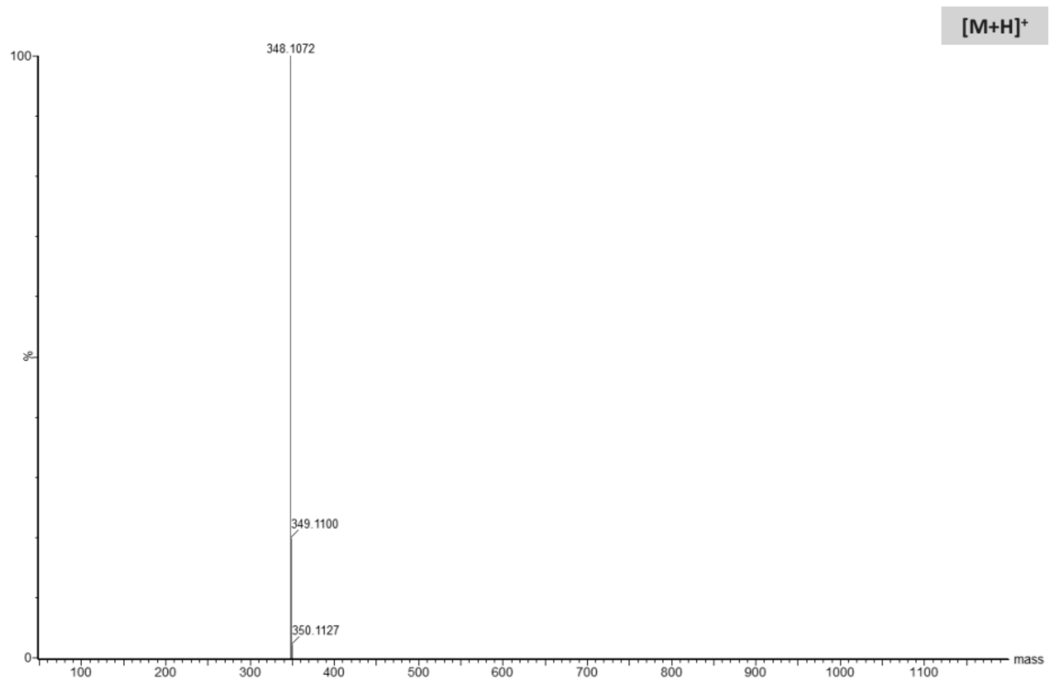

Mass Spectrum: KYC-4-63 (Positive mode)

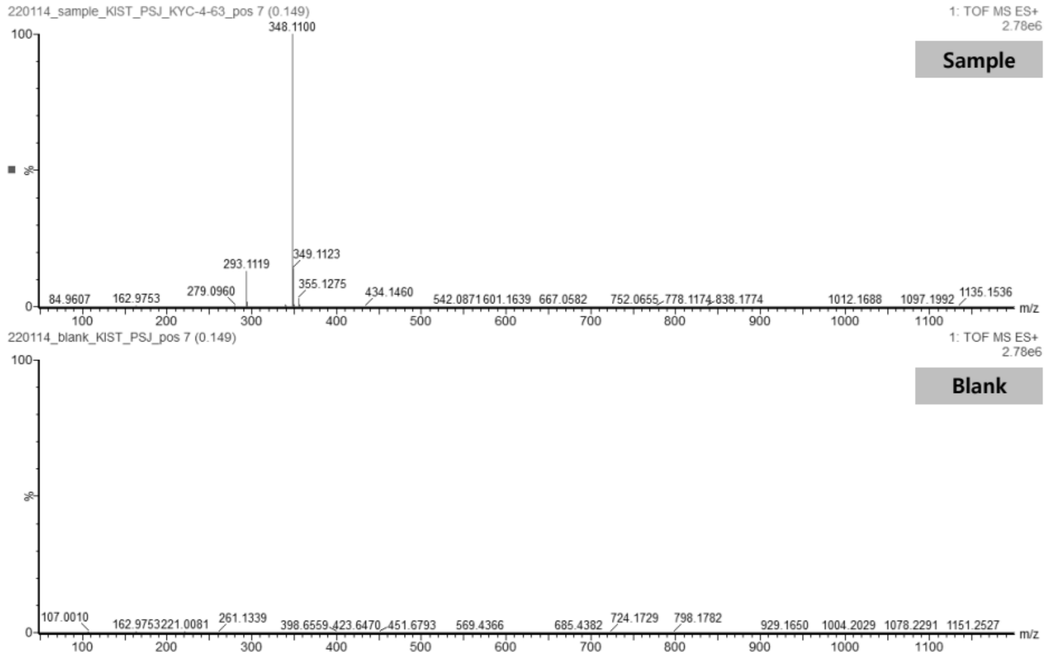

4-(4-Amino-6-((3,5-dimethylphenyl)amino)-1,3,5-triazin-2-yl)phenol (**9e**)

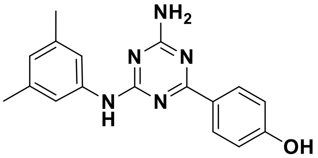

Isotope Modeling: KYC-4-69 (C17H17N5O, Exact MS 307.1433)

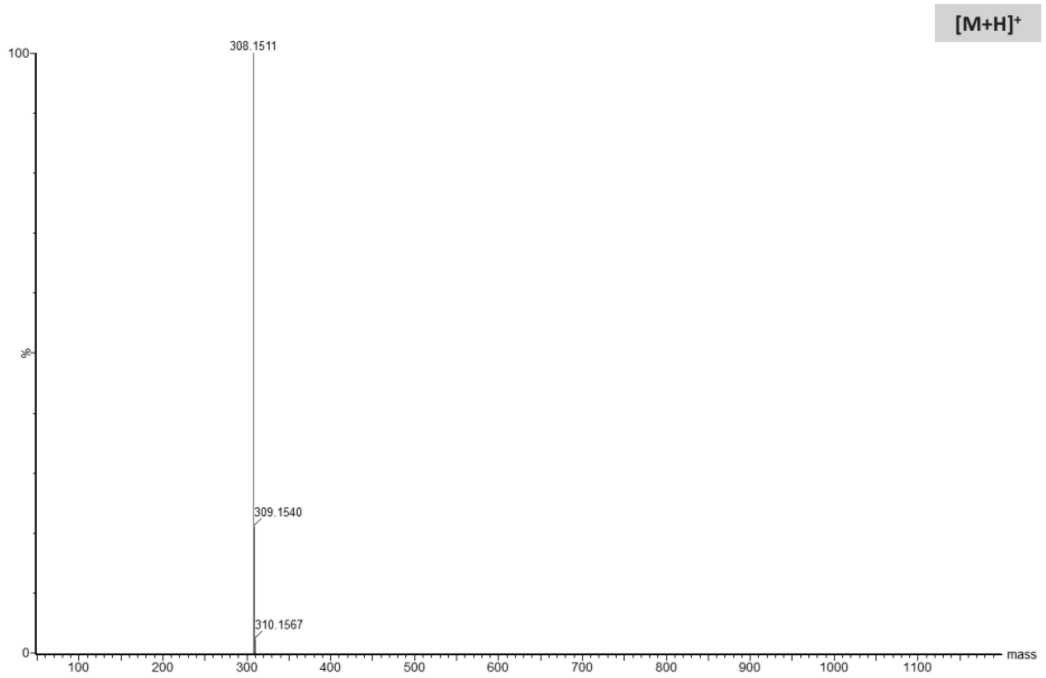

Mass Spectrum: KYC-4-69 (Positive mode)

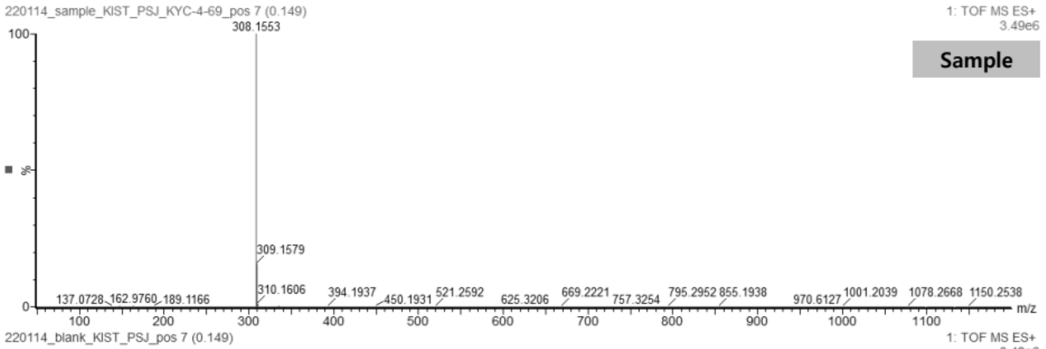

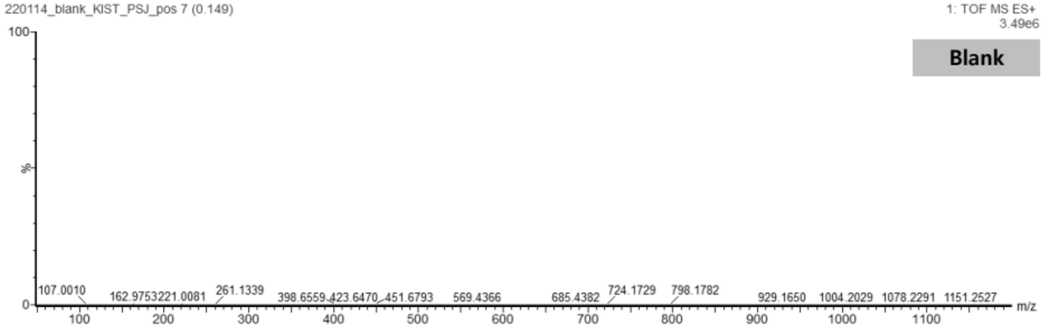

4-(4-Amino-6-((4-(piperidin-1-yl)phenyl)amino)-1,3,5-triazin-2-yl)phenol (**9f**)

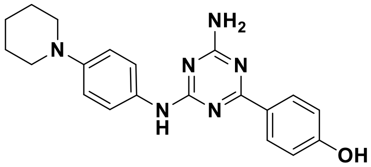

Isotope Modeling: KYC-4-79 ($C_{20}H_{22}N_6O$, Exact MS 362.1855)

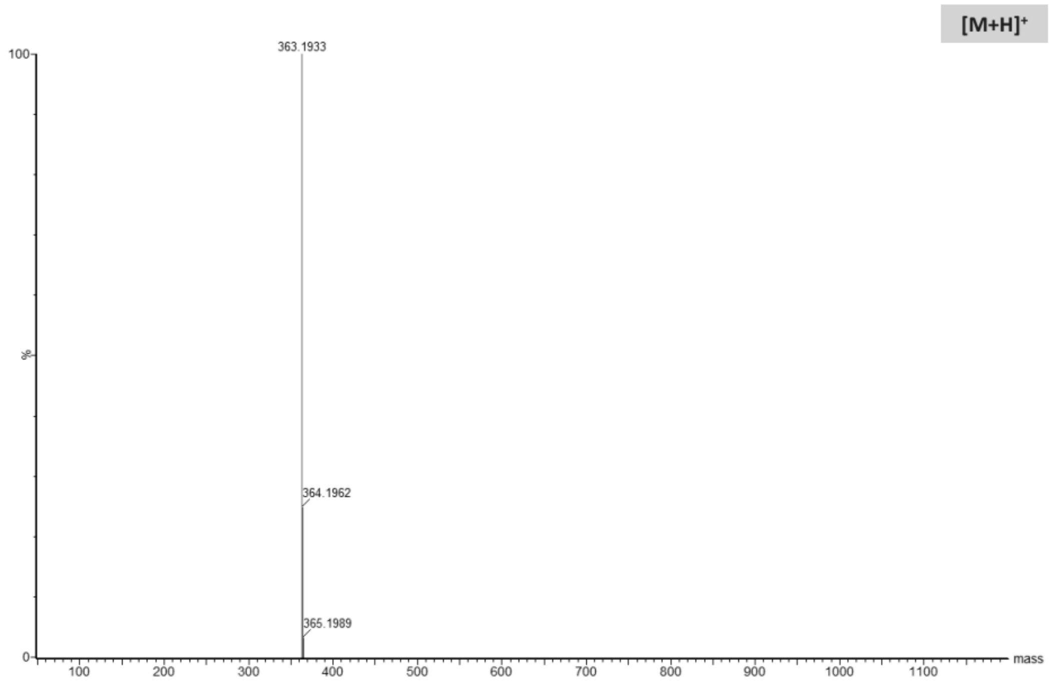

Mass Spectrum: KYC-4-79 (Positive mode)

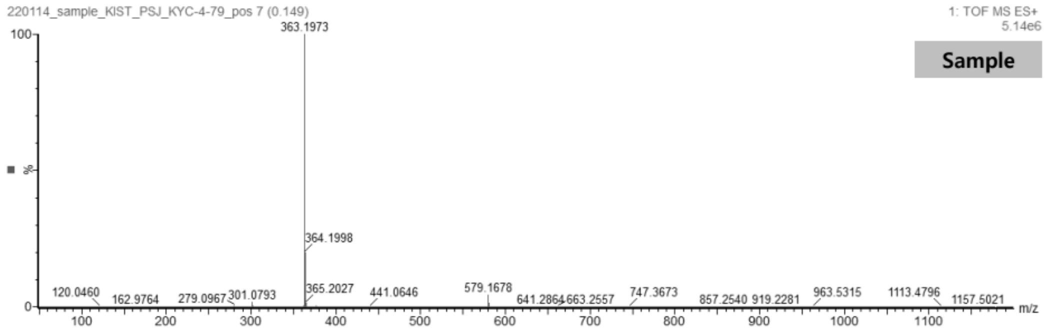

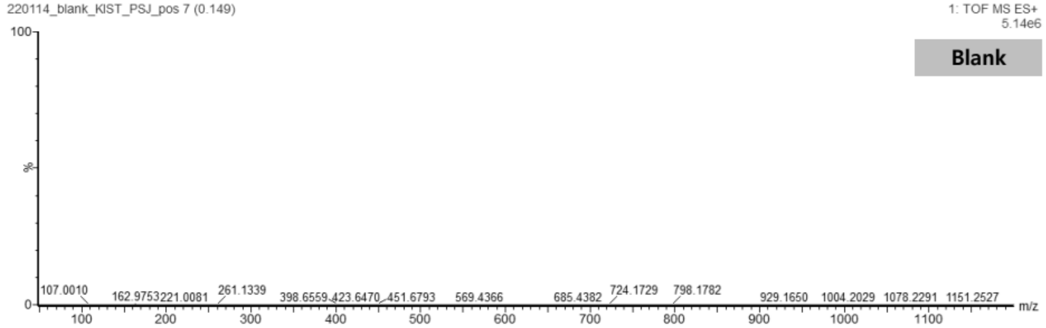

4-(4-Amino-6-((2,4-dimethylphenyl)amino)-1,3,5-triazin-2-yl)phenol (**9g**)

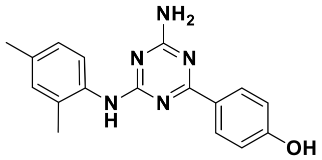

Isotope Modeling: KYC-4-82 (C17H17N5O, Exact MS 307.1433)

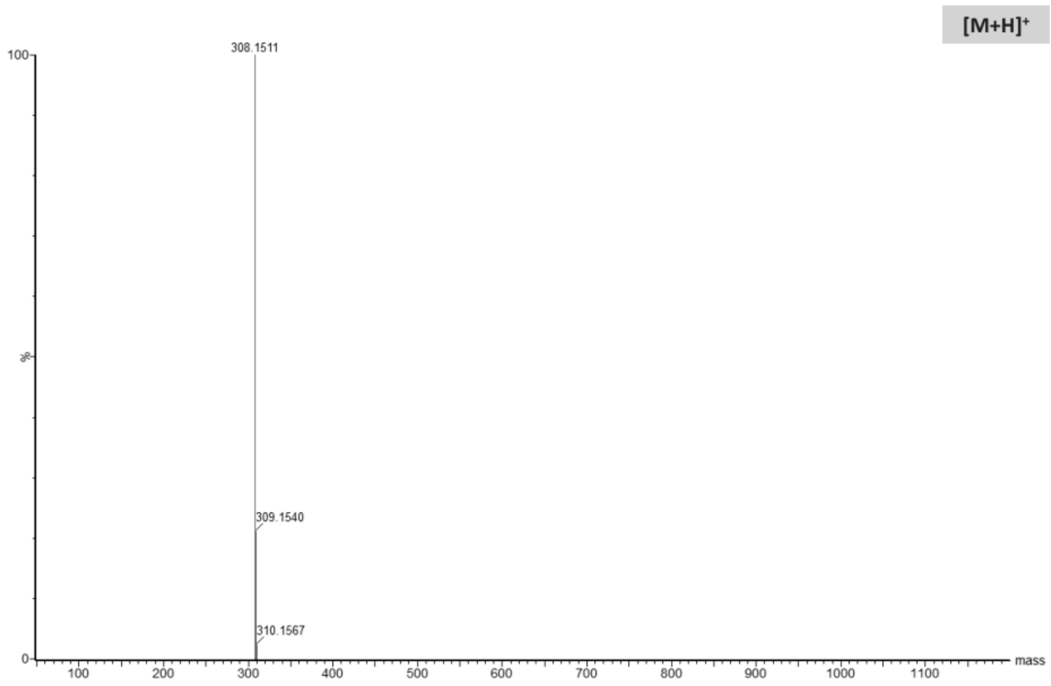

Mass Spectrum: KYC-4-82 (Positive mode)

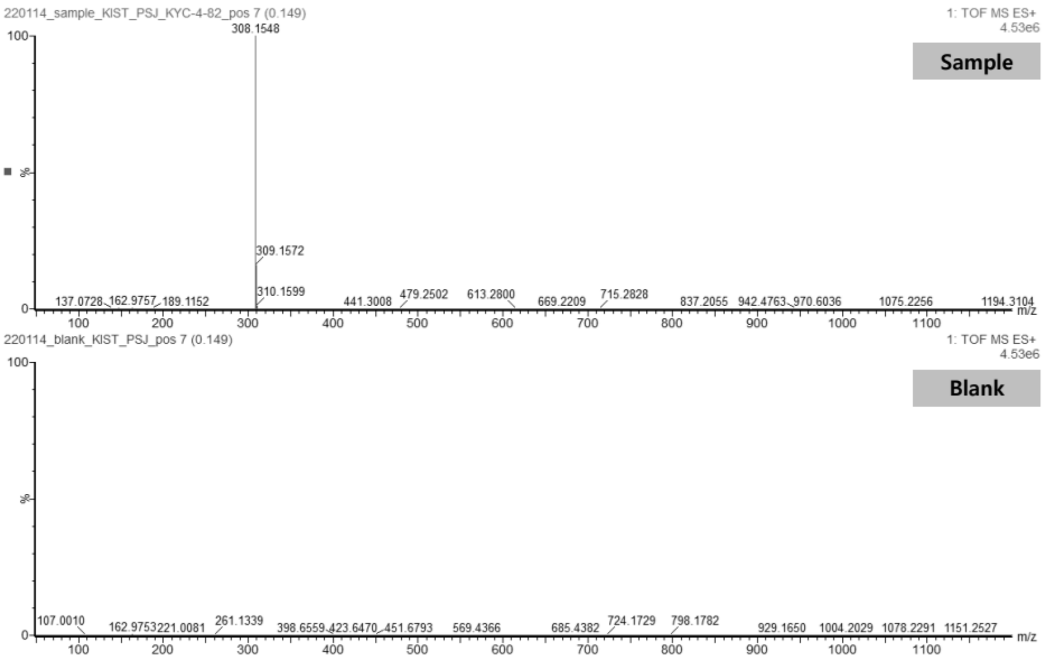

N[2]-(3-Fluoro-4-methoxyphenyl)-6-(4-methoxyphenyl)-1,3,5-triazine-2,4-diamine (**11a**)

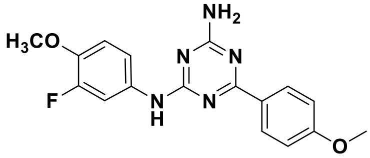

Compound Table

Compound Label	RT	Mass	Abund	Formula	Tgt Mass	Diff (ppm)
Cpd 1: C17 H16 F N5 O2	0.242	341.1295	681105	C17 H16 F N5 O2	341.1288	1.96

Compound Label	*m/z*	RT	Algorithm	Mass
Cpd 1: C17 H16 F N5 O2	342.1368	0.242	Find By Formula	341.1295

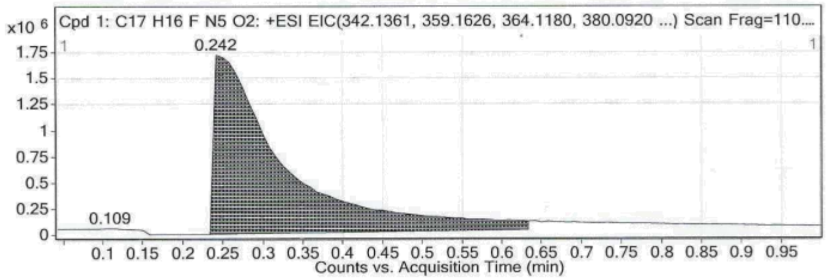

MS Spectrum

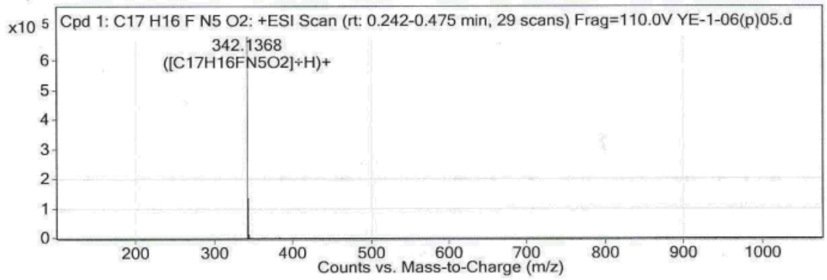

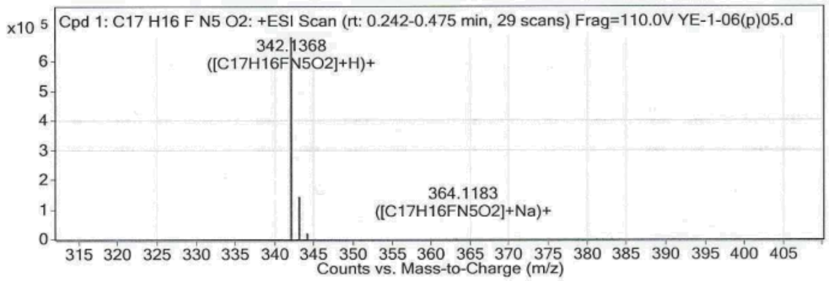

MS Spectrum Peak List

m/z	*Calc m/z*	Diff(ppm)	z	Abund	Formula	Ion
342.1368	342.1361	-2.13	1	681105.13	C17H16FN5O2	(M+H)+
343.1393	343.1389	-1.15	1	135914.66	C17H16FN5O2	(M+H)+
344.1418	344.1415	-0.93	1	12011.48	C17H16FN5O2	(M+H)+
345.1447	345.1439	-2.33	1	1009.78	C17H16FN5O2	(M+H)+
364.1183	364.118	-0.66	1	94.29	C17H16FN5O2	(M+Na)+
380.0932	380.092	-3.17	1	62.38	C17H16FN5O2	(M+K)+

*N*²-(3-Fluoro-4-methoxyphenyl)-6-(4-fluorophenyl)-1,3,5-triazine-2,4-diamine (**11b**)

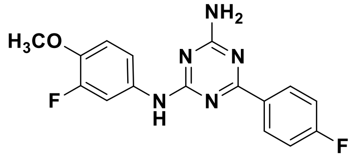

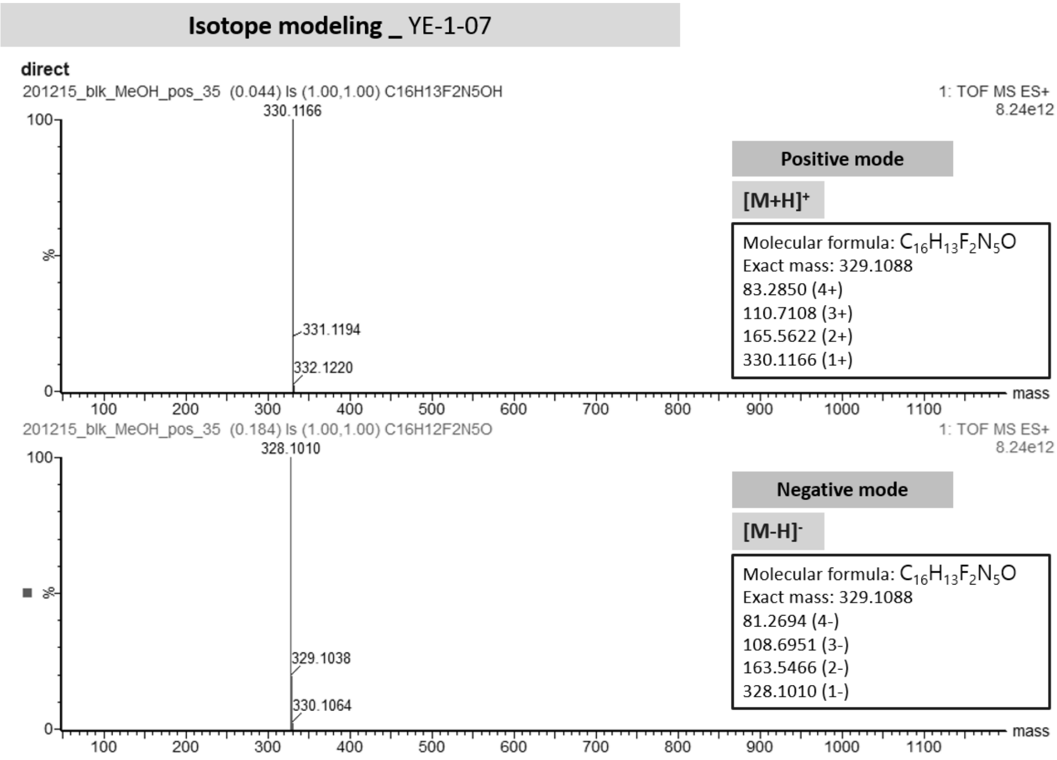

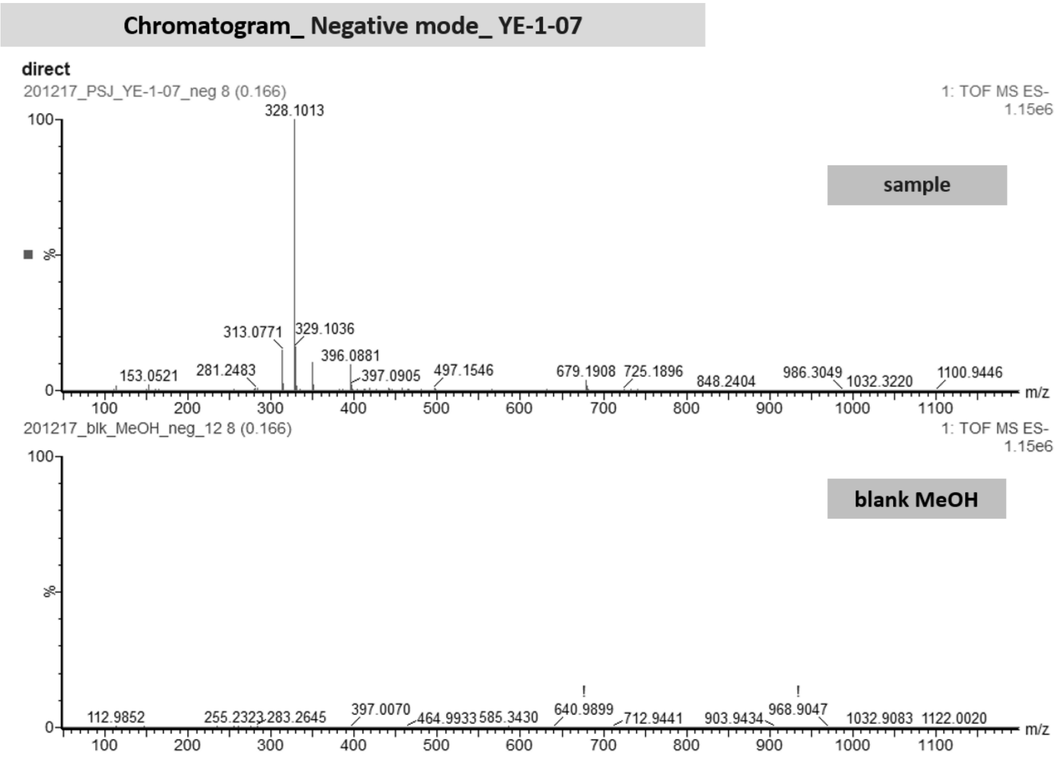

N[2]-(3-Fluoro-4-methoxyphenyl)-6-(4-(trifluoromethoxy)phenyl)-1,3,5-triazine-2,4-diamine (**11c**)

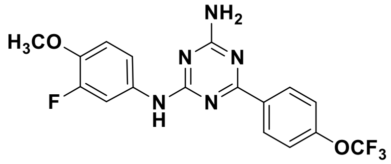

Isotope modeling _ YE-1-09

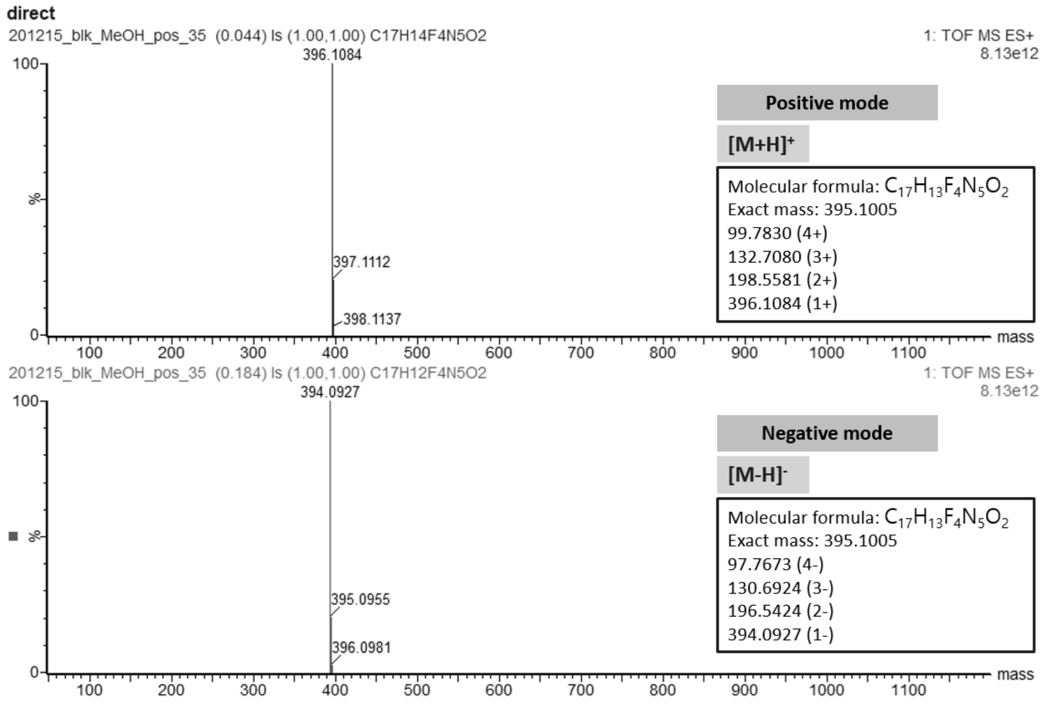

Chromatogram_ Negative mode_ YE-1-09

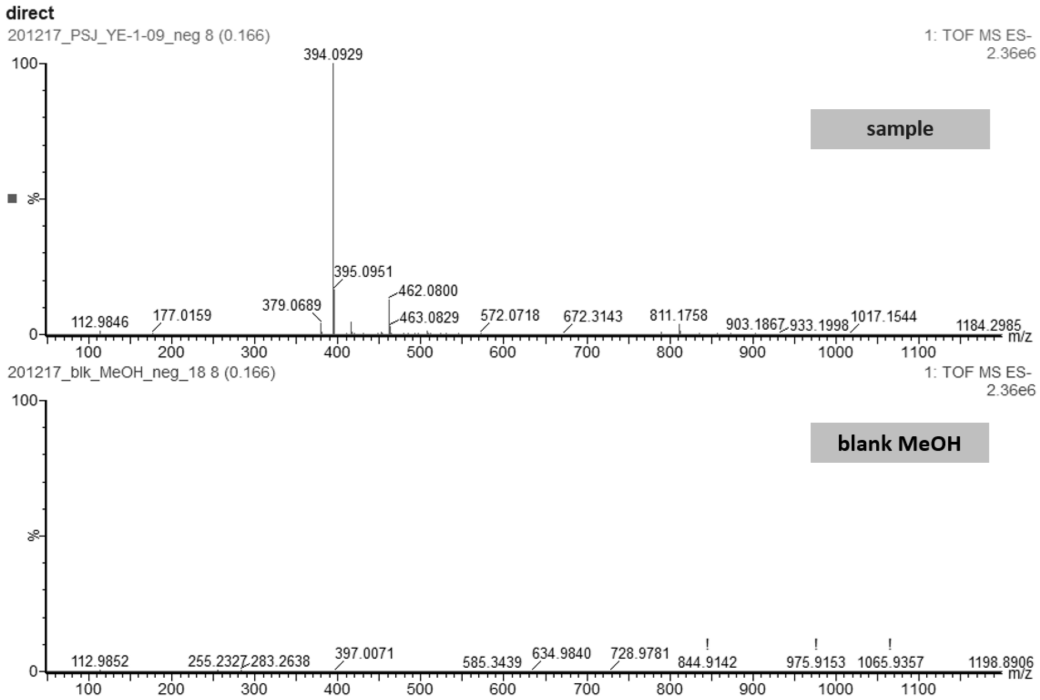

N^2-(3-Fluoro-4-methoxyphenyl)-6-(4-(trifluoromethyl)phenyl)-1,3,5-triazine-2,4-diamine (**11d**)

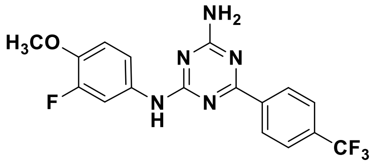

Isotope modeling _ YE-1-18

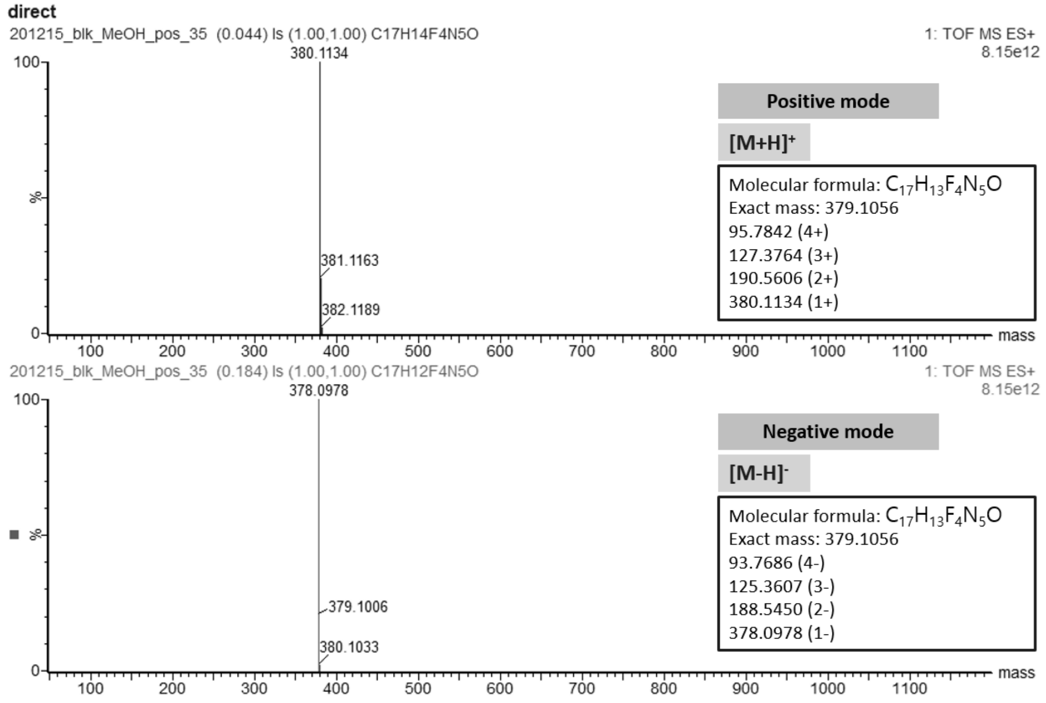

Chromatogram_ Negative mode_ YE-1-18

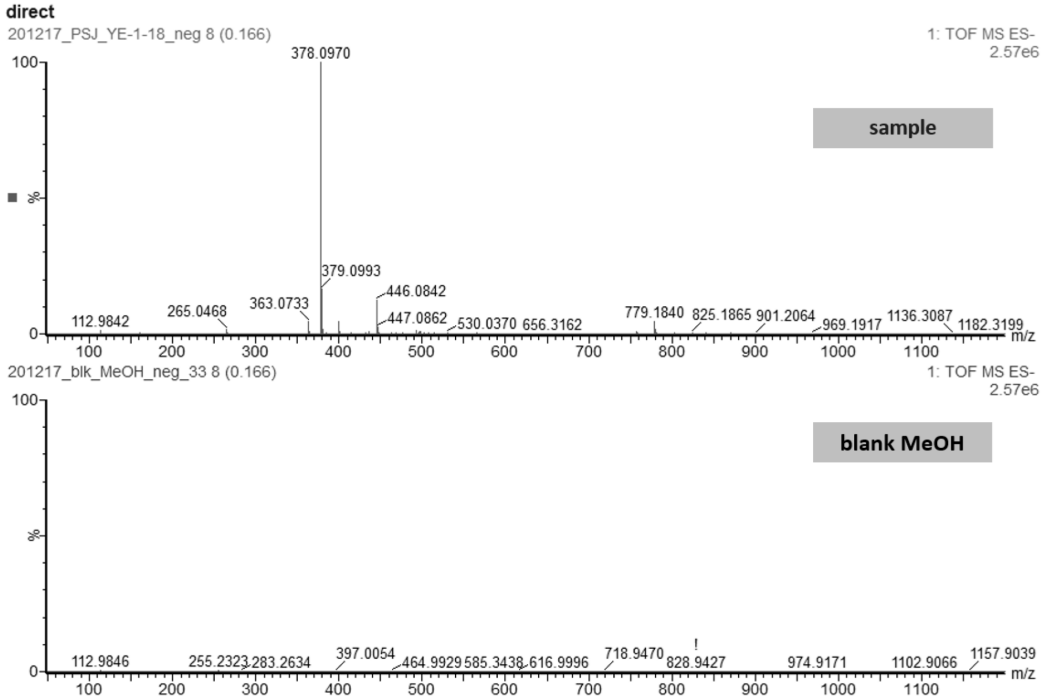

4-(4-Amino-6-((3-fluoro-4-methoxyphenyl)amino)-1,3,5-triazin-2-yl)benzonitrile (**11e**)

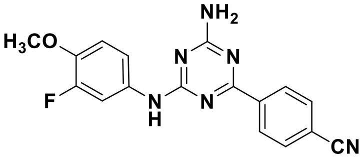

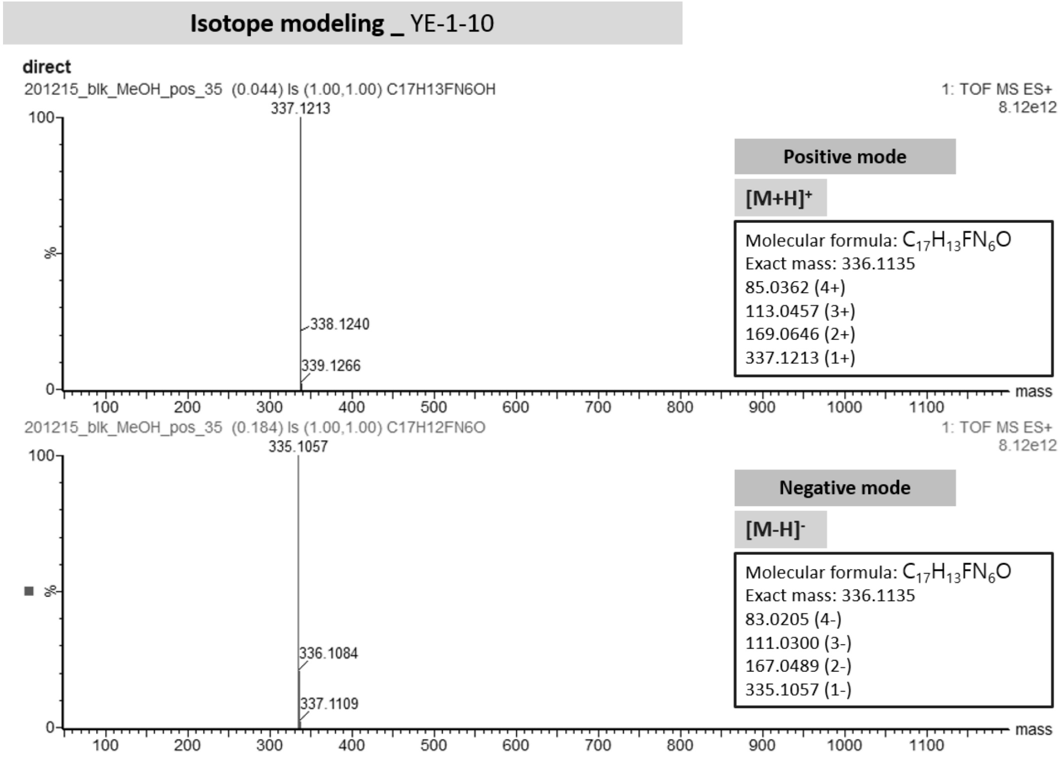

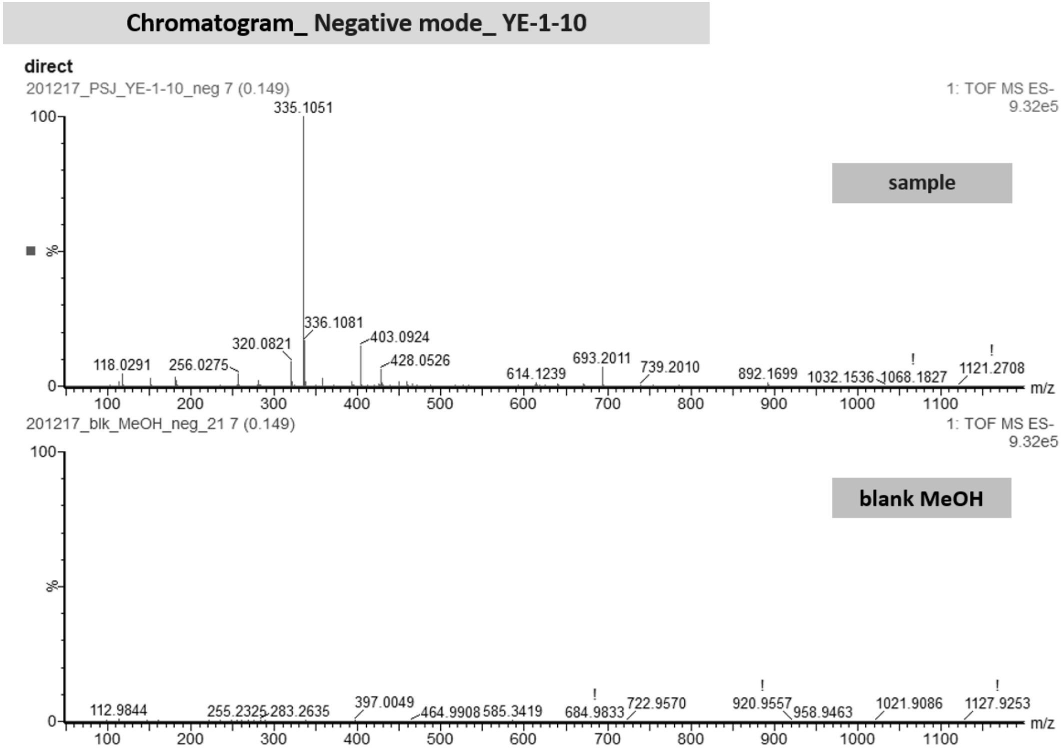

Methyl 4-(4-Amino-6-((3-fluoro-4-methoxyphenyl)amino)-1,3,5-triazin-2-yl)benzoate (**11f**)

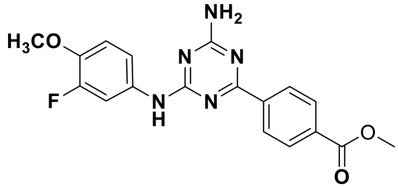

Isotope modeling _ YE-1-11

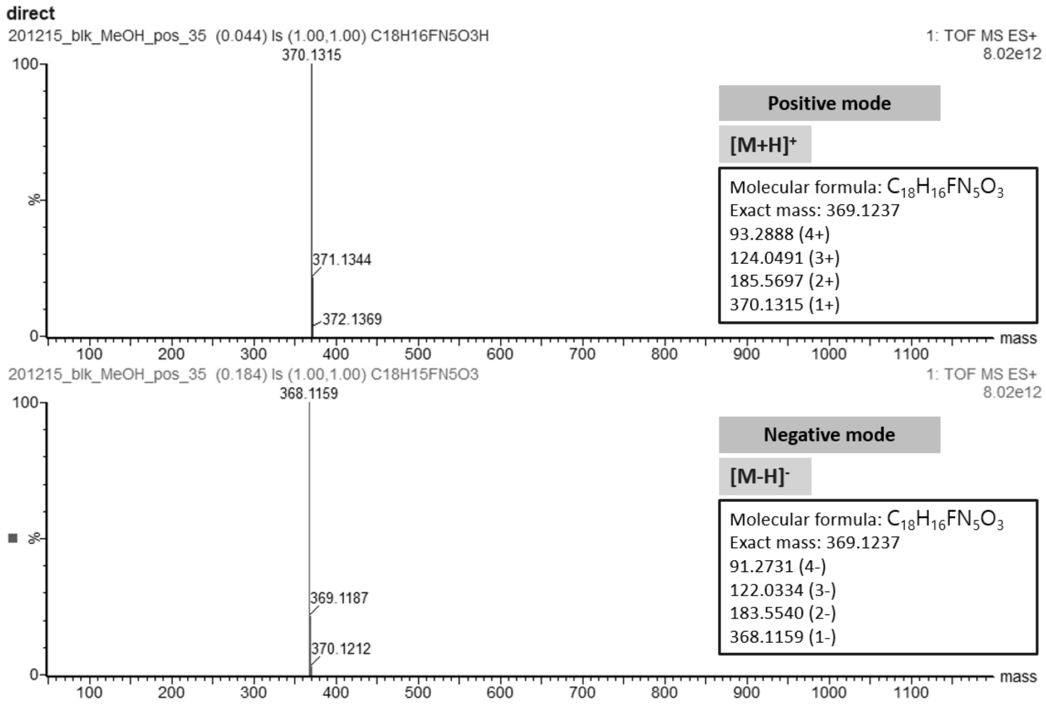

Chromatogram_ Negative mode_ YE-1-11

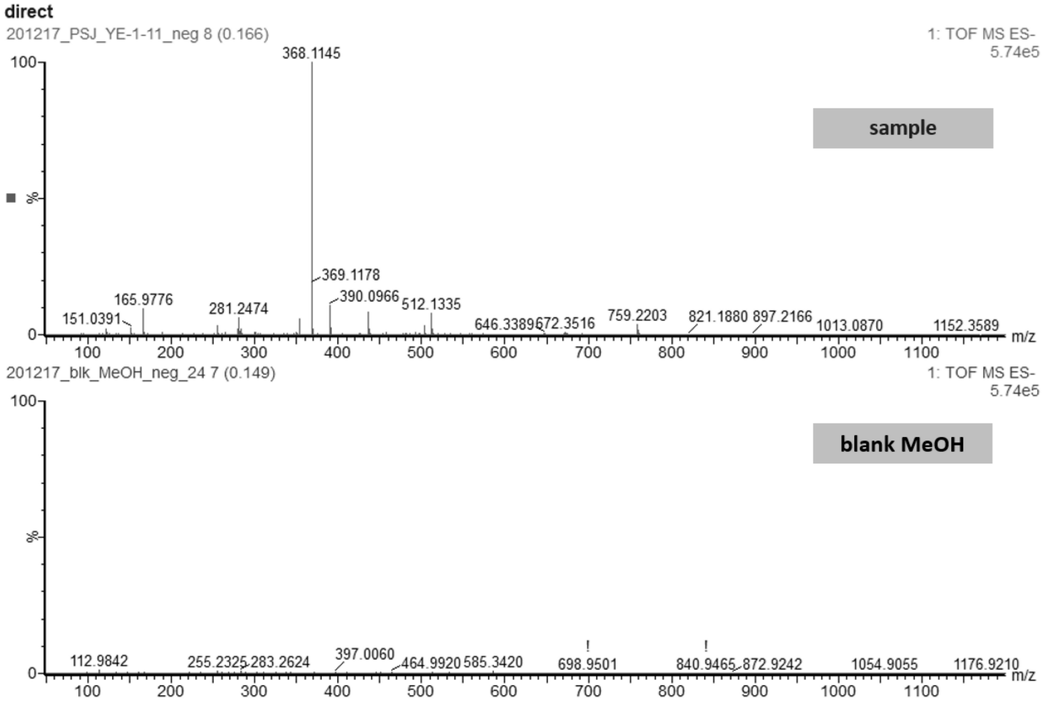

Ethyl 4-(4-Amino-6-((3-fluoro-4-methoxyphenyl)amino)-1,3,5-triazin-2-yl)benzoate (**11g**)

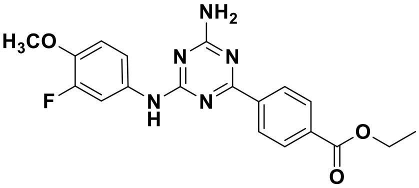

Compound Table

Compound Label	RT	Mass	Abund	Formula	Tgt Mass	Diff (ppm)
Cpd 1: C19 H18 F N5 O3	0.178	383.1395	25410	C19 H18 F N5 O3	383.1394	0.4

Compound Label	*m/z*	RT	Algorithm	Mass
Cpd 1: C19 H18 F N5 O3	384.1468	0.178	Find By Formula	383.1395

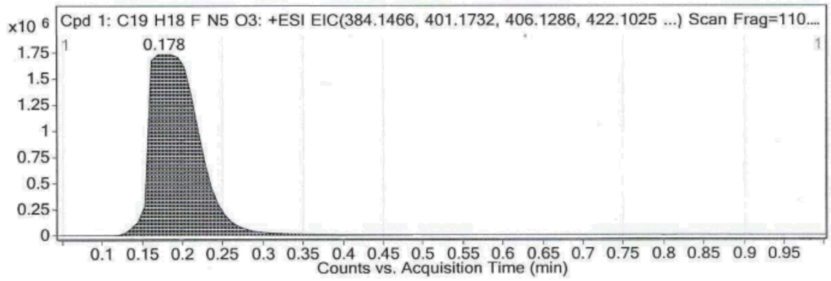

MS Spectrum

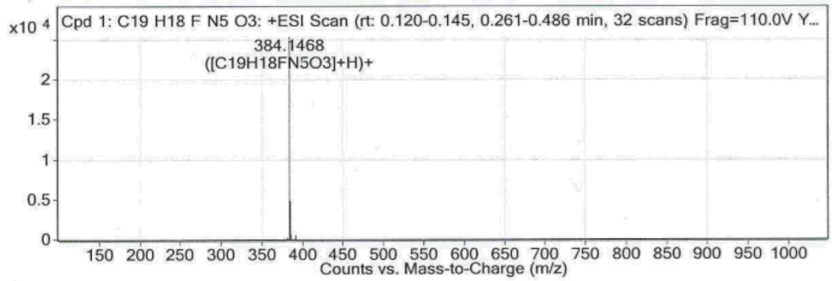

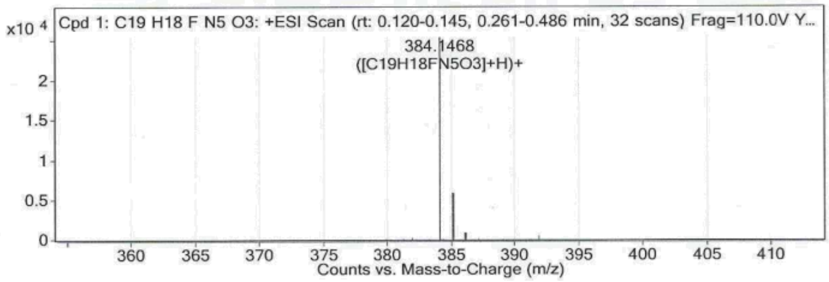

MS Spectrum Peak List

m/z	*Calc m/z*	Diff(ppm)	z	Abund	Formula	Ion
384.1468	384.1466	-0.43	1	25409.76	C19H18FN5O3	(M+H)+
385.1496	385.1495	-0.17	1	4864.49	C19H18FN5O3	(M+H)+
386.1523	386.1521	-0.52	1	582.88	C19H18FN5O3	(M+H)+
387.1566	387.1546	-5.39	1	32.66	C19H18FN5O3	(M+H)+

4-(4-Amino-6-((3-fluoro-4-methoxyphenyl)amino)-1,3,5-triazin-2-yl)-2,6-difluorophenol (**11h**)

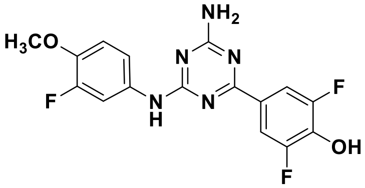

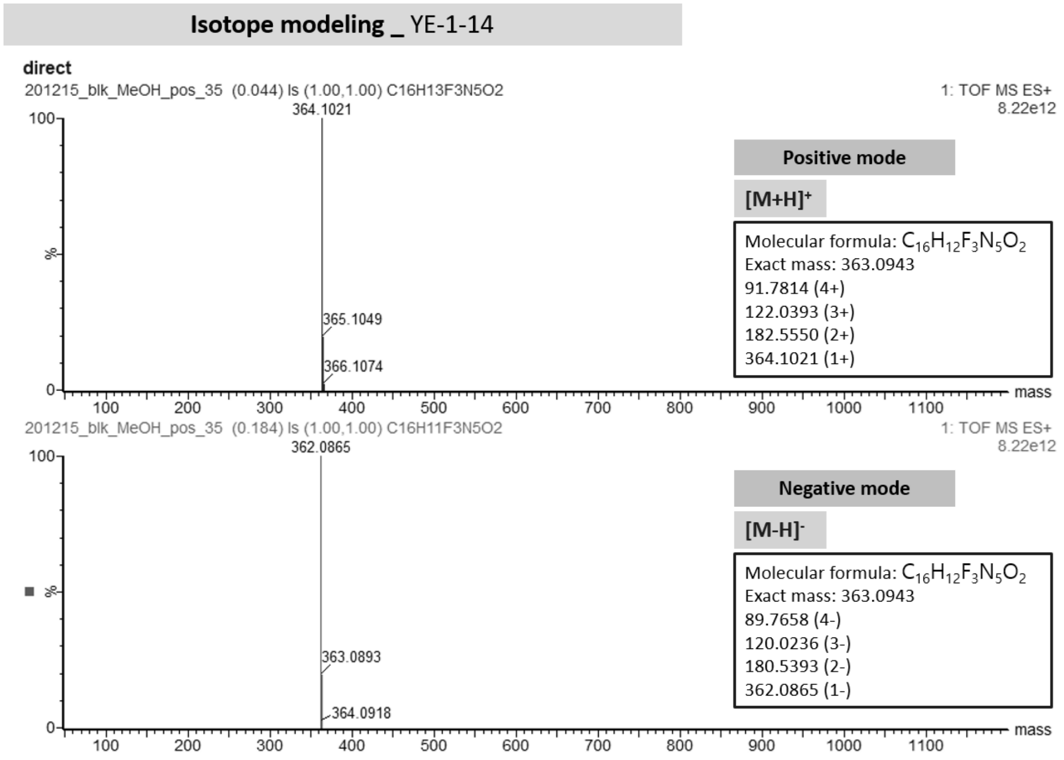

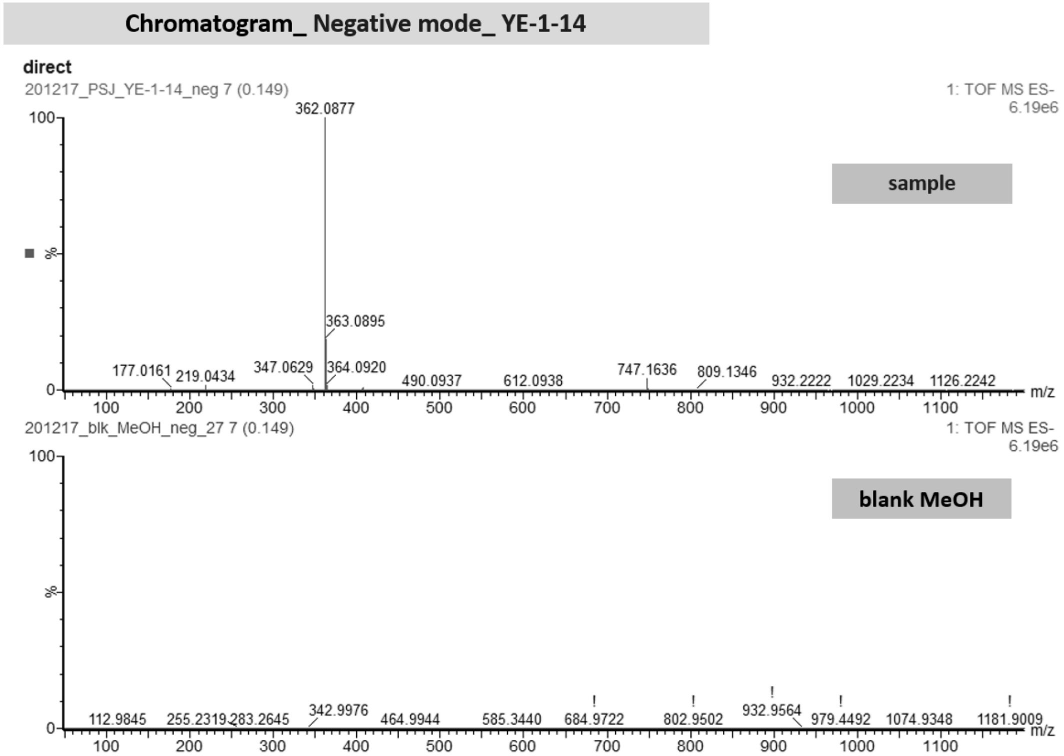

6-(4-Fluoro-3-nitrophenyl)-N^2-(3-fluoro-4-methoxyphenyl)-1,3,5-triazine-2,4-diamine (**11i**)

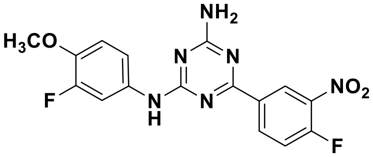

Isotope modeling _ YE-1-15

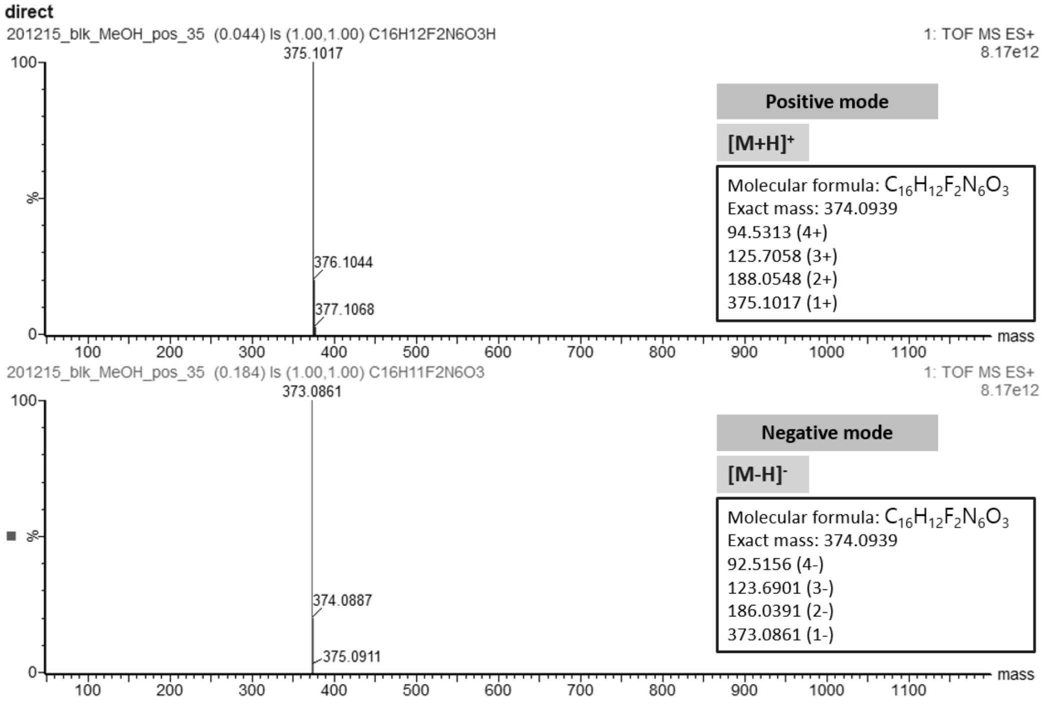

Chromatogram_ Negative mode_ YE-1-15

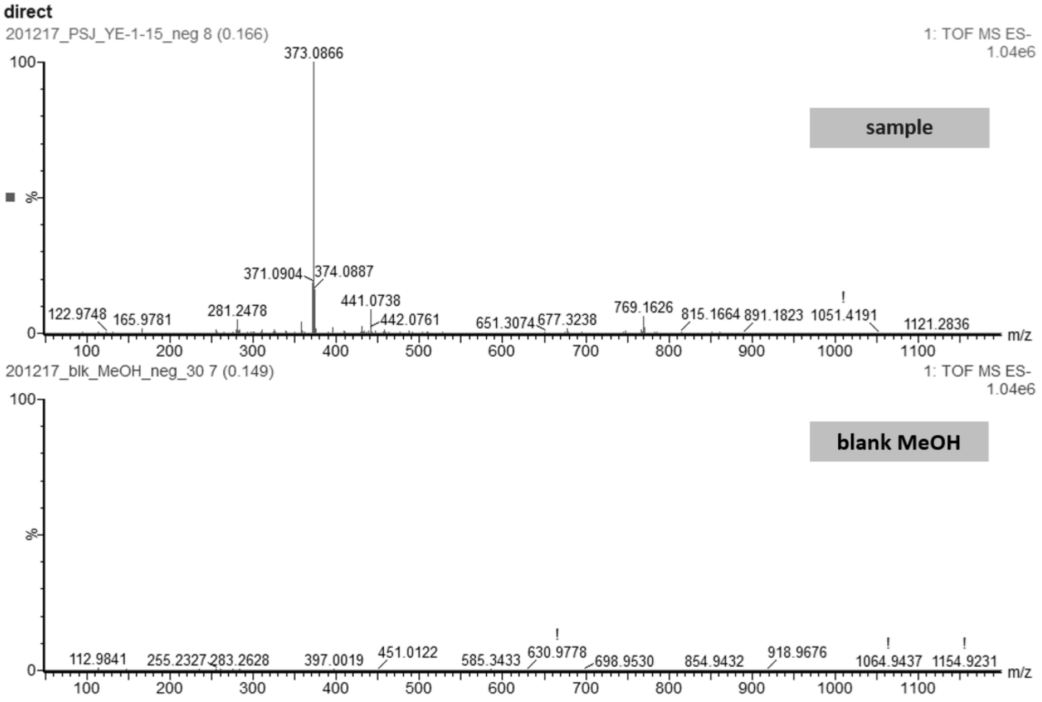

Figure S3. Molecular Docking of Compound **9a, 11a,** and **11b**

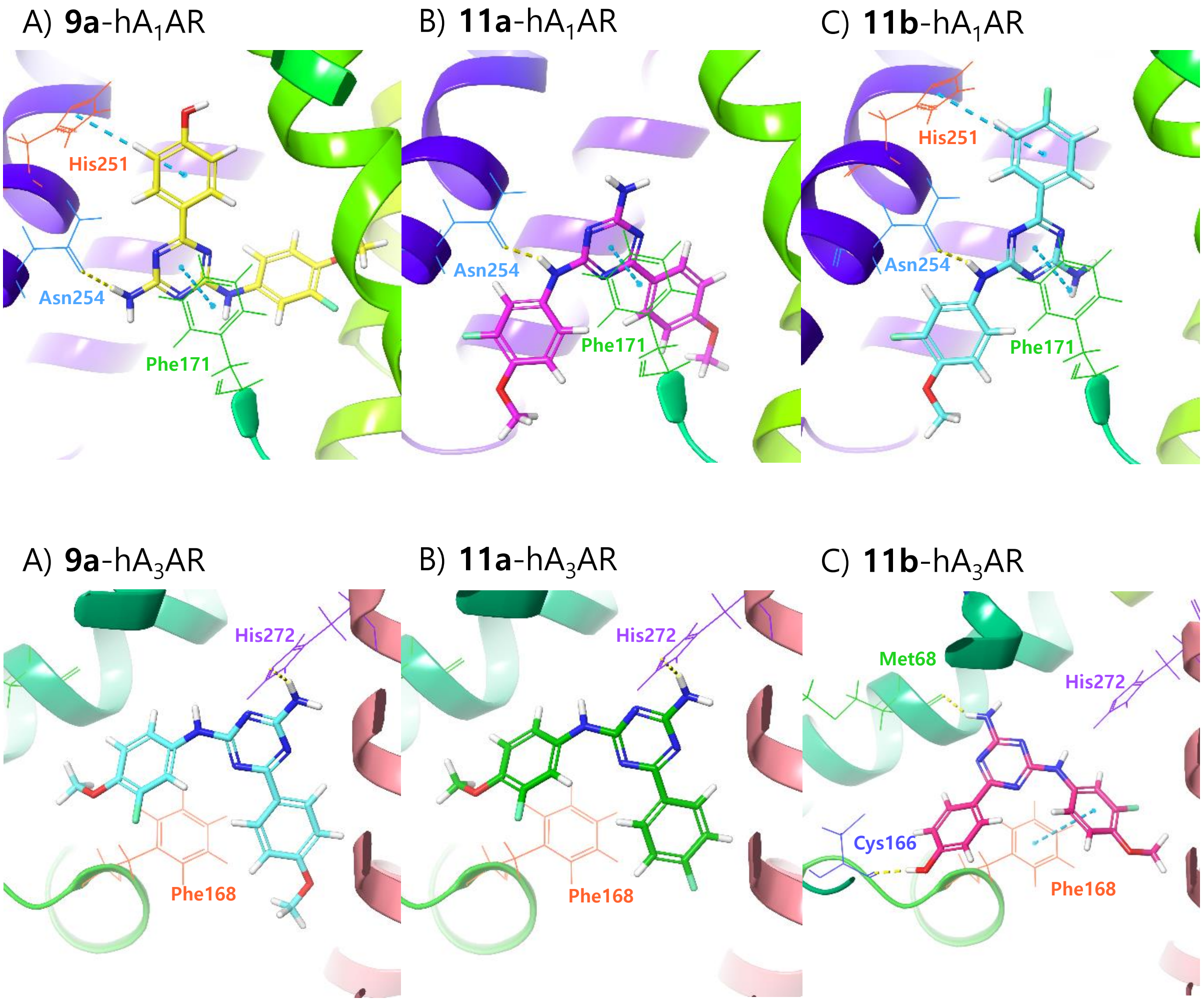

Supplement: Supplementary file 1 [file molecules-27-04016-s001.zip › molecules-1747036-supplementary.pdf]
